# Infection with the sheep gastrointestinal nematode *Teladorsagia circumcincta* increases luminal pathobionts

**DOI:** 10.1186/s40168-020-00818-9

**Published:** 2020-04-30

**Authors:** Alba Cortés, John Wills, Xiaopei Su, Rachel E. Hewitt, Jack Robertson, Riccardo Scotti, Daniel R. G. Price, Yvonne Bartley, Tom N. McNeilly, Lutz Krause, Jonathan J. Powell, Alasdair J. Nisbet, Cinzia Cantacessi

**Affiliations:** 1 Department of Veterinary Medicine, University of Cambridge, Cambridge, CB3 0ES UK; 2 Moredun Research Institute, Pentlands Science Park, Edinburgh, EH26 0PZ UK; 3 Microba, Brisbane, 4000 Australia

**Keywords:** Gastrointestinal helminth, *Teladorsagia circumcincta*, Parasite gastroenteritis, Gut microbiota, Bacterial 16S rRNA gene sequencing, Vaccine, Pathobiont, T cell, Automated in situ cell counting

## Abstract

**Background:**

The multifaceted interactions between gastrointestinal (GI) helminth parasites, host gut microbiota and immune system are emerging as a key area of research within the field of host-parasite relationships. In spite of the plethora of data available on the impact that GI helminths exert on the composition of the gut microflora, whether alterations of microbial profiles are caused by direct parasite-bacteria interactions or, indirectly, by alterations of the GI environment (e.g. mucosal immunity) remains to be determined. Furthermore, no data is thus far available on the downstream roles that qualitative and quantitative changes in gut microbial composition play in the overall pathophysiology of parasite infection and disease.

**Results:**

In this study, we investigated the fluctuations in microbiota composition and local immune microenvironment of sheep vaccinated against, and experimentally infected with, the ‘brown stomach worm’ *Teladorsagia circumcincta*, a parasite of worldwide socio-economic significance. We compared the faecal microbial profiles of vaccinated and subsequently infected sheep with those obtained from groups of unvaccinated/infected and unvaccinated/uninfected animals. We show that alterations of gut microbial composition are associated mainly with parasite infection, and that this involves the expansion of populations of bacteria with known pro-inflammatory properties that may contribute to the immunopathology of helminth disease. Using novel quantitative approaches for the analysis of confocal microscopy-derived images, we also show that gastric tissue infiltration of T cells is driven by parasitic infection rather than anti-helminth vaccination.

**Conclusions:**

*Teladorsagia circumcincta* infection leads to an expansion of potentially pro-inflammatory gut microbial species and abomasal T cells. This data paves the way for future experiments aimed to determine the contribution of the gut flora to the pathophysiology of parasitic disease, with the ultimate aim to design and develop novel treatment/control strategies focused on preventing and/or restricting bacterial-mediated inflammation upon infection by GI helminths.

Video Abstract

## Background

Gastrointestinal (GI) helminths of livestock cause serious welfare issues and are primary causes of severe economic losses worldwide due to treatment costs, stock replacement, growth retardation and impaired production [1]. Traditionally, control of GI helminths has relied upon the administration of parasiticides (= ‘anthelmintics’). However, widespread resistance to all available classes of anthelmintics in parasites of livestock [2, 3], together with the concrete threat of emerging drug resistance in human helminths [4], make the discovery and development of alternative treatment and control strategies against GI helminths a top priority.

A better understanding of the fundamental biology of parasites and of host-parasite interactions is key to the identification of potential targets for the development of novel, integrated and sustainable practices of parasite control. Thus far, the majority of studies that have attempted to unravel the complex interactions occurring at the host-helminth interface have focussed on two major players—the parasite and the host immune system (see [5] for a recent review). Nevertheless, increasing experimental evidence points towards a key role of a third party—the gut microbiota—in such interactions (reviewed by [6, 7]). Indeed, over the last few years, several investigations have demonstrated that, sharing the same environment within the vertebrate host, the GI microbiota and parasitic helminths interact with each other, and the results of these interactions may impact, directly and/or indirectly, on host health and homeostasis (reviewed by [8]). Of note, helminth infections of both humans and animal models have been associated with significant alterations in the relative abundances of several gut microbial taxa with immune-modulatory functions (e.g. [9–16]). This raises the question of whether parasite-associated qualitative and quantitative modifications in gut microbiota, and of its metabolism, may represent mechanisms by which helminths establish chronic infections [9]. However, whether such modifications are caused by direct interactions of the microbial flora with helminth parasites and/or, indirectly, by changes in the mucosal immune environment, remains to be established. Determining relationships between GI helminths, the host immune system and the gut microbiota is nonetheless pivotal, as this new knowledge will form the basis for the development of targeted parasite control strategies based on the manipulation of these interactions. One host-parasite pair in which such investigations are possible is the sheep-*Teladorsagia* system.

*Teladorsagia circumcincta* is the most prevalent nematode parasite of sheep in the UK, and one of the leading causes of parasite gastroenteritis (PGE) in temperate areas worldwide [17]. This parasite is transmitted through the faecal-oral route and develops in the abomasum of the ovine host, where it causes pathology with mucosal damage and a protein-losing gastropathy associated with host inflammatory immune responses [18]. *T*. *circumcincta* resistance to anthelmintics is widespread [19, 20], thus making control of this parasite in flocks highly challenging. Recent efforts by Nisbet and colleagues [21–23] have resulted in the development of an effective sub-unit vaccine against *T*. *circumcincta*, composed of a cocktail of eight recombinant proteins [21–23]. Administration of this vaccine to 6–7-month-old lambs resulted in up to 70% and 75% reduction in total worm and faecal egg counts (FEC) (the latter a proxy of parasite burden), respectively, compared to unvaccinated/challenged controls, thus representing a promising alternative to the administration of anthelmintics [21]. The availability of this system, where vaccination is effective at reducing worm numbers but does not induce sterile immunity, represents an opportunity to gain a better understanding of the relationships between GI helminths, gut microbiota and host immune responses. Thus, in this study, we provide insights into these three-fold interactions *via* the characterisation of the fluctuations in gut microbiota composition and relative abundance of individual microbial species in sheep (i) following experimental infection with *T*. *circumcincta*, and (ii) following immunisation and parasite challenge, and establish links between these alterations and immune microenvironment features using immunofluorescence labelling and quantitative microscopy. We show that modifications in gut flora composition are driven primarily by parasite infection, rather than by vaccine-induced immunity, and identify bacterial taxa with likely roles in the immunopathology of helminth infection.

## Materials and methods

### Experimental procedures

**Fig. 1:**
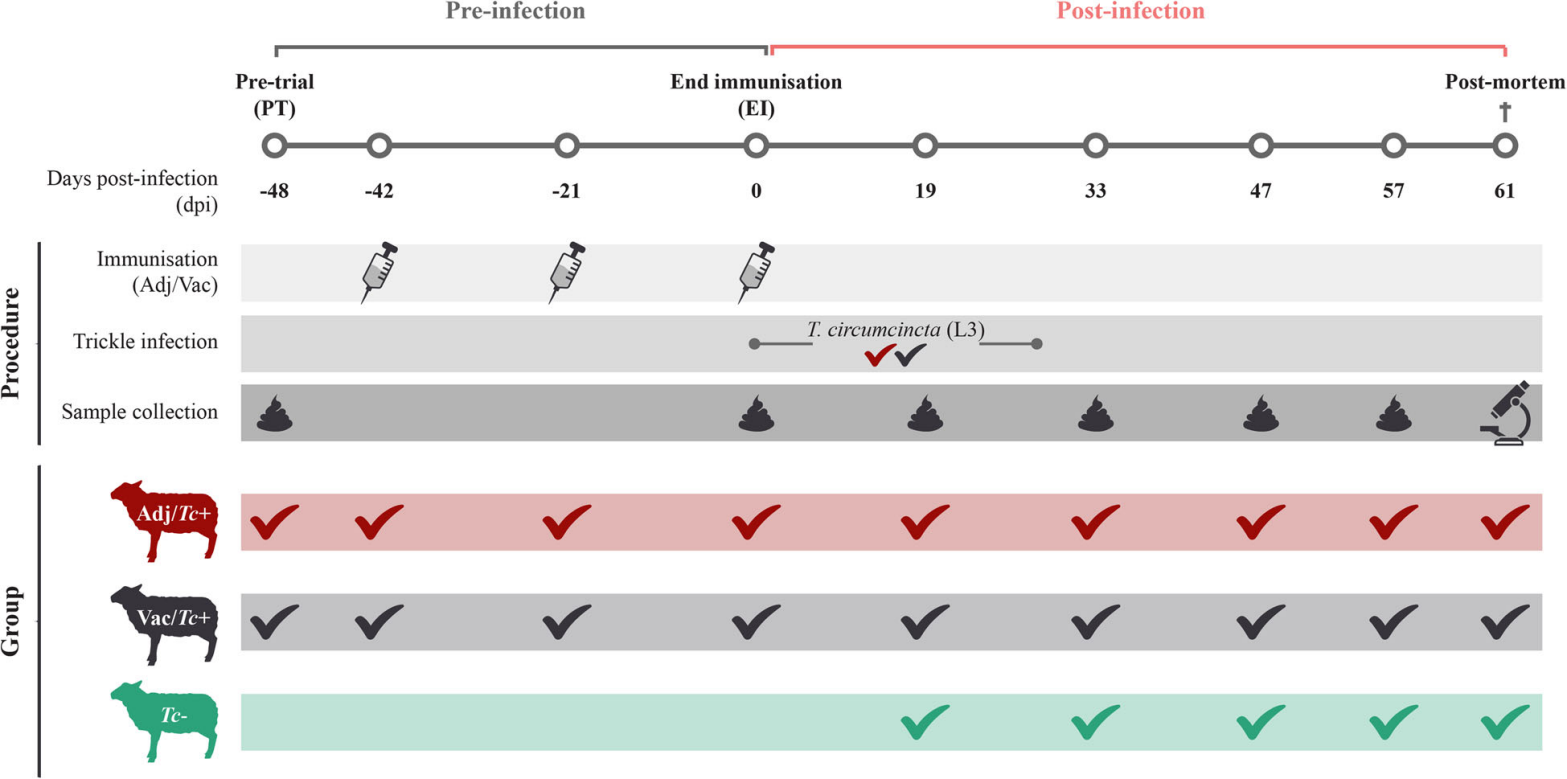
Experimental design. Schematic representation of the timeline of the study and the experimental procedures conducted on sheep infected with *Teladorsagia circumcincta* following adjuvant (Adj/*Tc*+) or vaccine (Vac/*Tc*+) administration, and uninfected controls (*Tc*-)

Thirty Texel crossbred lambs, 5 to 6 months of age, reared under helminth-free conditions (verified and confirmed by parasitological examination of individual faecal samples prior to the beginning of the study) were randomly divided into three age- and gender-balanced groups of ten lambs each. Each group (groups 1–3) was housed in a separate pen. In alignment with 3Rs principles, lambs in groups 1 and 2 were derived from a vaccine efficacy study recently published by Nisbet and co-workers [23] and correspond to the animals described in the synonymous groups enrolled in trial 6 of said study. Briefly, lambs in group 1 (‘Vac/*Tc+*’) were injected three times with a recombinant vaccine against *T*. *circumcincta*, leaving a 3-week interval between successive injections; protocols of vaccine production and administration had been described previously [21]. Following the final immunization, each animal was experimentally infected with 2000 *T*. *circumcincta* infective third-stage larvae (L3), administered orally three times per week for 4 weeks [21] (Fig. 1). Lambs in group 2 (‘Adj/*Tc*+’) were injected with the vaccine vehicle (i.e. urea, PBS and the adjuvant Quil A) [21] and infected with *T*. *circumcincta* larvae as described for animals in group 1 (Fig. 1). Lambs in group 3 (negative controls; ‘*Tc*-’) were included with the primary objective to assess the effect of vaccination-induced reduction in worm numbers on the dynamics of faecal microbiome composition, and remained unimmunized and uninfected until the end of the experiment (Fig. 1). All lambs were euthanised at the end of the trial, i.e. 103 days from the first immunisation, for total worm counts and sample collection for automated in situ cell counting analyses (see below).

### Parasitological analyses and sample collection for microbiome sequencing

In order to account for the parasite pre-patent period, individual faecal samples were collected directly from the rectum of each sheep 12 days after the first *T*. *circumcincta* trickle infection (dpi) in both Vac/*Tc+* and Adj/*Tc*+ groups, and twice weekly thereafter, for parasitological analyses. FEC were performed twice a week using the salt flotation technique, sensitive to one egg per gram [24] for estimation of parasite burdens during the timeframe of the trial. At the end of the trial, worms were enumerated from the abomasa of each sheep using standard techniques [25]. A Generalised Additive Mixed Modelling (GAMM) approach was undertaken for analysis of longitudinal FEC data, whilst cumulative FEC values for each sheep (cFEC) were calculated using the trapezoidal method, as previously described [23]. Worm burden data were analysed using negative binomial generalised linear models with a logarithmic link function accounting for data over-dispersion as described previously [21–23].

Individual faecal samples for microbiome sequencing were collected from all lambs in the Vac/*Tc+* and Adj/*Tc*+ groups 6 days prior to the first immunization (pre-trial = PT), on the day of the last immunization (end of immunization = EI) and at 19, 33, 47 and 57 dpi (Fig. 1) and stored in sterile containers. Individual faecal samples were also collected from *Tc*- lambs at 19, 33, 47 and 57 days after the first trickle infection of lambs in the Vac/*Tc+* and Adj/*Tc*+ groups (Fig. 1). Samples were stored at − 80 °C immediately after collection and until use for DNA extraction.

### DNA extraction and high-throughput sequencing of the bacterial 16S rRNA gene

An equal number of samples collected from each experimental group and at different time points was processed simultaneously to minimise technical sources of variation (batch effect). Genomic DNA was extracted from a total of 154 faecal samples, as well as seven (no-DNA template) negative controls, using the PowerSoil DNA Isolation Kit (Qiagen) according to manufacturers’ instructions. Sequencing libraries were prepared following Illumina recommended instructions for bacterial 16S rRNA amplicon sequencing library preparation, with minor adjustments. Briefly, the V3–V4 region of the bacterial 16S rRNA gene was amplified by conventional PCR using universal primers [26], that contained the Illumina adapter overhang nucleotide sequences, Q5® NEBNext Hot Start High-Fidelity DNA Polymerase (New England Biolabs), 5 ng/μl of template DNA and the following thermocycling protocol: 98 °C for 2 min, 20 cycles of 98 °C/15 s—63 °C/30 s—72 °C/30 s and a final elongation step at 72 °C for 5 min. Amplicons were purified using AMPure XP beads (Beckman Coulter) and set up for the index PCR using Q5® NEBNext Hot Start High-Fidelity DNA Polymerase (New England Biolabs) and Nextera XT index primers (Illumina) with thermocycling conditions as follows: 3 min at 95 °C, 8 cycles of 30 s at 95 °C—30 s at 55 °C—30 s at 72 °C and 5 min at 72 °C. The indexed samples were purified using AMPure XP beads (Beckman Coulter), quantified using the Qubit™ dsDNA High Sensitivity Kit (Life Technologies) and equal DNA amounts from each sample were pooled. The resulting pooled library was quantified using the NEBNext® Library Quantification Kit for Illumina® (New England Biolabs), and sequenced using the v3 chemistry (2x300 bp paired-end reads, Illumina).

### Bioinformatics and statistical analyses

Paired-end demultiplexed Illumina sequencing reads were imported into the Quantitative Insights Into Microbial Ecology 2 (QIIME2; 2018.6 distribution, https://qiime2.org) software suite [27]. Sequences were then quality filtered, dereplicated, chimeras identified and paired-end reads merged in QIIME2 using DADA2 [28] with default settings. A phylogenetic tree was generated using the align-to-tree-mafft-fasttree pipeline in the q2-phylogeny plugin. Bray-Curtis dissimilarity between samples was calculated using core-metrics-phylogenetic method from the q2-diversity plugin. Classification of Operational Taxonomic Units (OTUs) was performed using a Naïve Bayes algorithm trained using sequences representing the bacterial V3–V4 rRNA region available from the SILVA database (https://www.arb-silva.de/download/archive/qiime; Silva_132) [29], and the corresponding taxonomic classifications were obtained using the q2-feature-classifier plugin in QIIME2. The classifier was then used to assign taxonomic information to representative sequences of each OTU. Statistical analyses were executed using the Calypso software (cgenome.net/calypso/) [30]. For data normalisation, Cumulative-Sum Scaling (CSS) was applied to the OTU table, followed by log2 transformation to account for the non-normal distribution of taxonomic count data. Samples were clustered using unsupervised Principal Coordinates Analyses (PCoA) based on Bray-Curtis dissimilarities. Supervised Canonical Correlation Analyses (CCA), Redundancy Analyses (RDA) and permutational multivariate analyses of variance using distance matrices (Adonis) [31] were performed to investigate significant associations between microbiota composition and infectious status, administration of Vac or Adj, time point and experimental group. Adonis was run on Bray-Curtis dissimilarity matrices. Rarefaction was applied before analysis of microbial alpha- and beta diversity to account for differences in sample sequencing depth. Changes in microbial alpha diversity (Shannon index) over the course of the experiment were evaluated using Mixed Effect Linear Regression (MELR), whilst ANOVA was employed to test differences between groups at each time point. Beta diversity was calculated using Bray-Curtis dissimilarity, and differences across time points and between experimental groups were evaluated using Analysis Of Similarity (ANOSIM) [32]. Pairwise comparisons of beta diversity were performed by Permutational Multivariate Analysis Of Variance (PERMANOVA) [31] using the beta-group-significance function from the q2-diversity plugin within QIIME2. Longitudinal changes in the abundance of individual taxa in the faecal microbiota of each lamb were evaluated using MELR analysis followed by Tukey’s test as a post hoc analysis [33] and *p* values were corrected for multiple testing by False Discovery Rate (FDR) with a 0.05 significance level. Differences in the microbiota composition between groups were assessed at each time point using the Linear Discriminant analysis Effect Size (LEfSe) workflow [34] and negative binomial distribution (DESeq2) [35], the latter applied on not normalised, not rarefied datasets; DESeq2 *p* values were corrected by FDR and *q* values under 0.05 were considered statistically significant.

### Tissue sample collection, immunofluorescence labelling and imaging

At the end of the trial, sections (approximately 2 × 2 cm in size) were collected from the fundus of the abomasa of a subset of sheep enrolled in this study. In particular, to achieve a wide range of cFEC for correlation, animals displaying high (generally in the Adj/*Tc*+ group) and low (generally in the Vac/*Tc*+ group) cFEC, with overlapping edges between groups, were selected for immunofluorescence labelling and automated, in situ cell counting. Tissues from five Vac/*Tc*+ animals with an average cFEC of 2068 (± 918) and five Adj/*Tc*+ animals with an average cFEC of 8061 (± 1840) were selected (Additional file 1). Sections from these tissues were subjected to immunofluorescence labelling and imaging. Briefly, following sectioning, tissues were snap-frozen in isopentane (pre-cooled in liquid nitrogen) for 1 min, immediately transferred into individual collection tubes and stored at − 80 °C until further use.

For immunofluorescence labelling, frozen tissues were embedded in optimal cutting temperature (OCT) compound, sectioned at 12 μm, collected onto SuperFrost® slides (Thermo Scientific) and left to air dry for 1 h at room temperature. After re-hydration in PBS for 30 min, sections were fixed with 4% paraformaldehyde (Sigma-Aldrich) for 10 min, and then washed three times (15 min each) in Tris-buffered saline (TBS) (Sigma-Aldrich) containing 25 mM glycine. After washing, fixed sections were incubated for 30 min in TBS containing 10% (v/v) goat serum, 1% (w/v) BSA and 25 mM glycine to block non-specific binding of antibodies.

For T lymphocyte counts in abomasal mucosa and epithelium, sections were incubated for 1 h with polyclonal rabbit anti-human CD3 (cross reactive with sheep, A045229, Agilent), followed by four washes with TBS (3 min each) and 1h incubation with Alexa Fluor® 488 goat anti-rabbit IgG [H+L] (A11034, Thermo Fisher Scientific). Following three further washes with TBS, nuclei were counterstained with TO-PRO™-3 Iodide for 15 min (T3605, Thermo Fisher Scientific). Alongside, a rabbit anti-human polyclonal isotype control (ab37415, Abcam) was used in tissue-matched serial cryostat sections to control for non-specific staining of the anti-CD3 antibody.

Finally, sections were mounted with #1.5 coverslips using VECTASHIELD HardSet Antifade Mounting Medium (Vector Laboratories) and imaged with a Leica TCS SP5 confocal microscope (Leica Microsystems) using 488 nm and 633 nm lasers to excite the AlexaFluor® 488 and TO-PRO™-3 iodide respectively. Images were collected using a × 20, 0.7 NA multi-immersion lens. Data were recorded using the Leica Confocal Software (v2.61).

### Quantitative analysis

Automated, in situ cell counting was performed using the freely available CellProfiler software (www.CellProfiler.org). Screenshots of all 40 modules used in the complete image analysis pipeline are provided in Additional file 2, and example raw image data from the confocal microscope, alongside the CellProfiler pipeline are also directly available *via* the BioStudies database under accession code S-BSST263. In brief, image data were loaded directly from the raw Leica .LIF files, and were thresholded on the basis of tissue-matched serial cryostat sections exposed to the secondary antibody alone (i.e. secondary-only controls). A mask of the nuclei in each image was then created to define the region of each field-of-view that contained mucosal tissue (i.e. to avoid illumination correction calculation on ‘blank’ regions of the image). For the tissue-occupied region, an illumination correction function was calculated to compensate for any unevenness in illumination resulting from tissue section curvature relative to the optical section of the confocal. Once calculated, this function was used to correct both the nuclei (i.e. TO-PRO™-3 iodide) and CD3 (i.e. AlexaFluor® 488) images. The TO-PRO™-3 iodide image was then intensity rescaled prior to segmentation of each nuclei using the ‘IdentifyPrimaryObjects’ module. Each nuclei-object was then dilated 5 pixels to create an integration contour wide enough from the parent nucleus to capture any associated CD3 staining. The size and shape of each cell-object defined by the integration contour was then measured, as well as the per-object fluorescence intensity in every channel. Data were exported as MATLAB objects. As is recommended practice for image-based cell profiling [36], cell objects outside the 5th and 95th percentiles by size were discarded prior to determining T lymphocyte counts on the basis of integrated, per-cell fluorescence intensities with values greater than those observed in tissue-matched, isotype control images (i.e. as per flow cytometry).

Worm recoveries, cFEC and CD3^+^ cell count datasets were tested for normality by Kolmogorov-Smirnov test and correspondence between parasite burdens (i.e. worm recoveries and cFEC) and the percentage of CD3^+^ cells in the abomasa of infected animals was tested by Spearman’s and Pearson’s correlations, for worm recoveries and cFEC, respectively.

## Results

### Experimental infection outcomes

Faecal examination confirmed that all animals exposed to *T*. *circumcincta* larvae were successfully infected and remained free of other undesired helminth infections; in turn, uninfected controls were verified to remain helminth-free until the end of the experiment. FEC profiles in Adj/*Tc*+ and Vac/*Tc*+ sheep over the course of the experiment are shown in Additional file 3. *T*. *circumcincta* eggs were detected in the faeces of parasite-exposed sheep from 15 dpi until the end of the trial, and GAMM analysis identified statistically significant differences in patterns of mean FEC between Adj/*Tc*+ and Vac/*Tc*+ sheep over the time course of the experiment (*p* = 0.044) [21]. An overall reduction in median FEC was observed in Vac/*Tc*+ animals compared with the Adj/*Tc*+ group over the course of the experiment (Additional file 3a and b). In addition, Vac/*Tc*+ sheep had, on average, 66% fewer *T*. *circumcincta* eggs per gram of faeces at the point of peak egg shedding than Adj/*Tc*+ sheep and mean cFEC and worm burdens at post mortem showed overall reductions of 30% and 50% in vaccinated *versus* adjuvant-inoculated sheep. Mean (± SEM) and median worm counts in the Vac/*Tc*+ group were 1033 (± 349, *n* = 9) and 600, respectively, and mean (± SEM) and median worm counts in Adj/*Tc*+ sheep were 2050 (± 536, *n* = 10) and 1900, respectively (Additional file 3c). Mean worm burdens in the five sheep selected for immunostaining and imaging in the Vac/*Tc*+ group were 740 ± 472 (median value 200) and, in the Adj/*Tc*+ group, 1840 ± 627 (median value 2500) (Additional file 1). One lamb in the Vac/*Tc*+ group failed to thrive throughout the experiment and, at post-mortem examination, displayed evidence of necrotic liver tissue. Data generated from samples collected from this animal were therefore excluded from downstream analyses.

### Microbiota profiling

A total of 30,434,702 paired-end reads were generated from 154 faecal extracts collected from 29 animals at different time points (Fig. 1), and subjected to further processing. After primer trimming, quality filtering, de-noising and removal of chimeric sequences, a total of 7,927,181 high-quality sequences (per sample mean 51,475 ± 3402) were retained for OTU table construction and selection of representative sequences. Raw sequence data generated in this study are available from the European Nucleotide Archive (ENA) database under accession number PRJEB33114. Rarefaction curves generated following data curation showed that the majority of the bacterial communities were represented in the retained sequences (Additional file 4). These sequences were assigned to 21,704 OTUs, 18 bacterial phyla and one archaeal phylum. The overall bacterial profile of all samples included in the study is shown in Additional file 5. The phyla *Firmicutes* and *Bacteroidetes* were predominant in all samples analysed (40.44% mean ± 0.34% standard deviation and 36.01 ± 0.33%, respectively), followed by the phyla *Verrucomicrobia* (5.53 ± 0.15%), *Spirochaetes* (4.0 ± 0.10%), *Cyanobacteria* (2.64 ± 0.07%), *Proteobacteria* (2.32 ± 0.07%), *Fibrobacteres* (1.71 ± 0.10%), *Lentisphaerae* (1.66 ± 0.05%), *Euryarchaeota* (1.53 ± 0.74%), *Tenericutes* (1.14 ± 0.04%), *Epsilonbacteraeota* (0.98 ± 0.07%), *Planctomycetes* (0.65 ± 0.02%) and *Patescibacteria* (0.47 ± 0.03%). The remaining phyla, including *Kiritimatiellaeota*, *Elusimicrobia*, *Actinobacteria*, *Deferribacteres*, *Chloroflexi* and *WPS*-*2*, composed less than 1% of the whole microbiota (Additional file 5). Predominant lower level bacterial taxa were class *Clostridia*, order *Clostridiales* and family *Ruminococcaceae* within the phylum *Firmicutes*, and class *Bacteroidia*, order *Bacteroidales* and family *Rikenellaceae* within the phylum *Bacteroidetes* (Additional file 5).

### Changes in faecal microbiota composition of Adj/*Tc*+, Vac/*Tc*+ and *Tc*− over the course of the experiment

**Fig. 2:**
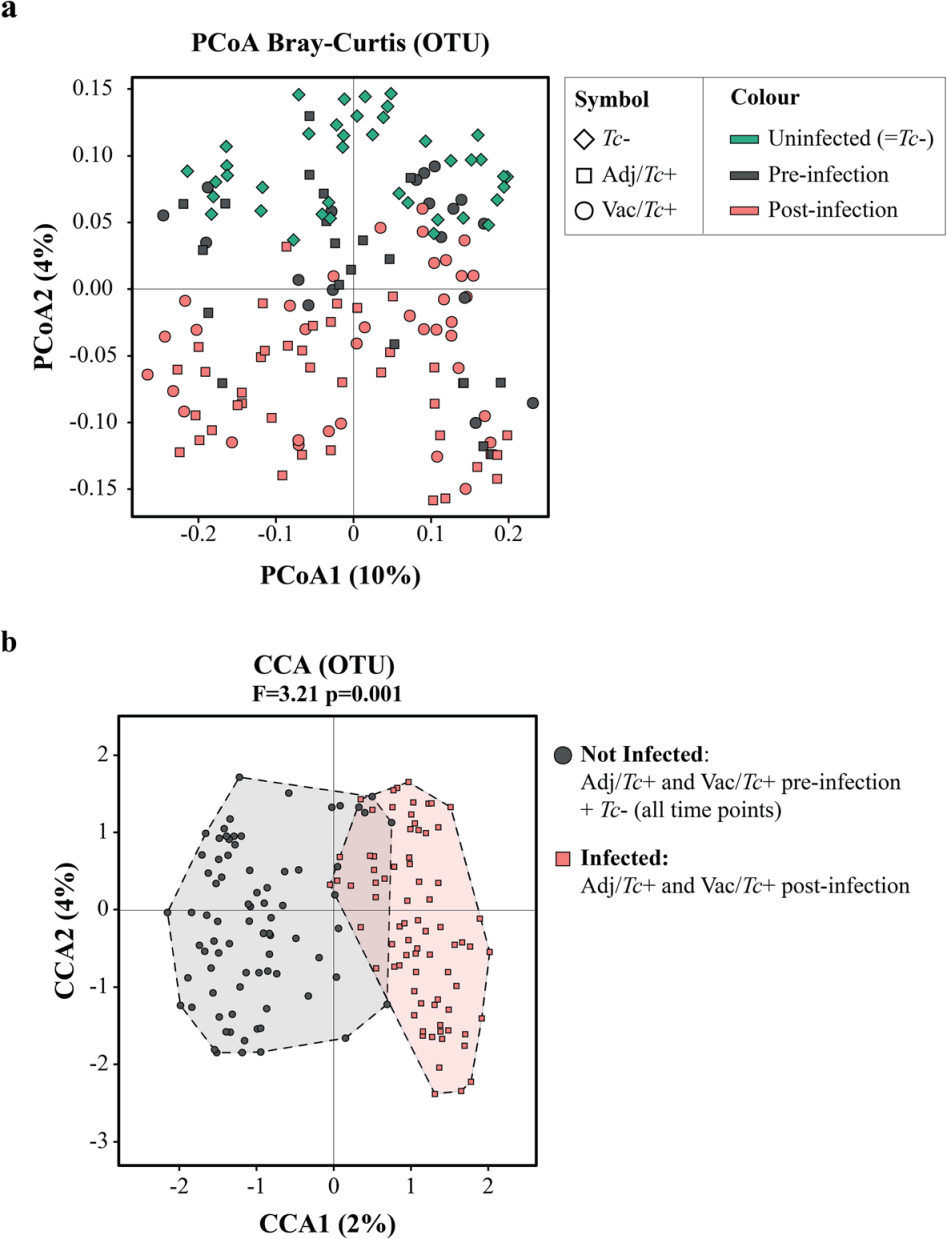
*Teladorsagia circumcincta* impacts on the faecal microbiota composition of sheep. Multivariate statistical analyses applied to the faecal microbiota of sheep infected with *T*. *circumcincta* following adjuvant (Adj/*Tc*+) or vaccine (Vac/*Tc*+) administration, and uninfected controls (*Tc*−). **a** Principal coordinate analysis (PCoA) for all the samples in the study, clustered according to experimental group (symbol) and infection status (colour). **b** Canonical Correlation Analysis (CCA) of microbial profiles of all samples, clustered according to infection status

Faecal microbial community profiles obtained from individual samples were ordinated by supervised and unsupervised multivariate analyses (Fig. 2). In particular, microbial communities clustered by infection status using unsupervised PCoA on Bray-Curtis dissimilarity along principal coordinate 2, with the vast majority of samples collected from Adj/*Tc*+ and Vac/*Tc*+ prior to experimental infection and samples from *Tc*− grouping together to the exclusion of post-infection Adj/*Tc*+ and Vac/*Tc*+ samples (Fig. 2a). This clustering was supported by supervised CCA (*p* ≤ 0.001; results for CCA are shown in Fig. 2b, whilst the full list of results from multivariate statistical analyses is provided in Additional file 6a).

No significant fluctuations in OTU alpha diversity, calculated by Shannon index, were detected within any of the experimental groups over time (Additional file 7a). However, statistically significant differences in faecal microbial beta diversity were detected by ANOSIM (Bray-Curtis dissimilarities) within Adj/*Tc*+ and Vac/*Tc*+ over time, but not in *Tc*−, albeit with low *R* values (Additional file 8).

**Fig. 3:**
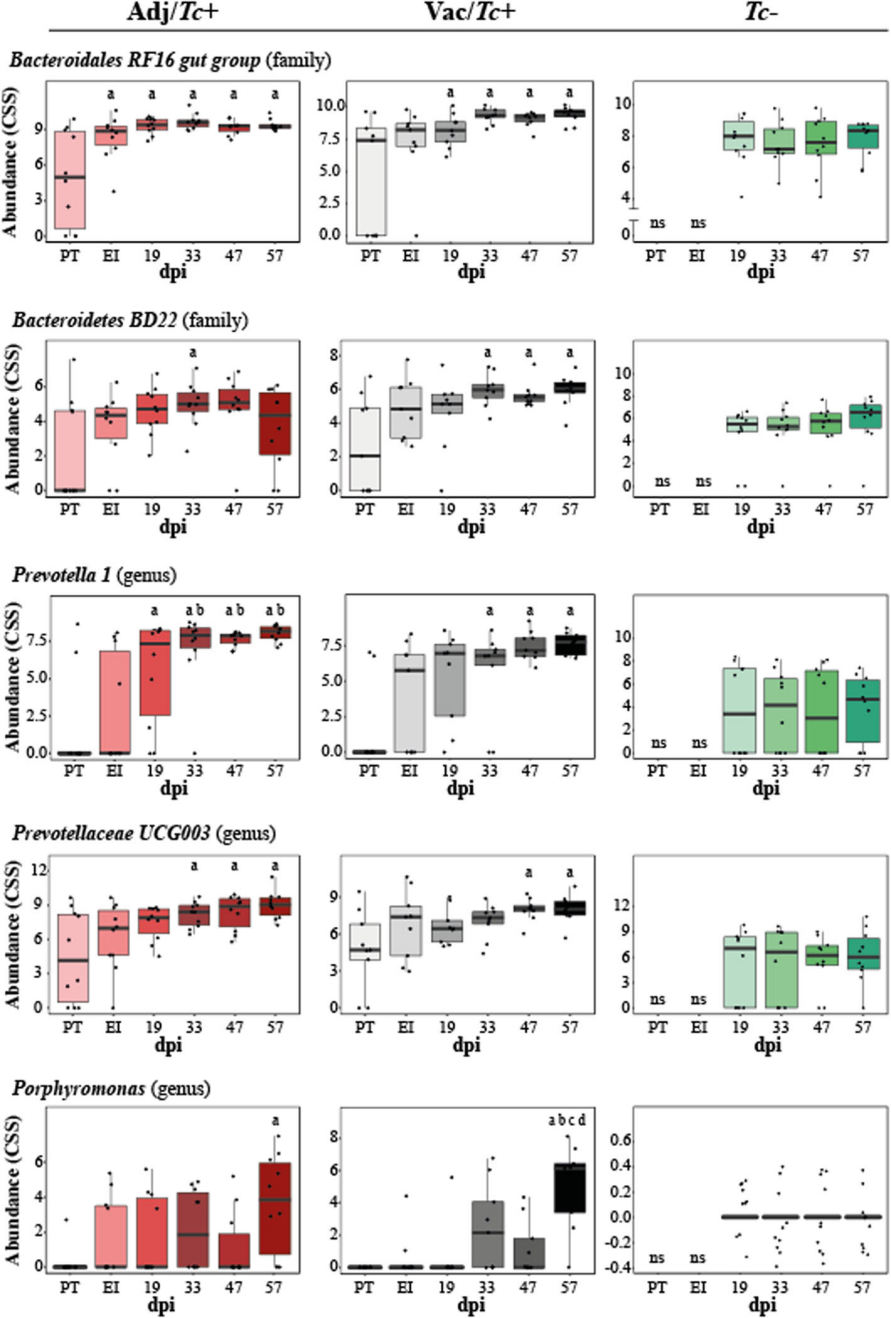
Infection is associated with longitudinal changes in taxa abundances. Boxplots representing overtime profiles of microbial taxa significantly altered in the faeces of sheep infected with *Teladorsagia circumcincta* following adjuvant inoculation (Adj/*Tc+*) or vaccination (Vac/*Tc+*) and uninfected controls (*Tc*−). Statistical differences were assessed by Mixed Effect Linear Regression (FDR-adjusted *q* < 0.05) followed by Tukey’s test as a *post hoc* analysis; letters indicate significant differences (Tukey’s *p* < 0.05) for a particular time point with respect to : a = pre-trial (PT); b = end of immunisation (EI); c = 19 days post infection (dpi) and d = 47 dpi. *ns* = no sample available

Changes in the relative abundances of individual microbial taxa in the faecal microbiota of Adj/*Tc*+, Vac/*Tc+* and *Tc*− over the course of the experiment were assessed by MELR (FDR-adjusted cut-off *q* < 0.05; Additional file 9). In particular, the family *Bacteroidetes* BD22, as well as the genera *Prevotella* 1 and *Prevotellaceae* UCG003, were progressively and significantly expanded in the faecal microbiota of Adj/*Tc*+ and Vac/*Tc+* sheep following trickle infection (Fig. 3). Populations of *Bacteroidales* RF16 group were expanded in the faecal microbiota of both groups of infected animals following adjuvant/vaccine inoculation, albeit significant differences in the abundance of this taxon with respect to PT samples were only recorded in the Adj/*Tc*+ group (Fig. 3). Additionally, the family *Porphyromonadaceae* and the genus *Porphyromonas* were significantly increased in the faecal microbiota of both experimental groups at the latest time point when compared with PT samples (Fig. 3).

Within the Adj/*Tc+* group, bacteria belonging to the family *Bifidobacteriaceae* and the genus *Bifidobacterium*, and to the family *Burkholderiaceae* and the genus *Sutterella*, were progressively expanded over time (MELR; FDR-adjusted *q* < 0.05); however, no significant differences between the abundances of these taxa were detected between time-point pairs using *post hoc* analysis (Additional file 10). Of the microbial communities identified in samples from Vac*/Tc*+, populations of bacteria belonging to the family *Burkholderiaceae* and genus *Sutterella* were also expanded following experimental infection (MELR; *p* < 0.05), although statistical significance was not maintained following FDR correction for multiple testing (Additional file 10). Furthermore, bacteria belonging to the family *PeH15* were significantly expanded in Vac*/Tc*+ samples from EI onwards, along with *Endomicrobiaceae* (family) and *Candidatus Endomicrobium* (genus) (FDR-adjusted *q* < 0.05), albeit for the latter two taxa, significant differences were not detected between any pair of time points by *post hoc* analysis (Additional file 10).

### Differences in faecal microbial composition between Adj/*Tc*+, Vac/*Tc*+ and *Tc*−

Significant differences between the microbial profiles of samples collected from Adj/*Tc*+, Vac/*Tc*+ and *Tc*− sheep were detected at each time point post-challenge infection (*p* < 0.05; results for CCA are shown in Additional file 11a, whilst the full list of results from multivariate statistical analyses is provided in Additional file 6b). In order to determine significant associations between gut microbial profiles and Adj/Vac immunisation and/or helminth infection, the faecal microbial compositions of Adj/*Tc*+ and Vac/*Tc*+ were compared at each time point. Statistically significant differences between the gut microbiota of these two groups were observed at 57 dpi, thus suggesting a significant association between faecal microbial composition and Adj/Vac administration at this time point (CCA, RDA and Adonis *p* < 0.05; Additional files 6b and 11b). For the remaining time points post-trickle infection (i.e. 19, 33 and 47 dpi), no statistically significant differences were observed between the faecal microbial profiles of Adj/*Tc*+ and Vac/*Tc*+. Thus, the effect of helminth infection on faecal microbial composition was assessed by pooling samples from Adj/*Tc*+ and Vac/*Tc*+ animals into a single group (i.e. ‘infected’) for comparative analyses with samples from ‘uninfected’ (i.e. *Tc*−) animals. A significant association between faecal microbiota composition and infection status was detected by CCA at each time point (*p* < 0.05; Additional files 6b and 11c). Conversely, no significant differences in OTU alpha diversity (Shannon index) were detected between samples collected from Adj/*Tc*+, Vac/*Tc*+ and *Tc*− at any time point (Additional file 7b).

Differences in the relative abundance of individual bacterial taxa (from phylum to genus level) were detected by LEfSe between each pair of experimental groups at every time point (LDA score (log10) > 2.5; Additional file 12). Alterations significantly associated with *T*. *circumcincta* infection irrespective of vaccine/adjuvant administration included expanded populations of *Porphyromonas* (family *Porphyromonadaceae*), *Sutterella* (family *Burkholderiaceae* and order *Betaproteobacterales*), *Candidatus Saccharimonas* (family *Saccharimonadaceae*, order *Saccharimonadales*, class *Saccharimonadia* and phylum *Patescibacteria*) and *Bacteroidales* RF16 group from 19 dpi onwards (Table 1). Furthermore, several genera within the *Prevotellaceae* family, including *Prevotella* 1 and *Prevotellaceae* UCG003, amongst others, were significantly more abundant in the faecal microbiota of Adj/*Tc*+ and Vac/*Tc*+ compared with that of *Tc*− animals from 33 dpi (Table 1). Differences in the abundance of *Prevotella* 1, *Porphyromonas* (and corresponding family) and *Candidatus Saccharimonas* (and corresponding family, class and phylum) and *Bacteroidales* RF16 group, observed between the faecal microbiota of infected and uninfected animals, were confirmed by DESeq2 (FDR-adjusted *q* < 0.05) at the end of the experiment (57 dpi) (Additional files 13a and 14a). Using the same method, expanded populations of *Proteobacteria* (phylum) and *Gammaproteobacteria* (class) (FDR-adjusted *q* < 0.05) were detected in the faecal microbiota of infected sheep compared with the uninfected counterparts; nevertheless, differences in the abundances of *Betaproteobacteriales* (order), *Burkholderiaceae* (family) and *Sutterella* (genus) detected by DESeq2 (*p* < 0.05) were not maintained following FDR correction for multiple testing (Additional file 14b). In addition, LEfSe analysis detected expanded populations of *PeH15* (family) in the microbiota of Vac/*Tc*+ animals compared to the unvaccinated groups from EI onwards (Additional file 12), a result that was confirmed by DESeq2 (FDR-adjusted *q* < 0.001) at 33, 47 and 57 dpi (Additional files 13b and 14). The full list of microbial taxa whose relative abundances differed between the faecal microbiota of infected *vs*. uninfected, and vaccinated *vs*. unvaccinated animals by DESeq2 at different time points is available from Additional file 14 a and b. None of the differentially abundant bacterial taxa between infected and uninfected, and vaccinated and unvaccinated animals, differed in abundance between Adj/*Tc+* and Vac/*Tc+* (i.e. infected), nor between Adj/*Tc+* and *Tc*− (i.e. unvaccinated), respectively, using this method (Additional file 14c and d ).

**Table 1:**
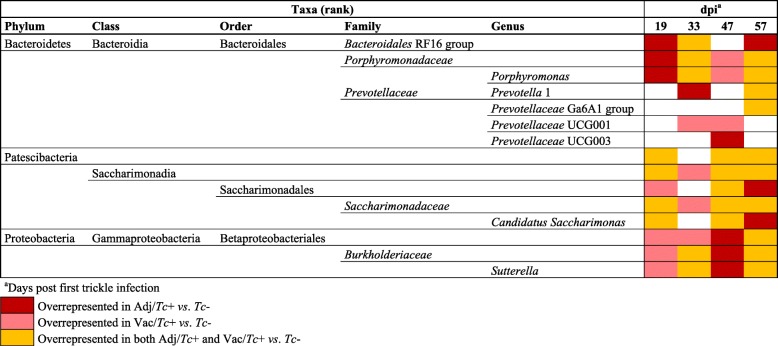
Selected microbial taxa displaying significantly higher abundance in faecal samples from sheep experimentally infected with *Teladorsagia circumcincta* following adjuvant (Adj/*Tc*+) or vaccine (Vac/*Tc*+) administration, compared to uninfected controls (*Tc*−). Results based on Linear discriminant analysis Effect Size (LEfSe); LDA score (log10) > 2.5

### Correlation between abomasal T lymphocyte frequency and infection burden or faecal microbiota composition

**Fig. 4:**
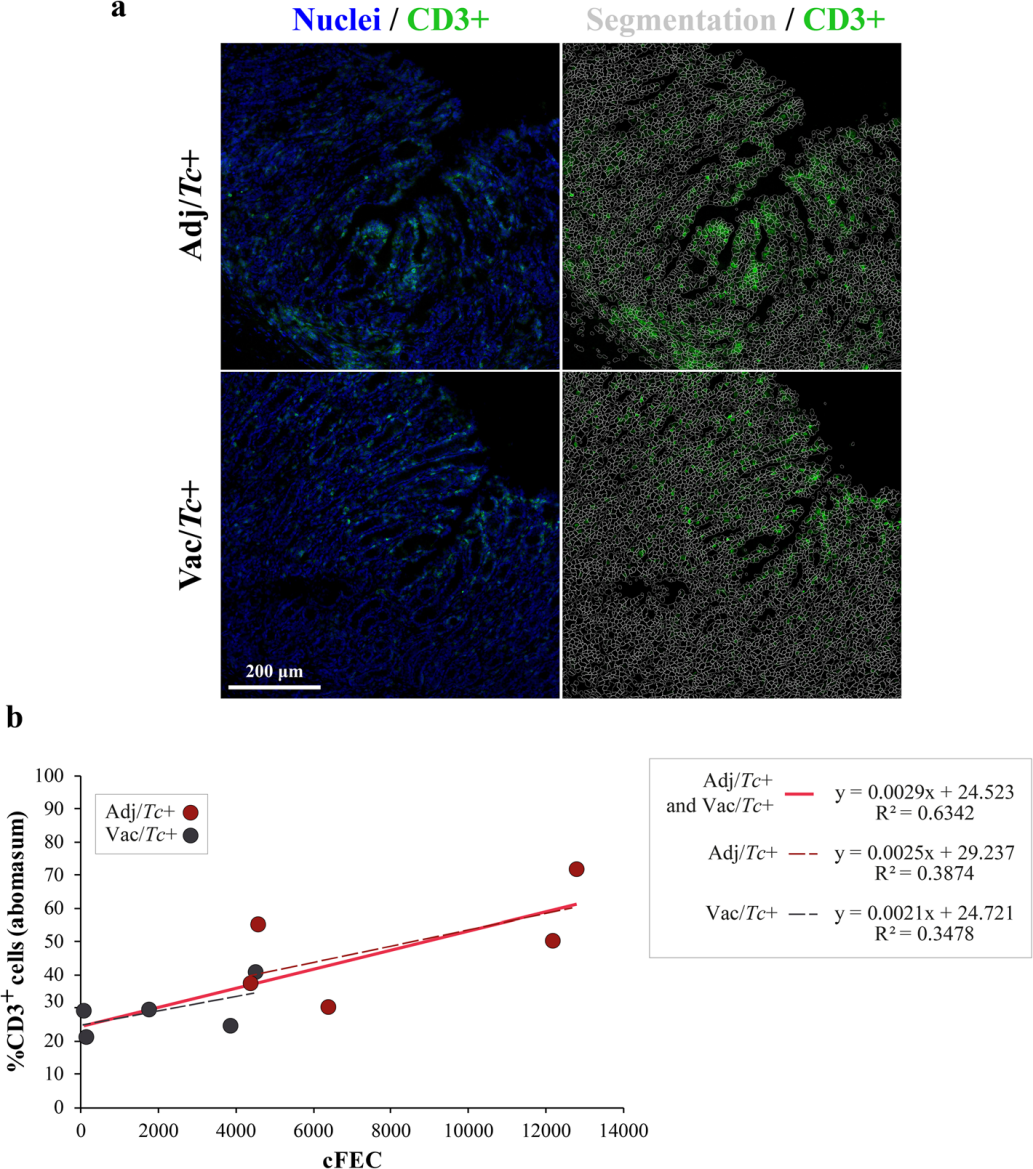
T cell populations in the abomasum correlate with cumulative faecal egg counts (cFEC). **a** Representative images (× 20) used for computational scoring of T cells (CD3^+^) in the abomasum of sheep infected with *Teladorsagia circumcincta* following adjuvant (Adj/*Tc*+) or vaccine (Vac/*Tc*+) administration. **b** Correlations between percentage of abomasal CD3^+^ cells and cFEC in infected sheep

T cells were detected both in the lamina propria and infiltrating the epithelial monolayer of the abomasa of all sheep included in the study (Fig. 4a). On average, a total of 17,671 cells were scored per sample and the percentage of CD3^+^ cells was calculated. To determine whether local recruitment of T cells was affected by anti-parasite vaccination and, in such a case, whether this effect was direct (i.e. part of the vaccine-elicited response) or indirect (i.e. resulting from a reduced worm establishment in vaccinated animals), we calculated the correlations of cFEC and worm burdens with populations of CD3^+^ cells in the abomasum. A significant correlation was observed between percentage of abomasal T cell infiltrates and cFEC detected in Adj/*Tc*+ and Vac/*Tc*+ at 57 dpi (*p* = 0.006; Pearson’s *r* = 0.796; Fig. 4b); however, no significant correlation was detected between abomasal T cell abundances and worm burdens recovered post-mortem (*p* = 0.144; Spearman’s *r* = 0.499; Additional file 1c). Linear regression of the percentage of CD3^+^ cells *versus* cFEC in each experimental group (i.e. Adj/*Tc*+ and Vac/*Tc*+, separately) showed similar slopes, indicating that the positive correlation between these two parameters, and thus, the recruitment of T cells towards the site of infection is not driven by vaccination but by infection (Fig. 4b). Spearman’s correlation applied to T lymphocyte counts and relative proportions of faecal bacterial taxa showed a positive, albeit weak, correlation between local populations of this cell type and the genus *Prevotellaceae* UCG003 (*p* = 0.049; Spearman’s *r* = 0.649; Additional file 15).

## Discussion

Determining the impact that colonisation by GI helminth parasites exerts on the composition of the gut microbial flora of their vertebrate hosts is key to the identification of populations of bacteria that may play active roles in immune and/or pathophysiological mechanisms that contribute to the outcome of infection and disease; in turn, this new knowledge could be exploited for the development of novel treatment and/or control strategies against parasitic diseases based on the manipulation of the gut microbiota. To this end, we characterised the qualitative and quantitative fluctuations in gut microbiota composition of sheep experimentally infected with a socioeconomically important GI nematode, *T*. *circumcincta*, and investigated the relative contribution of vaccine-induced host immunity to the observed changes in the proportions of populations of resident bacteria with potential roles in host-parasite interactions. Furthermore, a novel high-throughput cytometry method, based on immunofluorescence labelling coupled to computational image analysis and cell scoring [37], was set up for the study of T cell populations in the ovine abomasum, and their correlation with parasite load and the abundance of certain bacteria in the faeces of a subgroup of vaccinated and unvaccinated animals.

The overall gut microbial profiles of sheep enrolled in this study (i.e. Adj/*Tc*+, Vac/*Tc*+ and *Tc*−) was consistent with previous descriptions of the ovine gut microbiota [38, 39]. Nonetheless, experimental infection with *T*. *circumcincta* induced significant changes in the taxonomic profiles of sheep gut communities that occurred irrespective of previous vaccination. Quantitative and qualitative alterations in gut microbial populations in response to GI helminth infections have been widely reported in both humans and veterinary species (reviewed by [6, 40]) including in ruminants infected with abomasal nematodes [41–43]. Indeed, whilst our study used faecal microbial composition as a proxy of bacterial populations inhabiting other compartments of the sheep GI tract (cf. [44]; reviewed by [45]), selected alterations observed herein are consistent with previous descriptions of qualitative and quantitative changes of bacterial populations inhabiting the abomasa of ruminants infected with GI nematodes [41–43], and thus provide support to the validity of our findings. Taken together, these data confirm the major role of helminth colonisation in gut microbiome re-modelling, and that experimental vaccination prior to parasite challenge resulted in further alterations of the gut microbial profiles of Vac/*Tc*+ sheep at the end of the study period, which were likely to result from the effect of vaccination on worm survival. Indeed, vaccination alone did not result in significant changes in faecal microbial composition at EI, with the exception of samples collected at EI, for which marginally significant differences were detected by Adonis between the two sample groups, that were however not supported by CCA and RDA. Nevertheless, the absence of a vaccine-only group (a choice guided by the 3Rs principles) prevents us from speculating on the occurrence of a complex three-way network of interactions between the parasite, gut microbiome and host immune system.

No statistically significant differences in gut microbial alpha diversity were recorded over time between vaccinated/ and unvaccinated/challenged animals, nor between these and uninfected control sheep, in accordance with previous studies conducted in ruminants and other large herbivores colonised by GI helminths, e.g. goats infected with the abomasal strongyle *Haemonchus contortus* [42], partially immune cattle challenged with infective larvae of *Ostertagia ostertagi* (an abomasal nematode whose life cycle and biology closely reflect those of *T. circumcincta* in sheep) [41] and horses harbouring large-intestinal strongyles [46, 47]. On the other hand, throughout the course of the study, the gut microbiota of sheep challenged with *T*. *circumcincta* (irrespective of prior immunisation) displayed a progressively decreasing beta diversity, that reached statistical significance when the microbial profiles of samples collected prior to challenge infection were compared to that of samples collected at the end of the study period. This finding may result from the rise in pH of the gastric environment that follows the establishment of *T*. *circumcincta* [48], which is likely to enable a greater survival of selected ruminal bacteria (e.g. obligate anaerobes) transiting through the abomasum of infected ruminants [48]. In the present study, obligate anaerobes that were significantly expanded in the gut microbiota of *T*. *circumcincta* infected animals, compared to uninfected animals, included members of the *Prevotellaceae*, and *Porphyromonas* and *Sutterella*. Amongst these, *Prevotella* spp. proliferate when the pH of the medium increases [49–51]; the expansion of this genus of bacteria was also reported in the abomasum of *Haemonchus*-infected goats alongside a significant pH increase [42]. *Prevotella* spp. are key metabolisers of peptides and carbohydrates [52–55]; thus, the expansion of this genus of bacteria had been hypothesized to serve as a compensatory mechanism to counteract the protein loss caused by abomasal helminth infections [42]. Nevertheless, the significant increase of *Prevotella* observed in the faeces of parasitized animals, together with the simultaneous expansion of other bacterial taxa, e.g. *Porphyromonas* spp., is likely to contribute to the pathophysiology of *Teladorsagia* ovine infection. Notably, these two taxa are common and generally harmless members of the resident gut flora that, under particular environmental conditions, or in individuals carrying selected genetic mutations, may become pathobionts [56, 57] and cause immune-mediated diseases [58–66]. Furthermore, the expansion of the genus *Sutterella* in *Tc*+ sheep could also contribute to abomasal inflammation upon infection [67], though the mechanisms through which this taxon could enhance local inflammation are still not fully understood [68–70].

Indeed, the molecular mechanisms underlying pathobiont-mediated pathology are diverse, and often involve positive feedback loops that exacerbate inflammation (reviewed by [57]). One known mechanism involves loss of tolerance to otherwise innocuous microbes, and subsequent activation of microbiota-specific pro-inflammatory T cells (of the Th17 and Th1 subsets) in the presence of a heterologous GI infection [71, 72]. The mucosal immune responses activated upon *T*. *circumcincta* infection are complex, and involve the activation of mixed Th1/Th2/Th17 cell populations [73, 74]; in particular, a study of the transcriptome of the abomasal epithelium in response to chronic *T*. *circumcincta* infection showed that polarisation towards the Th17 subset is determinant for susceptibility to parasite colonisation (leading to gross mucosal inflammation), whilst the local immune responses of ‘resistant’ animals are polarised towards a Th2-dominated phenotype [73, 74]. In these animals, mucosal histopathology in response to infection is limited [73]. The immune-molecular interactions that determine Th cell polarisation towards the Th17 or Th2 phenotype are still unclear [75, 76]. Similarly, whether Th17-skewed responses in susceptible animals are fully addressed to the invading parasites or also to the changing microbiota is currently unknown. Indeed, our data suggest that alterations of the abomasal environment induced by *T*. *circumcincta*, including increased pH, epithelial permeability and mucosal inflammation [18], could promote the expansion of selected populations of bacteria that, in a positive loop, could trigger the onset of collateral, pathobiont-dependent inflammation. However, given that changes in the gut microbiota are likely triggered by the increase of the abomasal pH that follows worm establishment [42, 48], the mechanisms underlying differences in overall pathology between susceptible and resistant sheep (displaying Th17- and Th2-dominated immune responses, respectively) are still unclear (cf. [73, 74]).

In the present study, two animal groups with different *Teladorsagia* worm burdens, i.e. the Adj/*Tc*+ and Vac/*Tc*+ animals, were investigated. Immunisation with a cocktail of recombinant parasite antigens resulted in a 56% reduction in mean peak faecal egg output and a 30% reduction in mean cFEC (cf. [23]). Both of these measurements are key indicators of the dynamics of the increase and decrease of parasite burden during the challenge period, whereas worm burden at post mortem is indicative of the number of worms that had survived till the end of the study. Worm burden measured at post mortem following a prolonged trickle infection protocol is therefore strongly influenced by factors such as the lifespan/rate of turnover of worms and interactions with the developing, exposure-induced, natural immunity [23]. In order to gain further insights into the immune microenvironment that accompanies parasite infections and the changing abomasal microbiota in naïve and immunised animals, local populations of mucosal T cells were studied *via* quantitative tissue microscopy, removing any reliance upon subjective observations and representative images. The results of our analyses indicate that the number of T lymphocytes infiltrating the abomasal tissue is directly proportional to worm burdens. Since infiltration of T cell populations in the abomasal mucosa has been associated with tissue damage linked to developing larvae [73], the enhanced recruitment of T cells into the abomasal mucosa of Adj/*Tc*+ sheep may result from higher loads of establishing parasites in this group compared with the Vac/*Tc*+ counterpart; therefore, under the experimental conditions of this study, assessing the roles that vaccination-induced local immunity and differences in worm burdens might play in protecting the host from microbiota-dependent pathology is challenging. Indeed, the gut microbial profiles of Adj/*Tc*+ and Vac/*Tc*+ sheep were largely similar and the effect of vaccination in the current trial was relatively small; thus, whilst significant expansion of the *PeH15* family and of the *Candidatus Endomicrobium* genus of bacteria was detected in vaccinated animals, any speculation on the potential links between the abundance of these taxa and the immune features of the abomasal mucosa is currently unwarranted. However, a significant (weak) correlation was observed between numbers of T lymphocytes and the abundance of *Prevotellaceae* UCG003. This genus of bacteria was significantly expanded in both Adj/*Tc*+ and Vac/*Tc*+ sheep over the course of parasite infection; nevertheless, it was significantly more abundant in the gut microbiota of Adj/*Tc*+ than in that of Vac/*Tc*+ animals at the end of the study. The limited availability of sheep-specific antibodies for phenotypic characterisation of T lymphocytes via immunofluorescence labelling of formalin-fixed paraffin-embedded tissues impaired the identification of the specific T cell subsets whose abundance might correlate with cFEC and/or with that of selected gut microbial populations. Nonetheless, our data provide support to the hypothesis that, as for other GI helminth infections [71, 72], microbiota-specific T cell responses might be activated following colonisation of the abomasum by *T*. *circumcincta*. Thus, future studies aimed to assess the contribution of each player, i.e. the macro- and microbiota, to the immunopathology of *Teladorsagia* infection are, in our opinion, warranted and timely.

## Conclusions

Data from this study support the occurrence of significant quantitative and qualitative alterations of specific populations of gut microbes in response to *T*. *circumcincta* infection in sheep. Nevertheless, the contribution of other host- and/or helminth-dependent factors to this microbiota re-modelling cannot be currently excluded; for instance, as recently described in rodent models of infection by other species of parasitic nematodes [77], excretory/secretory products released by *T*. *circumcincta* may directly interact with the host gut microbiota and shape its composition. Our findings also suggest that selected bacterial taxa, e.g. members of the genera *Prevotella*, *Porphyromonas* and/or *Sutterella*, may contribute to the pathogenesis of PGE *via* promoting abomasal inflammation. Altogether, our data provide a solid foundation for future studies aimed at (i) identifying populations of gut microbes with key roles in the immunopathology of parasite infection and of host-, parasite- and microbiota-related web of factors that may influence disease outcome, and (ii) designing novel parasite treatment and/or control strategies based on the targeted manipulation of the host gut microbiota.

## Supplementary information

**Additional file 1.**Parasitological results for sheep subsampled for immunofluorescence, and correlation between abomasal populations of T cells and worm recoveries. Worm recoveries at post-mortem (61 days post first trickle infection) (a) and cumulative faecal egg counts (cFEC; *p<0.05) (b) in sheep infected with *Teladorsagia circumcincta* following adjuvant (Adj/*Tc*+) or vaccine (Vac/*Tc*+) administration. (c) Correlation between abomasal CD3^+^ cell populations and worm recoveries at the end of the trial.

**Additional file 2.** CellProfiler pipeline. Details of every step of all 40 modules used to perform the image analysis using the CellProfiler software.

**Additional file 3.** Parasitological results. Mean (± standard error) (a) and median (b) values of *Teladorsagia circumcincta* eggs per gram of faeces (EPG) recorded over the course of the experiment in sheep infected following adjuvant (Adj/*Tc*+; red) or vaccine (Vac/*Tc*+; black) administration. (c) Number of worms (mean ± standard error) recovered from each infected group at post-mortem (61 days post-infection).

**Additional file 4. **Rarefaction curves for faecal microbial communities. Each curve (colour) represents a different sample in the experiment, i.e. faecal DNA extracts obtained from sheep infected with *Teladorsagia circumcincta* following adjuvant (Adj/*Tc*+) or vaccine (Vac/*Tc*+) administration, and uninfected controls (*Tc*-) at indicated time points (dpi: days post first trickle infection).

**Additional file 5.** Overall faecal microbiota profiling. Krona chart displaying the most prevalent microbial domains, phyla, classes, orders and families (from inner to outer circles, respectively) for all samples included in this study.

**Additional file 6.** Differences in overall microbial composition between groups. Statistical differences in the overall microbial profiles recorded using various supervised multivariate tests (a) between infected and uninfected samples; and (b) between experimental groups at different time points.

**Additional file 7: **Microbial alpha diversity is not affected by *Teladorsagia circumcincta* infection. Boxplots representing (a) over time microbial alpha diversity calculated by Shannon index in the faeces of sheep infected with *Teladorsagia circumcincta* following adjuvant inoculation (Adj/*Tc+*) or vaccination (Vac/*Tc+*), and uninfected controls (*Tc*-). Statistical differences were assessed by Mixed Effect Linear Regression (MELR); and (b) Shannon index calculated in each experimental group at every time point. PT = pre-trial; EI = end of immunisation; dpi = days post first trickle infection; *ns* = no sample available.

**Additional file 8 **Infection is associated with a trend towards a decreased faecal microbial beta diversity. ANOSIM plots displaying the microbial beta diversity of faeces of sheep infected with *Teladorsagia circumcincta* following (a) adjuvant (Adj/*Tc*+) or (b) vaccine (Vac/*Tc*+) administration, and (c) uninfected controls (*Tc*-) over the course of the experiment. Horizontal lines and asterisks indicate statistically significant differences between indicated pairs of time points calculated by Permutational Multivariate Analysis Of Variance (PERMANOVA) (*p<0.05).

**Additional file 9. **Longitudinal changes in faecal microbiota composition. Statistically significant longitudinal changes in the abundances of faecal microbial taxa (FDR-adjusted p<0.05) in sheep infected with *Teladorsagia circumcincta* (a) following (Adj/*Tc*+) or (b) vaccine (Vac/*Tc*+) administration and (c) uninfected controls (*Tc*).

**Additional file 10. **Vaccine and/or adjuvant administration are associated with longitudinal changes in the abundance of faecal microbial taxa in infected sheep. Boxplots representing over time abundances of microbial taxa significantly altered (Mixed Effect Linear Regression FDR-adjusted q<0.05) in faecal samples from sheep infected with *Teladorsagia circumcincta* following adjuvant inoculation (Adj/*Tc+*) or vaccination (Vac/*Tc+*), and uninfected controls (*Tc*-). PT = pre-trial; EI = end of immunisation; dpi = days post first trickle infection; *ns* = no sample available; NA = not applicable.

**Additional file 11. **Infection and vaccination are associated with changes in faecal microbial profiles. Canonical Correlation Analysis (CCA) for samples collected from sheep infected with *Teladorsagia circumcincta* following adjuvant inoculation (Adj/*Tc*+) or vaccination (Vac/*Tc*+), and uninfected controls (*Tc*-) at different days post first trickle infection (dpi). Samples clustered by experimental group (a), vaccine or adjuvant administration prior to infection (b), and infection status (c). NA = Not applicable.

**Additional file 12. **Differences in faecal microbial taxa abundances between experimental groups. Microbial taxa displaying statistically significant differences in abundance between faecal samples from sheep experimentally infected with *Teladorsagia circumcincta* following adjuvant (Adj/*Tc*+) or vaccine (Vac/*Tc*+) administration and uninfected controls (*Tc*-). Results based on Linear discriminant analysis Effect Size (LEfSe); LDA score (log10) > 2.5.

**Additional file 13. **Main quantitative changes in faecal microbial composition associated with infection by *Teladorsagia circumcincta* and anti-parasite vaccination. Boxplots displaying statistically significant differences detected by DESeq2 between the indicated groups (*p<0.05; **p<0.01; ***p<0.001; ****p<0.0001). (a) Differentially abundant taxa between infected and uninfected sheep at 57 days post first trickle infection (dpi). (b) Differences in the faecal abundance of the bacterial family *PeH15* between vaccinated and unvaccinated animals over the course of the experiment. PT = pre-trial; EI = end of immunisation.

**Additional file 14. **Differences in faecal microbial taxa abundance between groups using DESeq2. Statistically significant differences in the abundance of faecal microbial taxa calculated by DESeq2 (FDR-adjusted q<0.05) between (a) infected and uninfected, and (b) vaccinated and unvaccinated animals; and (c) between infected (i.e. Adj/*Tc*+ *vs.* Vac/*Tc+*) and (D) unvaccinated (i.e. Adj/*Tc*+ *vs. Tc-*) animals at different time points.

**Additional file 15. **Correlation between bacterial populations and T lymphocytes in the abomasum. Spearman’s correlation between the abundance of *Prevotellaceae* UCG03 and the percentage of T cells in the abomasum of sheep infected with *Teladorsagia circumcincta* following adjuvant (Adj/*Tc*+) or vaccine (Vac/*Tc*+) administration at post-mortem (i.e. 57 days post first trickle infection).

## Data Availability

The 16S rRNA gene sequence datasets generated and analysed during the current study are available from Mendeley Data (DOI: 10.17632/4vgtzxr6tx.1). The complete CellProfiler image analysis pipeline alongside sample raw microscopy data are available via Biostudies under accession code S-BSST263.

## Abbreviations

ANOSIM: Analysis Of Similarity
CCA: Canonical Correlation Analysis
cFEC: Cumulative faecal egg counts
CSS: Cumulative-Sum Scaling
dpi: Days post first trickle infection
EI: End of immunisation
FDR: False Discovery Rate
FEC: Faecal egg counts
GAMM: Generalised Additive Mixed Modelling
GI: Gastrointestinal
LEfSe: Linear discriminant analysis Effect Size
MELR: Mixed Effect Linear Regression
OTU: Operational Taxonomic Unit
PCoA: Principal Coordinates Analysis
PGE: Parasite gastroenteritis
PT: Pre-trial
RDA: Redundancy Analysis
TBS: Tris-buffered saline

## References

[1] Charlier J, van der Voort M, Kenyon F, Skuce P, Vercruysse J (2014). Chasing helminths and their economic impact on farmed ruminants. Trends Parasitol..

[2] Kaplan RM, Vidyashankar AN (2012). An inconvenient truth: global worming and anthelmintic resistance. Vet Parasitol..

[3] Rose H, Rinaldi L, Bosco A, Mavrot F, de Waal T, Skuce P (2015). Widespread anthelmintic resistance in European farmed ruminants: a systematic review. Vet Rec..

[4] Moser W, Schindler C, Keiser J (2017). Efficacy of recommended drugs against soil transmitted helminths: systematic review and network meta-analysis. BMJ..

[5] Gazzinelli-Guimaraes PH, Nutman TB. Helminth parasites and immune regulation. F1000Res. 2018;7. 10.12688/f1000research.15596.1.eCollection%202018. 10.12688/f1000research.15596.1 PMC6206608 30416709

[6] Peachey LE, Jenkins TP, Cantacessi C (2017). This gut ain't big enough for both of us. Or is it? Helminth-microbiota interactions in veterinary species. Trends Parasitol..

[7] Brosschot TP, Reynolds LA (2018). The impact of a helminth-modified microbiome on host immunity. Mucosal Immunol..

[8] Glendinning L, Nausch N, Free A, Taylor DW, Mutapi F (2014). The microbiota and helminths: sharing the same niche in the human host. Parasitology..

[9] Reynolds LA, Smith KA, Filbey KJ, Harcus Y, Hewitson JP, Redpath SA (2014). Commensal-pathogen interactions in the intestinal tract: lactobacilli promote infection with, and are promoted by, helminth parasites. Gut Microbes..

[10] Fricke WF, Song Y, Wang AJ, Smith A, Grinchuk V, Mongodin E (2015). Type 2 immunity-dependent reduction of segmented filamentous bacteria in mice infected with the helminthic parasite *Nippostrongylus brasiliensis*. Microbiome..

[11] Holm JB, Sorobetea D, Kiilerich P, Ramayo-Caldas Y, Estelle J, Ma T (2015). Chronic *Trichuris muris* infection decreases diversity of the intestinal microbiota and concomitantly increases the abundance of lactobacilli. PLoS One..

[12] Zaiss MM, Rapin A, Lebon L, Dubey LK, Mosconi I, Sarter K (2015). The intestinal microbiota contributes to the ability of helminths to modulate allergic inflammation. Immunity..

[13] Ramanan D, Bowcutt R, Lee SC, Tang MS, Kurtz ZD, Ding Y (2016). Helminth infection promotes colonization resistance via type 2 immunity. Science..

[14] Jenkins TP, Rathnayaka Y, Perera PK, Peachey LE, Nolan MJ, Krause L (2017). Infections by human gastrointestinal helminths are associated with changes in faecal microbiota diversity and composition. PLoS One..

[15] Jenkins TP, Peachey LE, Ajami NJ, MacDonald AS, Hsieh MH, Brindley PJ (2018). *Schistosoma mansoni* infection is associated with quantitative and qualitative modifications of the mammalian intestinal microbiota. Sci Rep..

[16] Su C, Su L, Li Y, Long SR, Chang J, Zhang W (2018). Helminth-induced alterations of the gut microbiota exacerbate bacterial colitis. Mucosal Immunol..

[17] O'Connor LJ, Walkden-Brown SW, Kahn LP (2006). Ecology of the free-living stages of major trichostrongylid parasites of sheep. Vet Parasitol..

[18] Stear MJ, Bishop SC, Henderson NG, Scott I (2003). A key mechanism of pathogenesis in sheep infected with the nematode *Teladorsagia circumcincta*. Anim Health Res Rev..

[19] Sargison ND, MacLeay M, Morrison AA, Bartley DJ, Evans M, Chaudhry U (2019). Development of amplicon sequencing for the analysis of benzimidazole resistance allele frequencies in field populations of gastrointestinal nematodes. Int J Parasitol Drugs Drug Resist..

[20] Turnbull F, Devaney E, Morrison AA, Laing R, Bartley DJ. Genotypic characterisation of monepantel resistance in historical and newly derived field strains of *Teladorsagia circumcincta*. Int J Parasitol Drugs Drug Resist. 2019;11:59–69. 10.1016/j.ijpddr.2019.10.002 PMC6796645 31622822

[21] Nisbet AJ, McNeilly TN, Wildblood LA, Morrison AA, Bartley DJ, Bartley Y (2013). Successful immunization against a parasitic nematode by vaccination with recombinant proteins. Vaccine..

[22] Nisbet AJ, McNeilly TN, Greer AW, Bartley Y, Oliver EM, Smith S (2016). Protection of ewes against *Teladorsagia circumcincta* infection in the periparturient period by vaccination with recombinant antigens. Vet Parasitol..

[23] Nisbet AJ, McNeilly TN, Price DRG, Oliver EM, Bartley Y, Mitchell M (2019). The rational simplification of a recombinant cocktail vaccine to control the parasitic nematode *Teladorsagia circumcincta*. Int J Parasitol..

[24] Christie M, Jackson F (1982). Specific identification of strongyle eggs in small samples of sheep faeces. Res Vet Sci..

[25] Jackson E, Jackson F, Smith WD (1984). Comparison of saline incubation and pepsin digestion as methods for recovering *Ostertagia circumcincta* larvae from the abomasum of sheep. Res Vet Sci..

[26] Klindworth A, Pruesse E, Schweer T, Peplies J, Quast C, Horn M (2013). Evaluation of general 16S ribosomal RNA gene PCR primers for classical and next-generation sequencing-based diversity studies. Nucleic Acids Res..

[27] Caporaso JG, Kuczynski J, Stombaugh J, Bittinger K, Bushman FD, Costello EK (2010). QIIME allows analysis of high-throughput community sequencing data. Nat Methods..

[28] Callahan BJ, McMurdie PJ, Rosen MJ, Han AW, Johnson AJ, Holmes SP (2016). DADA2: High-resolution sample inference from Illumina amplicon data. Nat Methods..

[29] Quast C, Pruesse E, Yilmaz P, Gerken J, Schweer T, Yarza P (2013). The SILVA ribosomal RNA gene database project: improved data processing and web-based tools. Nucleic Acids Res..

[30] Zakrzewski M, Proietti C, Ellis JJ, Hasan S, Brion MJ, Berger B (2017). Calypso: a user-friendly web-server for mining and visualizing microbiome-environment interactions. Bioinformatics..

[31] Anderson MJ (2001). A new method for non-parametric multivariate analysis of variance. Austral Ecology..

[32] Clarke KR (1993). Non-parametric multivariate analyses of changes in community structure. Austral Ecology..

[33] Fitzmaurice GM, Laird NM, Ware JH. Applied longitudinal analysis. 2^nd^ ed. Wiley; 2004.

[34] Segata N, Izard J, Waldron L, Gevers D, Miropolsky L, Garrett WS (2011). Metagenomic biomarker discovery and explanation. Genome Biol..

[35] Love MI, Huber W, Anders S (2014). Moderated estimation of fold change and dispersion for RNA-seq data with DESeq2. Genome Biol..

[36] Caicedo JC, Cooper S, Heigwer F, Warchal S, Qiu P, Molnar C (2017). Data-analysis strategies for image-based cell profiling. Nat Methods..

[37] Rees P, Wills JW, Brown MR, Barnes CM, Summers HD (2019). The origin of heterogeneous nanoparticle uptake by cells. Nat Commun..

[38] Wang Y, Cao P, Wang L, Zhao Z, Chen Y, Yang Y (2017). Bacterial community diversity associated with different levels of dietary nutrition in the rumen of sheep. Appl Microbiol Biotechnol..

[39] Zhang H, Shao M, Huang H, Wang S, Ma L, Wang H (2018). The dynamic distribution of small-tail han sheep microbiota across different intestinal segments. Front Microbiol..

[40] Cortés A, Toledo R, Cantacessi C (2018). Classic models for new perspectives: delving into helminth-microbiota-immune system interactions. Trends Parasitol..

[41] Li RW, Wu S, Li W, Huang Y, Gasbarre LC (2011). Metagenome plasticity of the bovine abomasal microbiota in immune animals in response to *Ostertagia ostertagi* infection. PLoS One..

[42] Li RW, Li W, Sun J, Yu P, Baldwin RL, Urban JF (2016). The effect of helminth infection on the microbial composition and structure of the caprine abomasal microbiome. Sci Rep..

[43] El-Ashram S, Al Nasr I, Abouhajer F, El-Kemary M, Huang G, Dincel G (2017). Microbial community and ovine host response varies with early and late stages of *Haemonchus contortus* infection. Vet Res Commun..

[44] Tapio I, Shingfield KJ, McKain N, Bonin A, Fischer D, Bayat AR (2016). Oral samples as non-invasive proxies for assessing the composition of the rumen microbial community. PLoS One..

[45] Huws SA, Creevey CJ, Oyama LB, Mizrahi I, Denman SE, Popova M (2018). Addressing global ruminant agricultural challenges through understanding the rumen microbiome: past, present, and future. Front Microbiol..

[46] Clark A, Salle G, Ballan V, Reigner F, Meynadier A, Cortet J (2018). Strongyle infection and gut microbiota: profiling of resistant and susceptible horses over a grazing season. Front Physiol..

[47] Peachey LE, Molena RA, Jenkins TP, Di Cesare A, Traversa D, Hodgkinson JE (2018). The relationships between faecal egg counts and gut microbial composition in UK thoroughbreds infected by cyathostomins. Int J Parasitol..

[48] Simcock DC, Joblin KN, Scott I, Burgess DM, Rogers CW, Pomroy WE (1999). Hypergastrinaemia, abomasal bacterial population densities and pH in sheep infected with *Ostertagia circumcincta*. Int J Parasitol..

[49] Fernando SC, Purvis HT, Najar FZ, Sukharnikov LO, Krehbiel CR, Nagaraja TG (2010). Rumen microbial population dynamics during adaptation to a high-grain diet. Appl Environ Microbiol..

[50] De Nardi R, Marchesini G, Li S, Khafipour E, Plaizier KJ, Gianesella M (2016). Metagenomic analysis of rumen microbial population in dairy heifers fed a high grain diet supplemented with dicarboxylic acids or polyphenols. BMC Vet Res..

[51] Kim YH, Nagata R, Ohkubo A, Ohtani N, Kushibiki S, Ichijo T (2018). Changes in ruminal and reticular pH and bacterial communities in Holstein cattle fed a high-grain diet. BMC Vet Res..

[52] Walker ND, McEwan NR, Wallace RJ (2005). A pepD-like peptidase from the ruminal bacterium, *Prevotella albensis*. FEMS Microbiol Lett.

[53] Matsui H, Ogata K, Tajima K, Nakamura M, Nagamine T, Aminov RI (2000). Phenotypic characterization of polysaccharidases produced by four *Prevotella* type strains. Curr Microbiol..

[54] Mao S, Zhang M, Liu J, Zhu W (2015). Characterising the bacterial microbiota across the gastrointestinal tracts of dairy cattle: membership and potential function. Sci Rep..

[55] Wirth R, Kadar G, Kakuk B, Maroti G, Bagi Z, Szilagyi A (2018). The planktonic core microbiome and core functions in the cattle rumen by next generation sequencing. Front Microbiol..

[56] Chow J, Tang H, Mazmanian SK (2011). Pathobionts of the gastrointestinal microbiota and inflammatory disease. Curr Opin Immunol..

[57] Zechner EL (2017). Inflammatory disease caused by intestinal pathobionts. Curr Opin Microbiol..

[58] Heimesaat MM, Bereswill S, Fischer A, Fuchs D, Struck D, Niebergall J (2006). Gram-negative bacteria aggravate murine small intestinal Th1-type immunopathology following oral infection with *Toxoplasma gondii*. J Immunol..

[59] Lucke K, Miehlke S, Jacobs E, Schuppler M (2006). Prevalence of *Bacteroides* and *Prevotella* spp. in ulcerative colitis. J Med Microbiol..

[60] Elinav E, Strowig T, Kau AL, Henao-Mejia J, Thaiss CA, Booth CJ (2011). NLRP6 inflammasome regulates colonic microbial ecology and risk for colitis. Cell..

[61] Scher JU, Sczesnak A, Longman RS, Segata N, Ubeda C, Bielski C (2013). Expansion of intestinal *Prevotella copri* correlates with enhanced susceptibility to arthritis. Elife..

[62] Arimatsu K, Yamada H, Miyazawa H, Minagawa T, Nakajima M, Ryder MI (2014). Oral pathobiont induces systemic inflammation and metabolic changes associated with alteration of gut microbiota. Sci Rep..

[63] Dillon SM, Lee EJ, Kotter CV, Austin GL, Gianella S, Siewe B (2016). Gut dendritic cell activation links an altered colonic microbiome to mucosal and systemic T-cell activation in untreated HIV-1 infection. Mucosal Immunol..

[64] Nakajima M, Arimatsu K, Kato T, Matsuda Y, Minagawa T, Takahashi N (2015). Oral administration of *P. gingivalis* induces dysbiosis of gut microbiota and impaired barrier function leading to dissemination of enterobacteria to the liver. PLoS One.

[65] Maeda Y, Kurakawa T, Umemoto E, Motooka D, Ito Y, Gotoh K (2016). Dysbiosis contributes to arthritis development via activation of autoreactive t cells in the intestine. Arthritis Rheumatol..

[66] Sato K, Takahashi N, Kato T, Matsuda Y, Yokoji M, Yamada M (2017). Aggravation of collagen-induced arthritis by orally administered *Porphyromonas gingivalis* through modulation of the gut microbiota and gut immune system. Sci Rep..

[67] Menon R, Ramanan V, Korolev KS (2018). Interactions between species introduce spurious associations in microbiome studies. PLoS Comput Biol..

[68] Mukhopadhya I, Hansen R, Nicholl CE, Alhaidan YA, Thomson JM, Berry SH (2011). A comprehensive evaluation of colonic mucosal isolates of *Sutterella wadsworthensis* from inflammatory bowel disease. PLoS One..

[69] Moon C, Baldridge MT, Wallace MA, DCA B, Virgin HW (2015). Vertically transmitted faecal IgA levels determine extra-chromosomal phenotypic variation. Nature.

[70] Hiippala K, Kainulainen V, Kalliomaki M, Arkkila P, Satokari R (2016). Mucosal prevalence and interactions with the epithelium indicate commensalism of *Sutterella* spp. Front Microbiol..

[71] Hand TW, Dos Santos LM, Bouladoux N, Molloy MJ, Pagan AJ, Pepper M (2012). Acute gastrointestinal infection induces long-lived microbiota-specific T cell responses. Science..

[72] Xu M, Pokrovskii M, Ding Y, Yi R, Au C, Harrison OJ (2018). c-MAF-dependent regulatory T cells mediate immunological tolerance to a gut pathobiont. Nature..

[73] Gossner AG, Venturina VM, Shaw DJ, Pemberton JM, Hopkins J (2012). Relationship between susceptibility of Blackface sheep to *Teladorsagia circumcincta* infection and an inflammatory mucosal T cell response. Vet Res..

[74] Gossner A, Wilkie H, Joshi A, Hopkins J (2013). Exploring the abomasal lymph node transcriptome for genes associated with resistance to the sheep nematode *Teladorsagia circumcincta*. Vet Res..

[75] Wilkie H, Gossner A, Bishop S, Hopkins J (2016). Variations in T cell transcription factor sequence and expression associated with resistance to the sheep nematode *Teladorsagia circumcincta*. PLoS One.

[76] Wilkie H, Nicol L, Gossner A, Hopkins J (2016). Mucosal expression of T cell gene variants is associated with differential resistance to *Teladorsagia circumcincta*. PLoS One..

[77] Rausch S, Midha A, Kuhring M, Affinass N, Radonic A, Kuhl AA (2018). Parasitic nematodes exert antimicrobial activity and benefit from microbiota-driven support for host immune regulation. Front Immunol..

