# Supplementary figures and images for: Infection with the sheep gastrointestinal nematode Teladorsagia circumcincta increases luminal pathobionts
Source: Microbiome. 2020 Apr 30;8:60. doi: 10.1186/s40168-020-00818-9 (PMC7193420; doi:10.1186/s40168-020-00818-9)

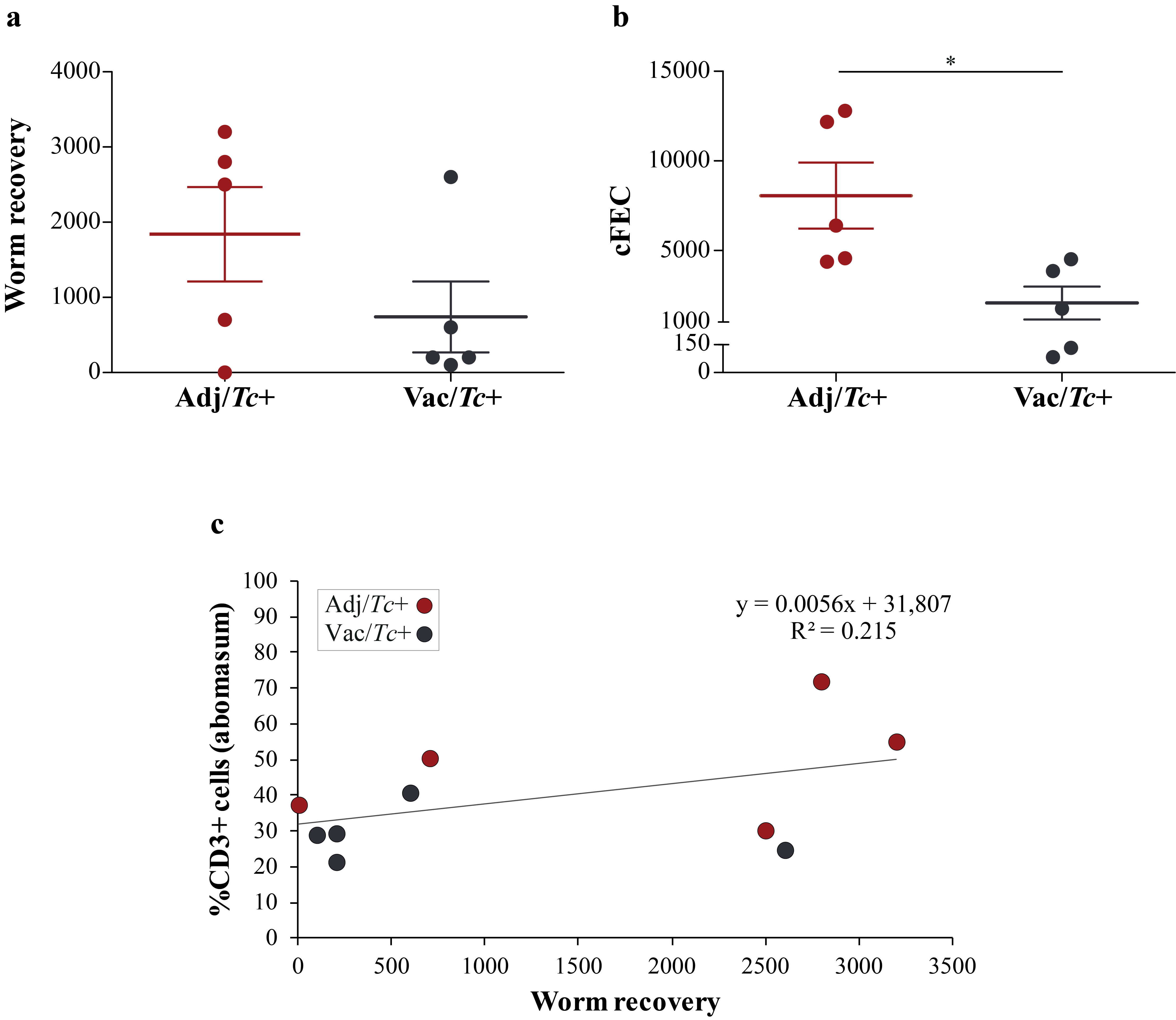

Supplement: Supplementary file 2 — Additional file 1.Parasitological results for sheep subsampled for immunofluorescence, and correlation between abomasal populations of T cells and worm recoveries. Worm recoveries at post-mortem (61 days post first trickle infection) (a) and cumulative faecal egg counts (cFEC; *p<0.05) (b) in sheep infected with Teladorsagia circumcincta following adjuvant (Adj/Tc+) or vaccine (Vac/Tc+) administration. (c) Correlation between abomasal CD3+ cell populations and worm recoveries at the end of the trial. [file 40168_2020_818_MOESM1_ESM.png]

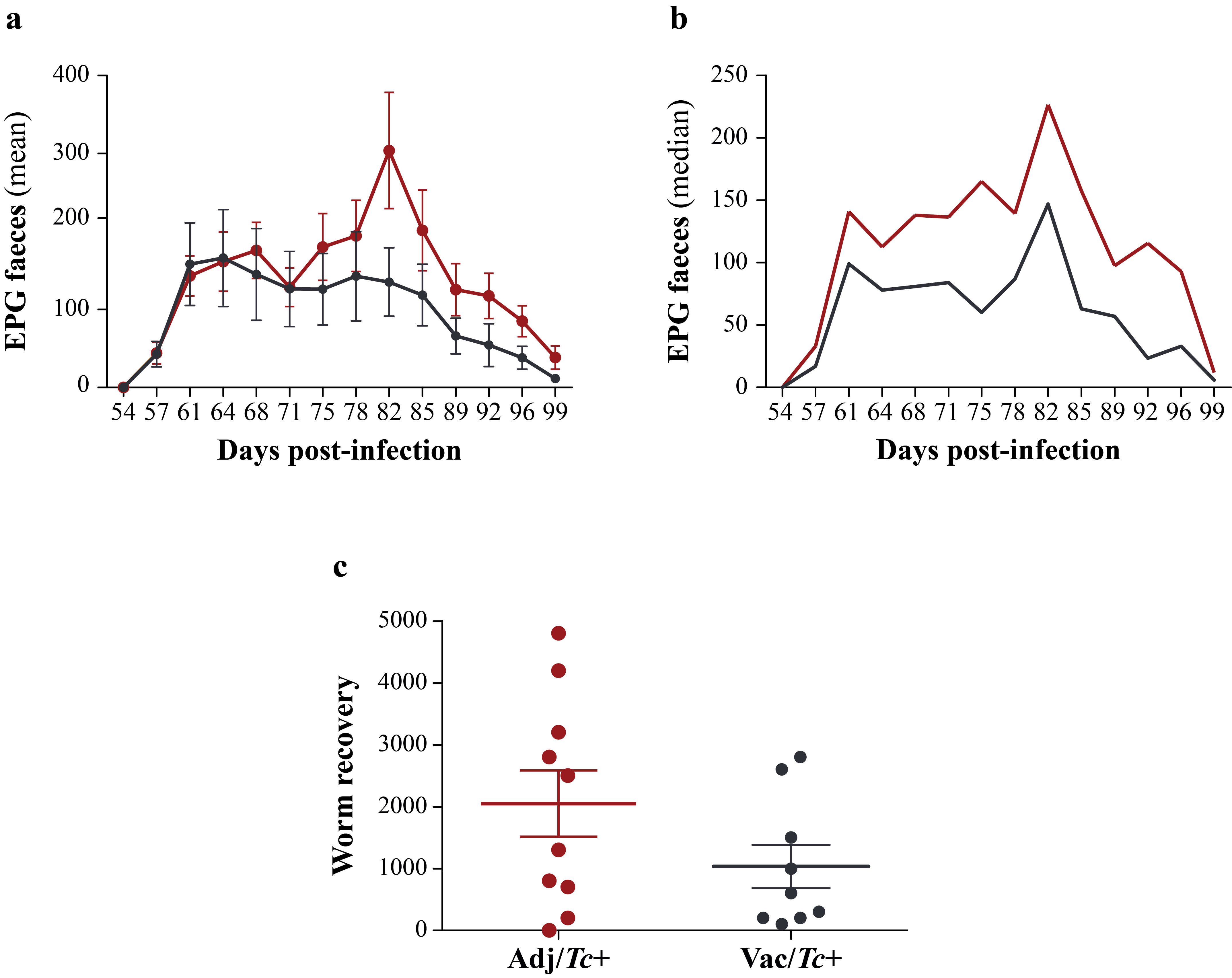

Supplement: Supplementary file 4 — Additional file 3. Parasitological results. Mean (± standard error) (a) and median (b) values of Teladorsagia circumcincta eggs per gram of faeces (EPG) recorded over the course of the experiment in sheep infected following adjuvant (Adj/Tc+; red) or vaccine (Vac/Tc+; black) administration. (c) Number of worms (mean ± standard error) recovered from each infected group at post-mortem (61 days post-infection). [file 40168_2020_818_MOESM3_ESM.png]

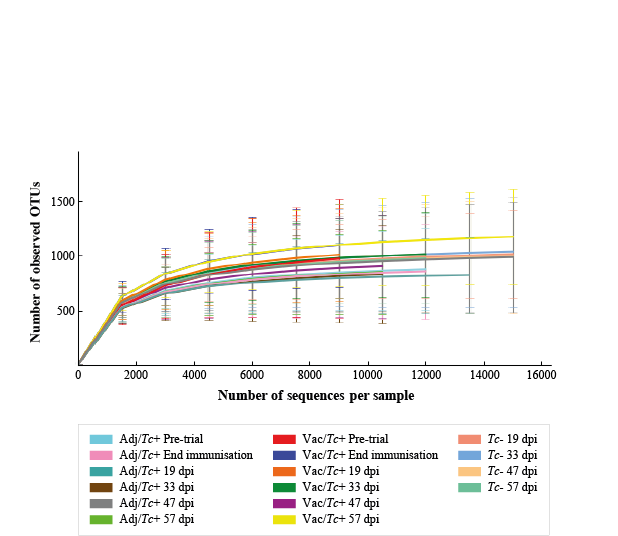

Supplement: Supplementary file 5 — Additional file 4. Rarefaction curves for faecal microbial communities. Each curve (colour) represents a different sample in the experiment, i.e. faecal DNA extracts obtained from sheep infected with Teladorsagia circumcincta following adjuvant (Adj/Tc+) or vaccine (Vac/Tc+) administration, and uninfected controls (Tc-) at indicated time points (dpi: days post first trickle infection). [file 40168_2020_818_MOESM4_ESM.png]

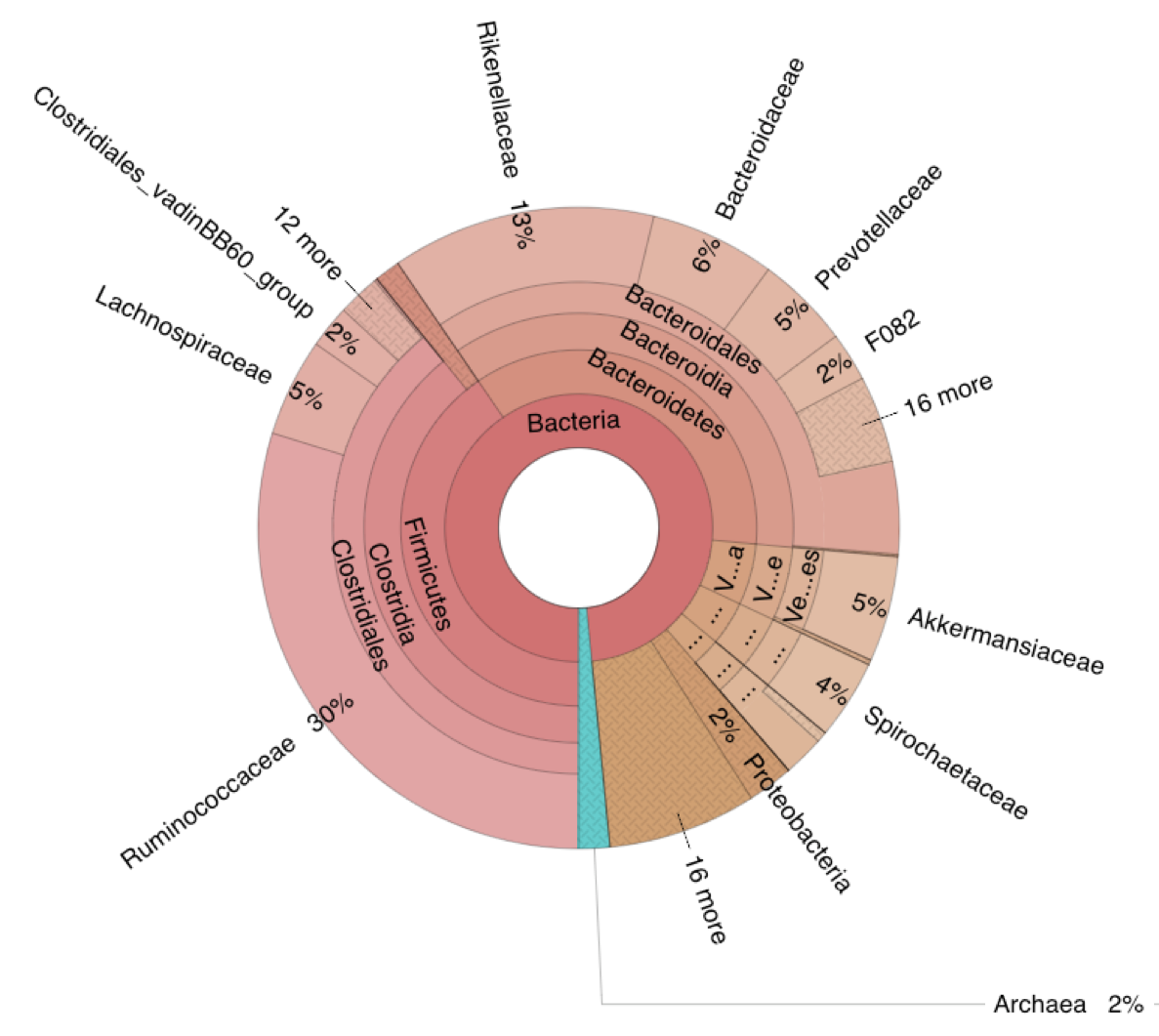

Supplement: Supplementary file 6 — Additional file 5. Overall faecal microbiota profiling. Krona chart displaying the most prevalent microbial domains, phyla, classes, orders and families (from inner to outer circles, respectively) for all samples included in this study. [file 40168_2020_818_MOESM5_ESM.png]

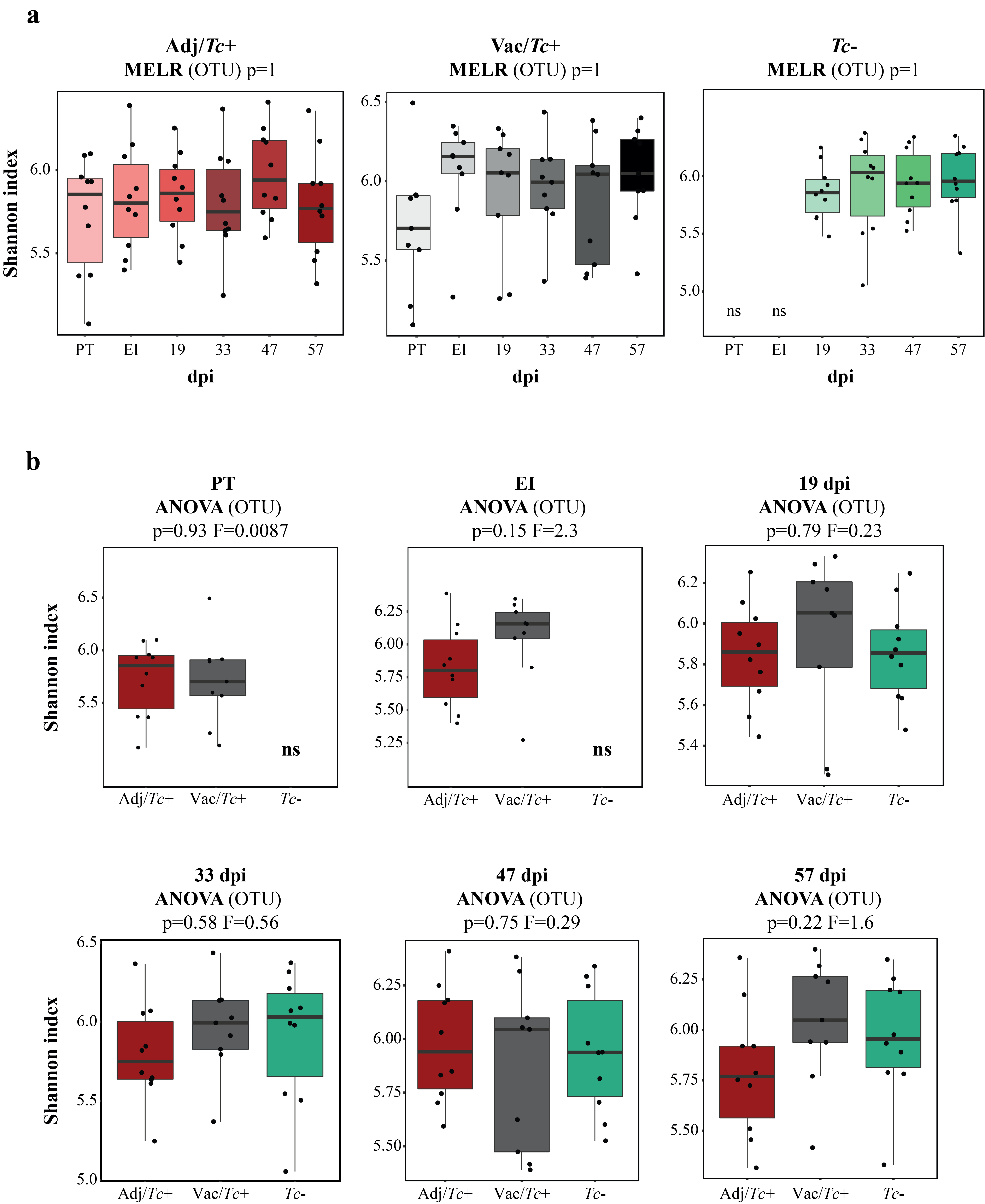

Supplement: Supplementary file 8 — Additional file 7: Microbial alpha diversity is not affected by Teladorsagia circumcincta infection. Boxplots representing (a) over time microbial alpha diversity calculated by Shannon index in the faeces of sheep infected with Teladorsagia circumcincta following adjuvant inoculation (Adj/Tc+) or vaccination (Vac/Tc+), and uninfected controls (Tc-). Statistical differences were assessed by Mixed Effect Linear Regression (MELR); and (b) Shannon index calculated in each experimental group at every time point. PT = pre-trial; EI = end of immunisation; dpi = days post first trickle infection; ns = no sample available. [file 40168_2020_818_MOESM7_ESM.png]

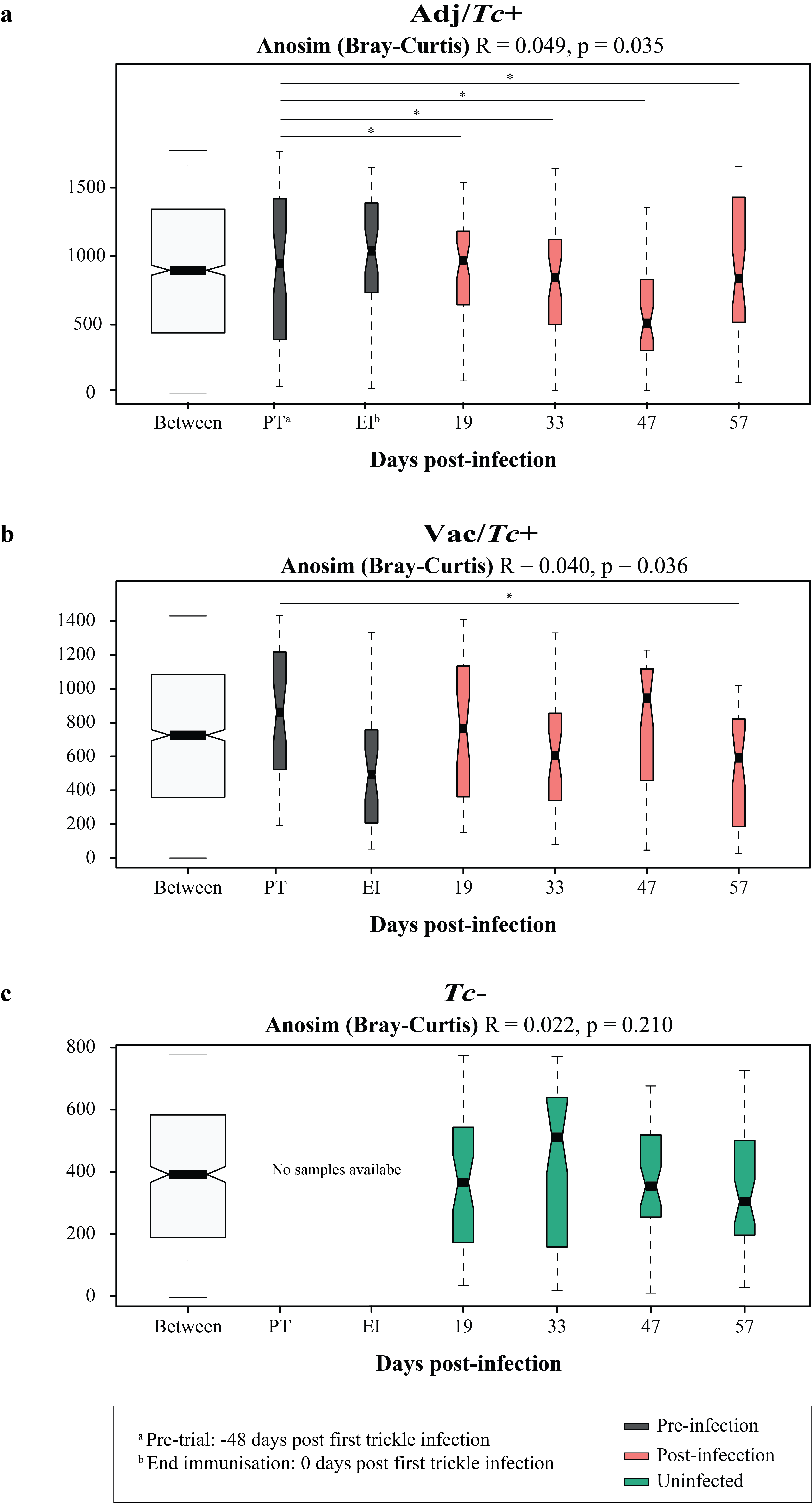

Supplement: Supplementary file 9 — Additional file 8 Infection is associated with a trend towards a decreased faecal microbial beta diversity. ANOSIM plots displaying the microbial beta diversity of faeces of sheep infected with Teladorsagia circumcincta following (a) adjuvant (Adj/Tc+) or (b) vaccine (Vac/Tc+) administration, and (c) uninfected controls (Tc-) over the course of the experiment. Horizontal lines and asterisks indicate statistically significant differences between indicated pairs of time points calculated by Permutational Multivariate Analysis Of Variance (PERMANOVA) (*p<0.05). [file 40168_2020_818_MOESM8_ESM.png]

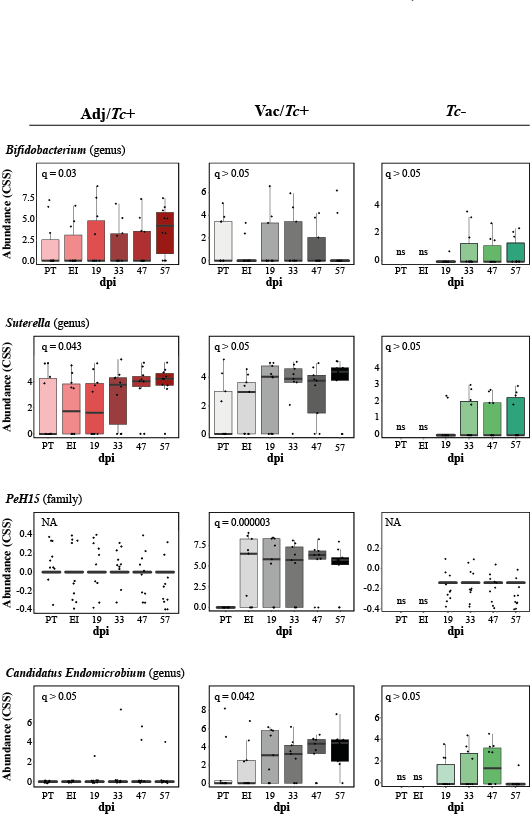

Supplement: Supplementary file 11 — Additional file 10. Vaccine and/or adjuvant administration are associated with longitudinal changes in the abundance of faecal microbial taxa in infected sheep. Boxplots representing over time abundances of microbial taxa significantly altered (Mixed Effect Linear Regression FDR-adjusted q<0.05) in faecal samples from sheep infected with Teladorsagia circumcincta following adjuvant inoculation (Adj/Tc+) or vaccination (Vac/Tc+), and uninfected controls (Tc-). PT = pre-trial; EI = end of immunisation; dpi = days post first trickle infection; ns = no sample available; NA = not applicable. [file 40168_2020_818_MOESM10_ESM.png]

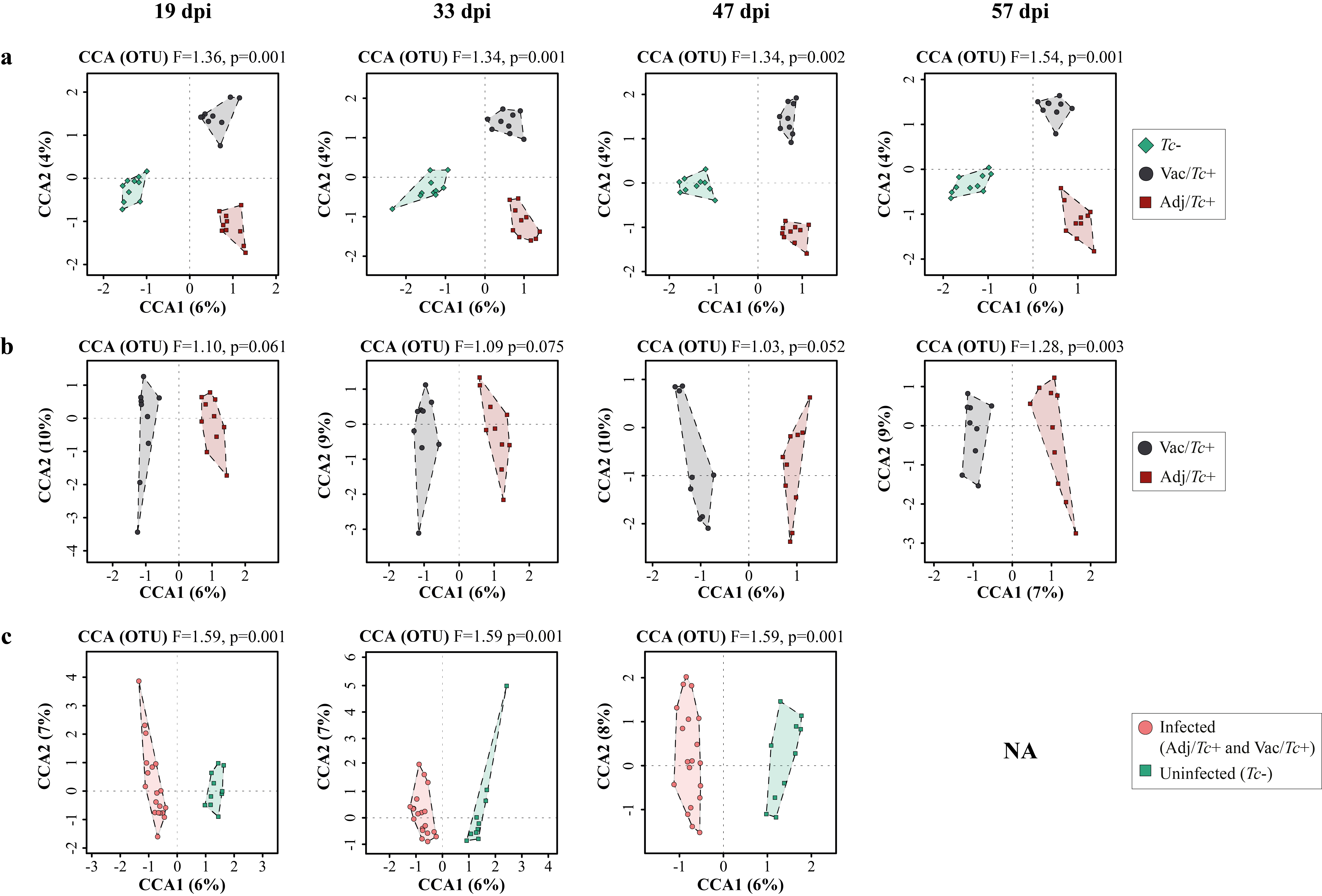

Supplement: Supplementary file 12 — Additional file 11. Infection and vaccination are associated with changes in faecal microbial profiles. Canonical Correlation Analysis (CCA) for samples collected from sheep infected with Teladorsagia circumcincta following adjuvant inoculation (Adj/Tc+) or vaccination (Vac/Tc+), and uninfected controls (Tc-) at different days post first trickle infection (dpi). Samples clustered by experimental group (a), vaccine or adjuvant administration prior to infection (b), and infection status (c). NA = Not applicable. [file 40168_2020_818_MOESM11_ESM.png]

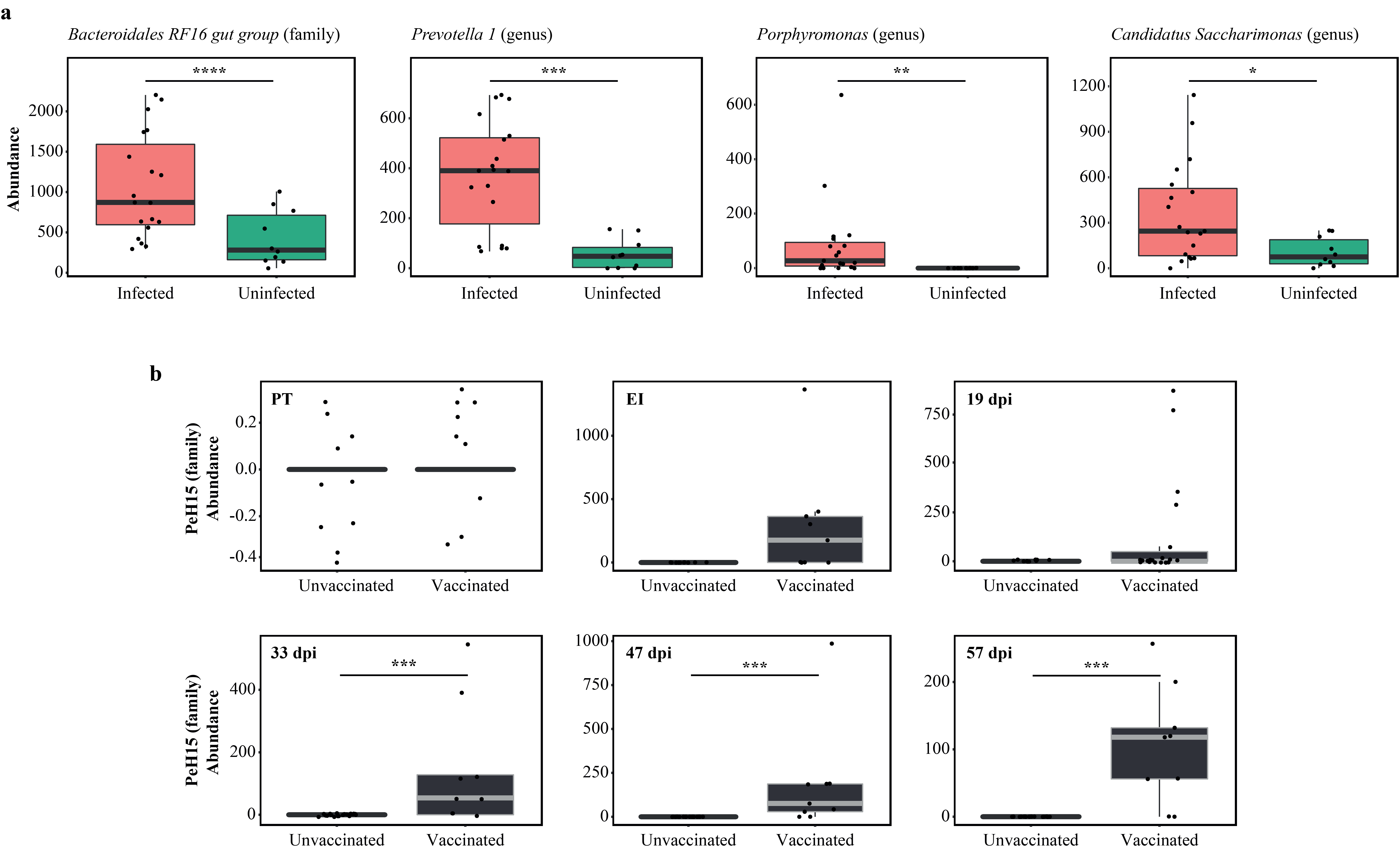

Supplement: Supplementary file 14 — Additional file 13. Main quantitative changes in faecal microbial composition associated with infection by Teladorsagia circumcincta and anti-parasite vaccination. Boxplots displaying statistically significant differences detected by DESeq2 between the indicated groups (*p<0.05; **p<0.01; ***p<0.001; ****p<0.0001). (a) Differentially abundant taxa between infected and uninfected sheep at 57 days post first trickle infection (dpi). (b) Differences in the faecal abundance of the bacterial family PeH15 between vaccinated and unvaccinated animals over the course of the experiment. PT = pre-trial; EI = end of immunisation. [file 40168_2020_818_MOESM13_ESM.png]

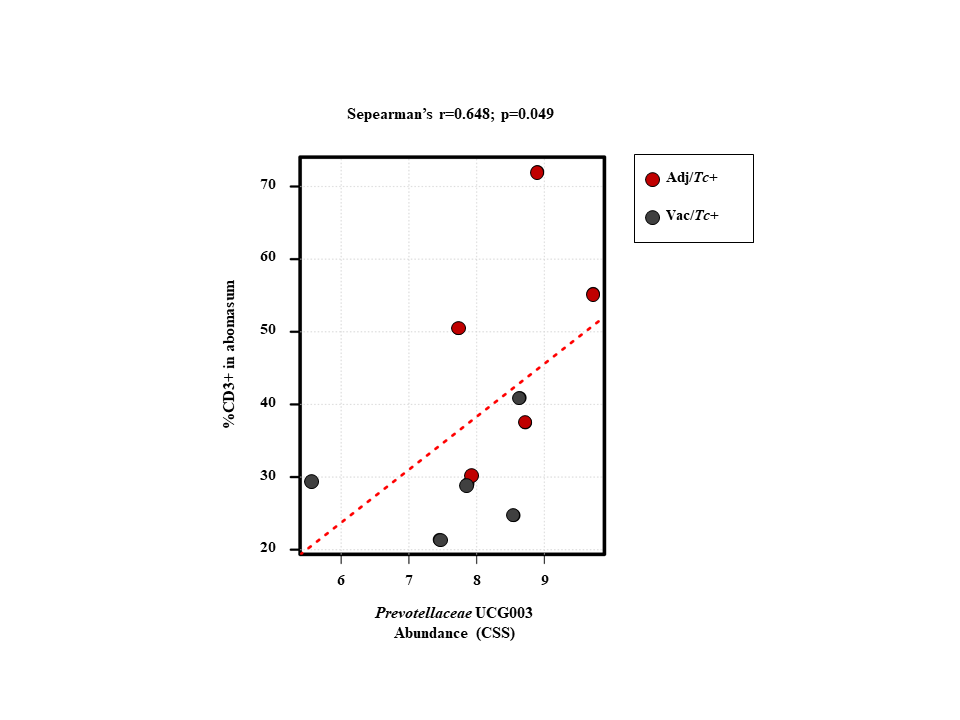

Supplement: Supplementary file 16 — Additional file 15. Correlation between bacterial populations and T lymphocytes in the abomasum. Spearman’s correlation between the abundance of Prevotellaceae UCG03 and the percentage of T cells in the abomasum of sheep infected with Teladorsagia circumcincta following adjuvant (Adj/Tc+) or vaccine (Vac/Tc+) administration at post-mortem (i.e. 57 days post first trickle infection). [file 40168_2020_818_MOESM15_ESM.png]
