# Supplementary material for: Infection with the sheep gastrointestinal nematode Teladorsagia circumcincta increases luminal pathobionts
Source: Microbiome. 2020 Apr 30;8:60. doi: 10.1186/s40168-020-00818-9 (PMC7193420; doi:10.1186/s40168-020-00818-9)
Supplement: Supplementary file 3 — Additional file 2. CellProfiler pipeline. Details of every step of all 40 modules used to perform the image analysis using the CellProfiler software. [file 40168_2020_818_MOESM2_ESM.pdf]

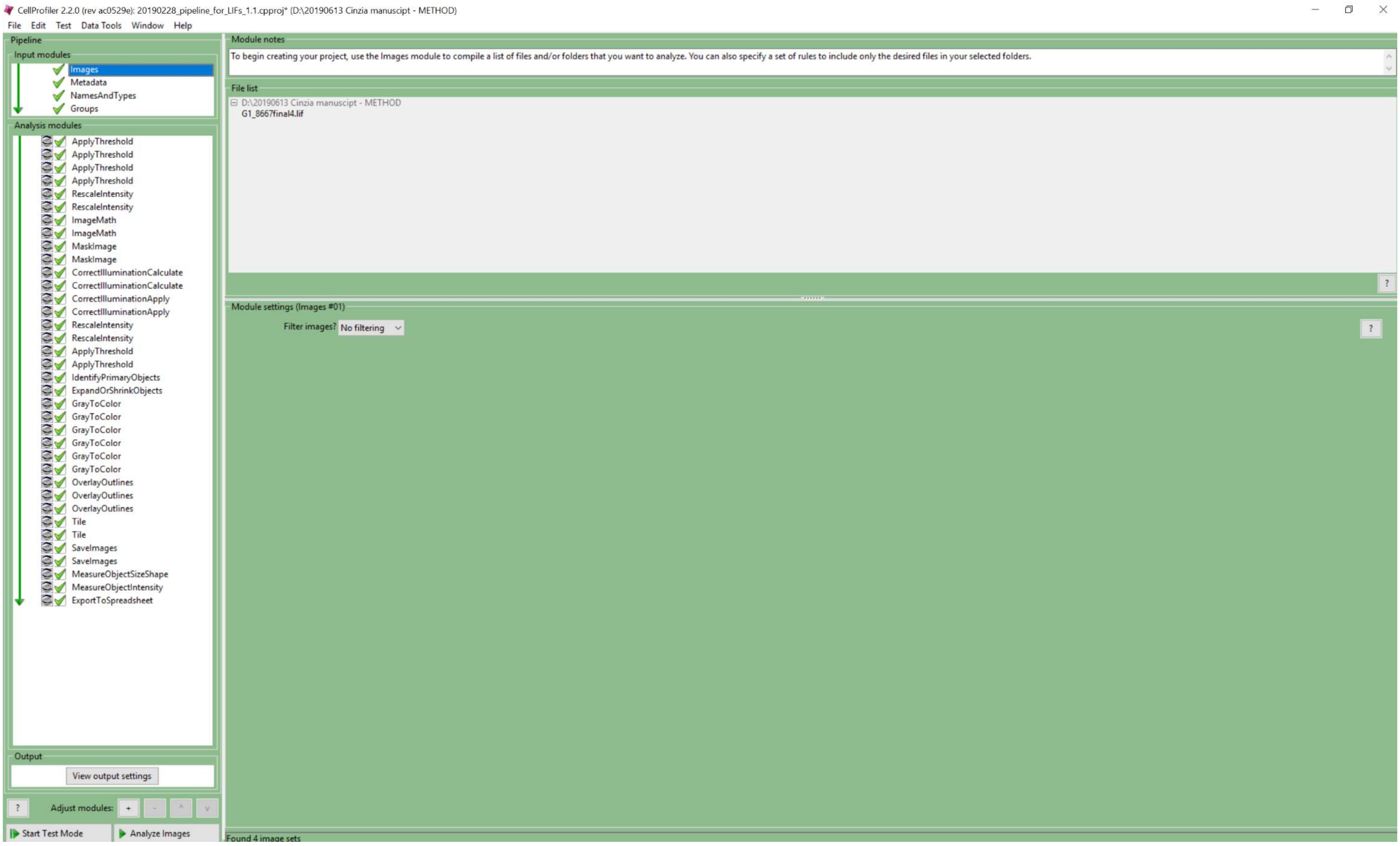
CellProfiler 2.2.0 (rev ac0529e): 20190228_pipeline_for_LIFs_1.1.cpproj* (D:\20190613 Cinzia manuscipt - METHOD)
File Edit Test Data Tools Window Help
Pipeline
Input modules
Images
Metadata
NamesAndTypes
Groups
Analysis modules
ApplyThreshold
ApplyThreshold
ApplyThreshold
ApplyThreshold
RescaleIntensity
RescaleIntensity
ImageMath
ImageMath
MaskImage
MaskImage
CorrectIlluminationCalculate
CorrectIlluminationCalculate
CorrectIlluminationApply
CorrectIlluminationApply
RescaleIntensity
RescaleIntensity
ApplyThreshold
ApplyThreshold
IdentifyPrimaryObjects
ExpandOrShrinkObjects
GrayToColor
GrayToColor
GrayToColor
GrayToColor
GrayToColor
GrayToColor
OverlayOutlines
OverlayOutlines
OverlayOutlines
Tile
Tile
SaveImages
SaveImages
MeasureObjectSizeShape
MeasureObjectIntensity
ExportToSpreadsheet
Output
View output settings
Adjust modules:
Start Test Mode
Analyze Images
Module notes
To begin creating your project, use the Images module to compile a list of files and/or folders that you want to analyze. You can also specify a set of rules to include only the desired files in your selected folders.
File list
D:\20190613 Cinzia manuscipt - METHOD
G1_8667final4.lif
Module settings (Images #01)
Filter images? No filtering
Found 4 image sets

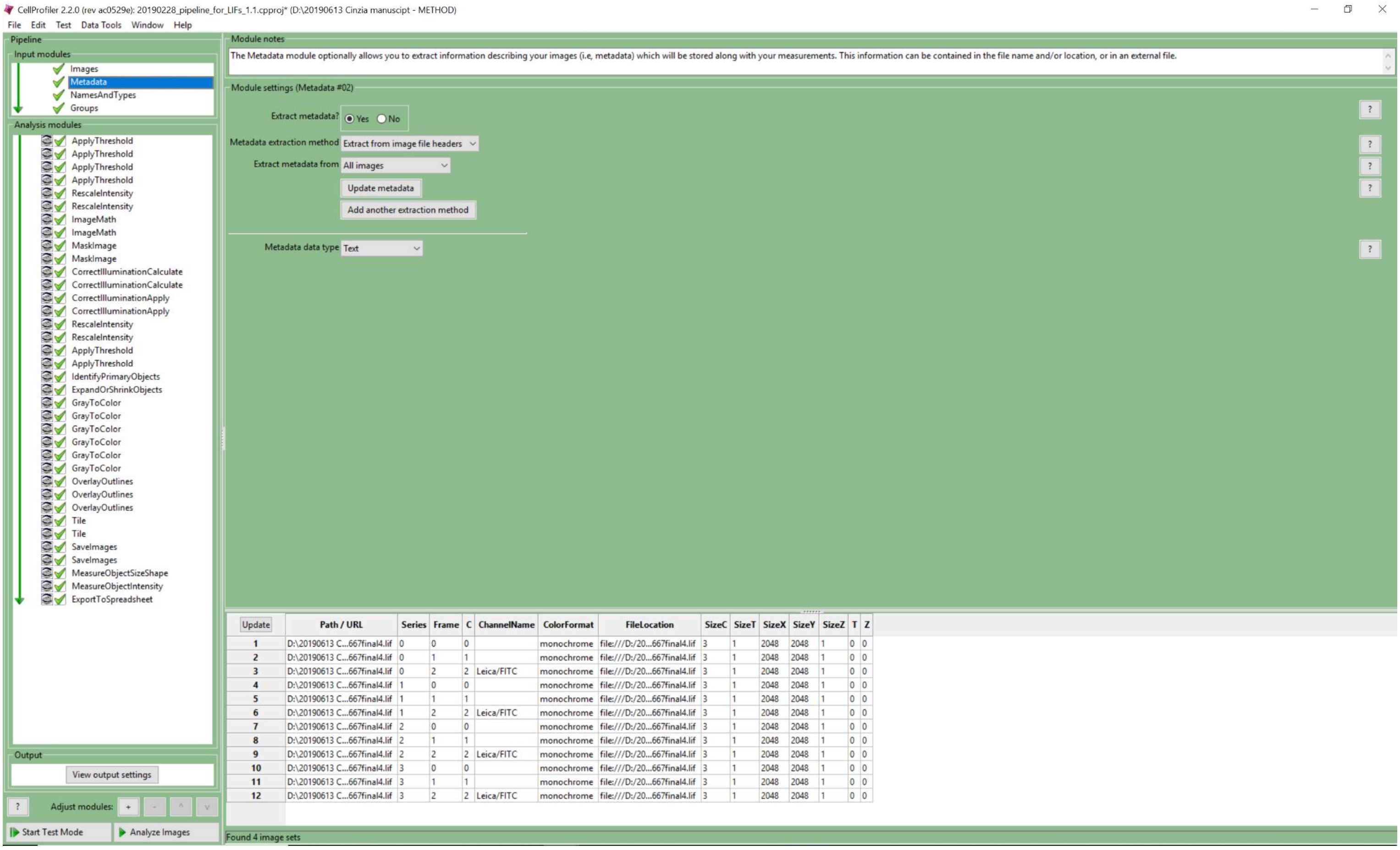
CellProfiler 2.2.0 (rev ac0529e): 20190228_pipeline_for_LIFs_1.1.cpproj* (D:\20190613 Cinzia manuscipt - METHOD)
File Edit Test Data Tools Window Help
Pipeline
Input modules
Images
Metadata
NamesAndTypes
Groups
Analysis modules
ApplyThreshold
ApplyThreshold
ApplyThreshold
ApplyThreshold
RescaleIntensity
RescaleIntensity
ImageMath
ImageMath
MaskImage
MaskImage
CorrectIlluminationCalculate
CorrectIlluminationCalculate
CorrectIlluminationApply
CorrectIlluminationApply
RescaleIntensity
RescaleIntensity
ApplyThreshold
ApplyThreshold
IdentifyPrimaryObjects
ExpandOrShrinkObjects
GrayToColor
GrayToColor
GrayToColor
GrayToColor
GrayToColor
GrayToColor
OverlayOutlines
OverlayOutlines
OverlayOutlines
Tile
Tile
SaveImages
SaveImages
MeasureObjectSizeShape
MeasureObjectIntensity
ExportToSpreadsheet
Output
View output settings
Adjust modules:
Start Test Mode
Analyze Images
Module notes
The Metadata module optionally allows you to extract information describing your images (i.e, metadata) which will be stored along with your measurements. This information can be contained in the file name and/or location, or in an external file.
Module settings (Metadata #02)
Extract metadata? Yes No
Metadata extraction method Extract from image file headers
Extract metadata from All images
Update metadata
Add another extraction method
Metadata data type Text
Update Path / URL Series Frame C ChannelName ColorFormat FileLocation SizeC SizeT SizeX SizeY SizeZ T Z
1 D:\20190613 C...667final4.lif 0 0 0 monochrome file:///D:/20...667final4.lif 3 1 2048 2048 1 0 0
2 D:\20190613 C...667final4.lif 0 1 1 monochrome file:///D:/20...667final4.lif 3 1 2048 2048 1 0 0
3 D:\20190613 C...667final4.lif 0 2 2 Leica/FITC monochrome file:///D:/20...667final4.lif 3 1 2048 2048 1 0 0
4 D:\20190613 C...667final4.lif 1 0 0 monochrome file:///D:/20...667final4.lif 3 1 2048 2048 1 0 0
5 D:\20190613 C...667final4.lif 1 1 1 monochrome file:///D:/20...667final4.lif 3 1 2048 2048 1 0 0
6 D:\20190613 C...667final4.lif 1 2 2 Leica/FITC monochrome file:///D:/20...667final4.lif 3 1 2048 2048 1 0 0
7 D:\20190613 C...667final4.lif 2 0 0 monochrome file:///D:/20...667final4.lif 3 1 2048 2048 1 0 0
8 D:\20190613 C...667final4.lif 2 1 1 monochrome file:///D:/20...667final4.lif 3 1 2048 2048 1 0 0
9 D:\20190613 C...667final4.lif 2 2 2 Leica/FITC monochrome file:///D:/20...667final4.lif 3 1 2048 2048 1 0 0
10 D:\20190613 C...667final4.lif 3 0 0 monochrome file:///D:/20...667final4.lif 3 1 2048 2048 1 0 0
11 D:\20190613 C...667final4.lif 3 1 1 monochrome file:///D:/20...667final4.lif 3 1 2048 2048 1 0 0
12 D:\20190613 C...667final4.lif 3 2 2 Leica/FITC monochrome file:///D:/20...667final4.lif 3 1 2048 2048 1 0 0
Found 4 image sets

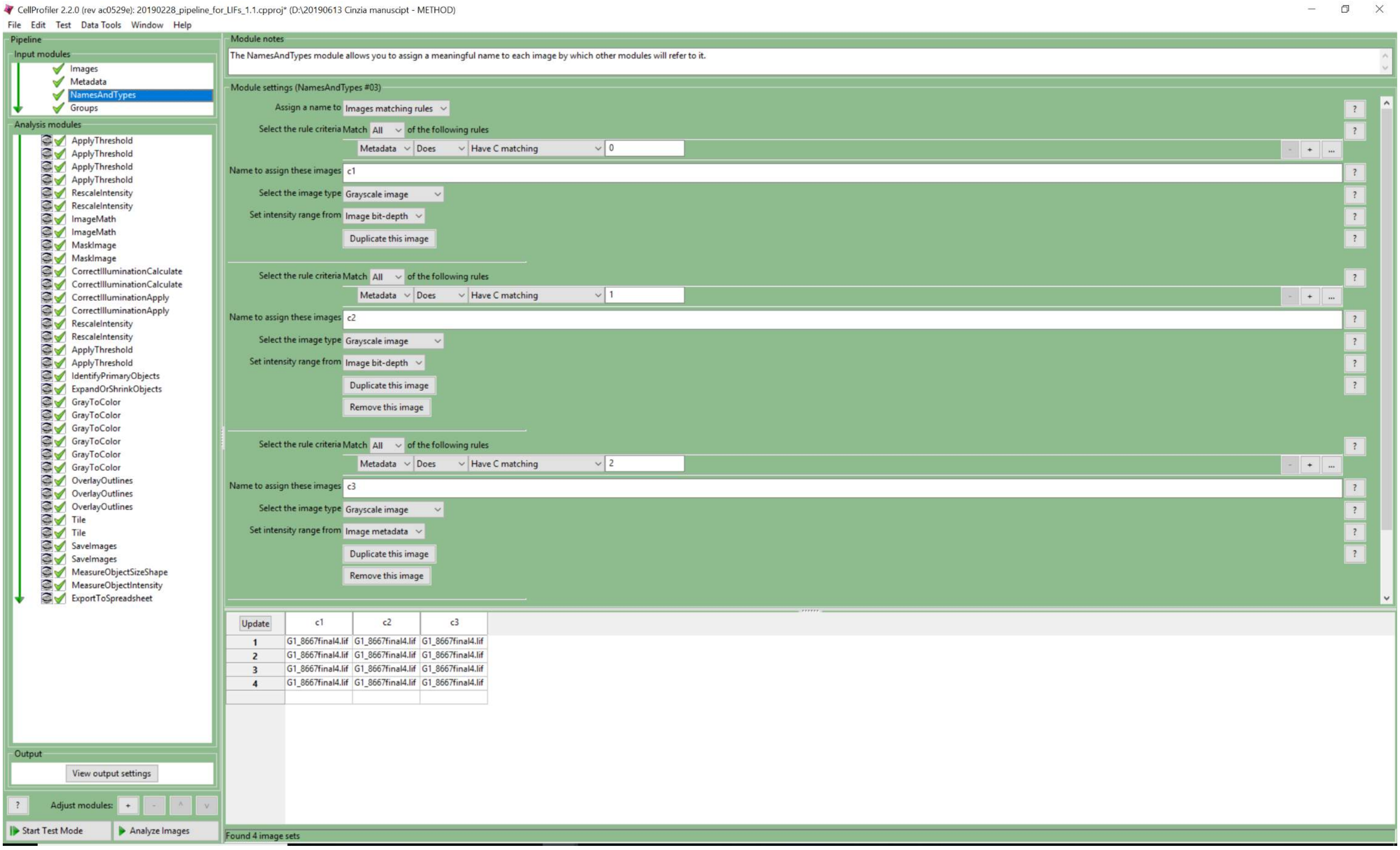
CellProfiler 2.2.0 (rev ac0529e): 20190228_pipeline_for_LIFs_1.1.cpproj* (D:\20190613 Cinzia manuscipt - METHOD)
File Edit Test Data Tools Window Help
Pipeline
Input modules
Images
Metadata
NamesAndTypes
Groups
Analysis modules
ApplyThreshold
ApplyThreshold
ApplyThreshold
ApplyThreshold
RescaleIntensity
RescaleIntensity
ImageMath
ImageMath
MaskImage
MaskImage
CorrectIlluminationCalculate
CorrectIlluminationCalculate
CorrectIlluminationApply
CorrectIlluminationApply
RescaleIntensity
RescaleIntensity
ApplyThreshold
ApplyThreshold
IdentifyPrimaryObjects
ExpandOrShrinkObjects
GrayToColor
GrayToColor
GrayToColor
GrayToColor
GrayToColor
GrayToColor
OverlayOutlines
OverlayOutlines
OverlayOutlines
Tile
Tile
SaveImages
SaveImages
MeasureObjectSizeShape
MeasureObjectIntensity
ExportToSpreadsheet
Output
View output settings
Adjust modules:
Start Test Mode
Analyze Images
Module notes
The NamesAndTypes module allows you to assign a meaningful name to each image by which other modules will refer to it.
Module settings (NamesAndTypes #03)
Assign a name to Images matching rules
Select the rule criteria Match All of the following rules
Metadata Does Have C matching 0
Name to assign these images c1
Select the image type Grayscale image
Set intensity range from Image bit-depth
Duplicate this image
Select the rule criteria Match All of the following rules
Metadata Does Have C matching 1
Name to assign these images c2
Select the image type Grayscale image
Set intensity range from Image bit-depth
Duplicate this image
Remove this image
Select the rule criteria Match All of the following rules
Metadata Does Have C matching 2
Name to assign these images c3
Select the image type Grayscale image
Set intensity range from Image metadata
Duplicate this image
Remove this image
Update c1 c2 c3
1 G1_8667final4.lif G1_8667final4.lif G1_8667final4.lif
2 G1_8667final4.lif G1_8667final4.lif G1_8667final4.lif
3 G1_8667final4.lif G1_8667final4.lif G1_8667final4.lif
4 G1_8667final4.lif G1_8667final4.lif G1_8667final4.lif
Found 4 image sets

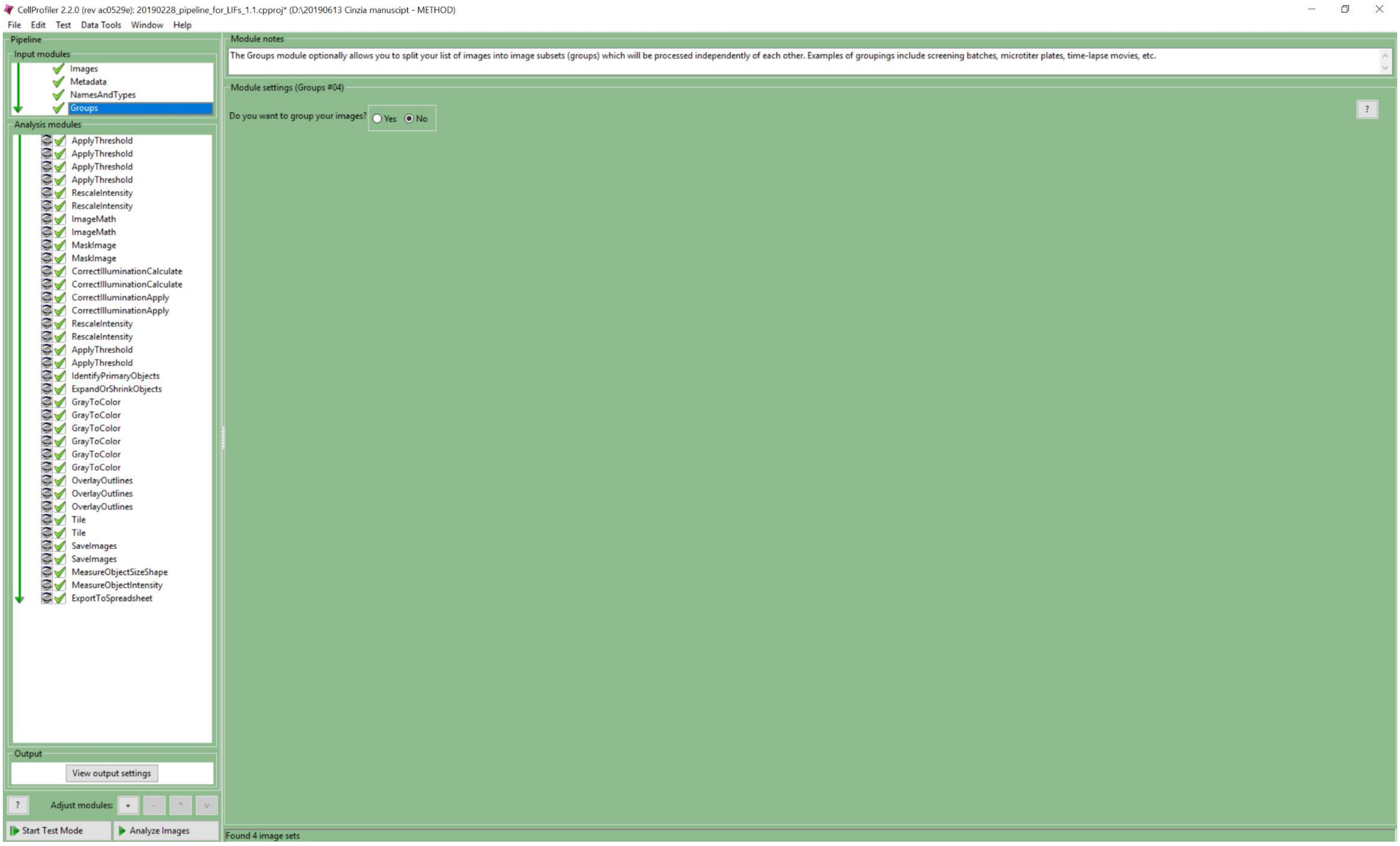
CellProfiler 2.2.0 (rev ac0529e): 20190228_pipeline_for_LIFs_1.1.cpproj* (D:\20190613 Cinzia manuscipt - METHOD)
File Edit Test Data Tools Window Help
Pipeline
Input modules
Images
Metadata
NamesAndTypes
Groups
Analysis modules
ApplyThreshold
ApplyThreshold
ApplyThreshold
ApplyThreshold
RescaleIntensity
RescaleIntensity
ImageMath
ImageMath
MaskImage
MaskImage
CorrectIlluminationCalculate
CorrectIlluminationCalculate
CorrectIlluminationApply
CorrectIlluminationApply
RescaleIntensity
RescaleIntensity
ApplyThreshold
ApplyThreshold
IdentifyPrimaryObjects
ExpandOrShrinkObjects
GrayToColor
GrayToColor
GrayToColor
GrayToColor
GrayToColor
GrayToColor
OverlayOutlines
OverlayOutlines
OverlayOutlines
Tile
Tile
SaveImages
SaveImages
MeasureObjectSizeShape
MeasureObjectIntensity
ExportToSpreadsheet
Output
View output settings
Adjust modules:
Start Test Mode
Analyze Images
Module notes
The Groups module optionally allows you to split your list of images into image subsets (groups) which will be processed independently of each other. Examples of groupings include screening batches, microtiter plates, time-lapse movies, etc.
Module settings (Groups #04)
Do you want to group your images?
Yes
No
Found 4 image sets

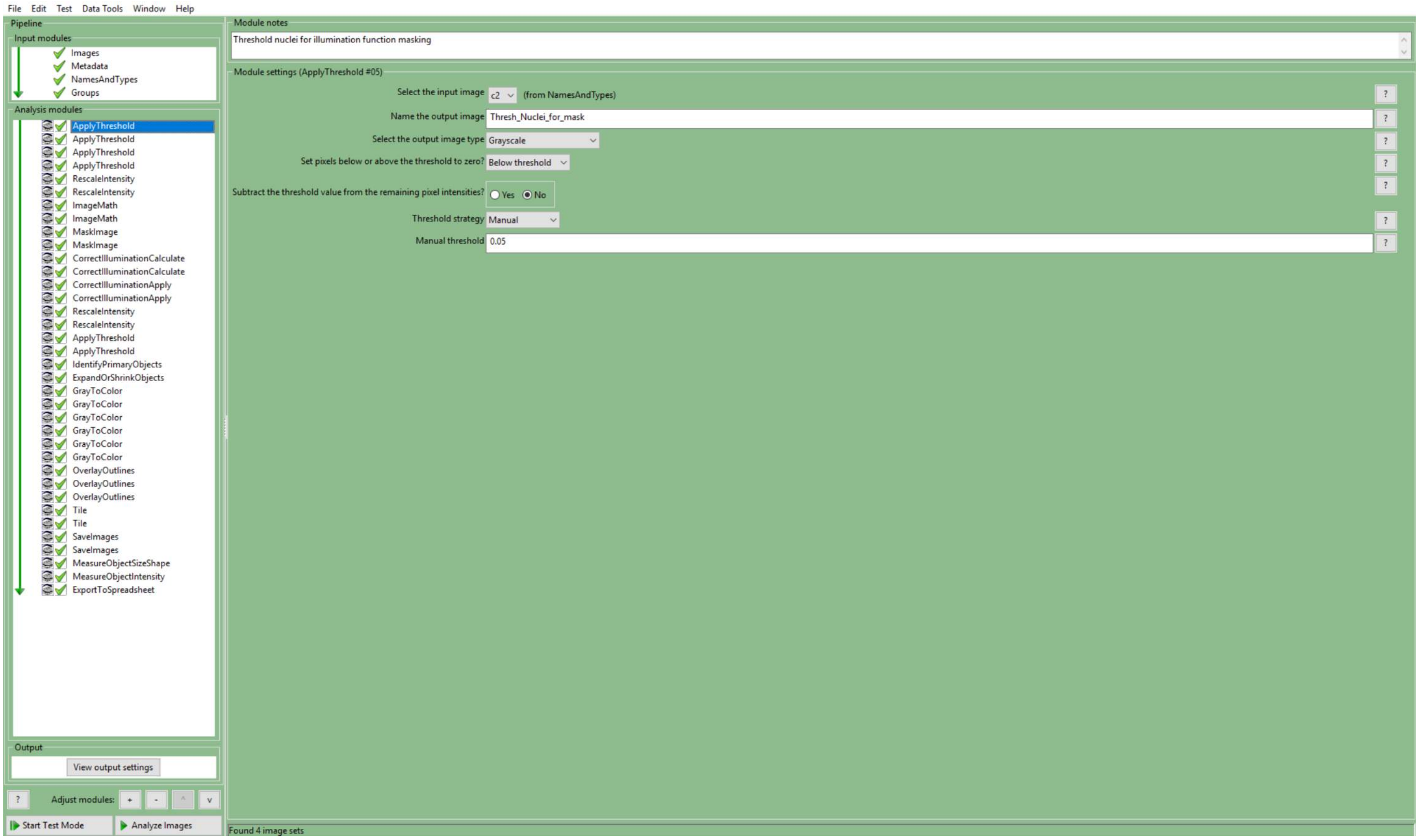
File Edit Test Data Tools Window Help
Pipeline
Input modules
Images
Metadata
NamesAndTypes
Groups
Analysis modules
ApplyThreshold
ApplyThreshold
ApplyThreshold
ApplyThreshold
RescaleIntensity
RescaleIntensity
ImageMath
ImageMath
MaskImage
MaskImage
CorrectIlluminationCalculate
CorrectIlluminationCalculate
CorrectIlluminationApply
CorrectIlluminationApply
RescaleIntensity
RescaleIntensity
ApplyThreshold
ApplyThreshold
IdentifyPrimaryObjects
ExpandOrShrinkObjects
GrayToColor
GrayToColor
GrayToColor
GrayToColor
GrayToColor
GrayToColor
OverlayOutlines
OverlayOutlines
OverlayOutlines
Tile
Tile
SaveImages
SaveImages
MeasureObjectSizeShape
MeasureObjectIntensity
ExportToSpreadsheet
Output
View output settings
Adjust modules:
Start Test Mode
Analyze Images
Module notes
Threshold nuclei for illumination function masking
Module settings (ApplyThreshold #05)
Select the input image c2 (from NamesAndTypes)
Name the output image Thresh_Nuclei_for_mask
Select the output image type Grayscale
Set pixels below or above the threshold to zero? Below threshold
Subtract the threshold value from the remaining pixel intensities? Yes No
Threshold strategy Manual
Manual threshold 0.05
Found 4 image sets

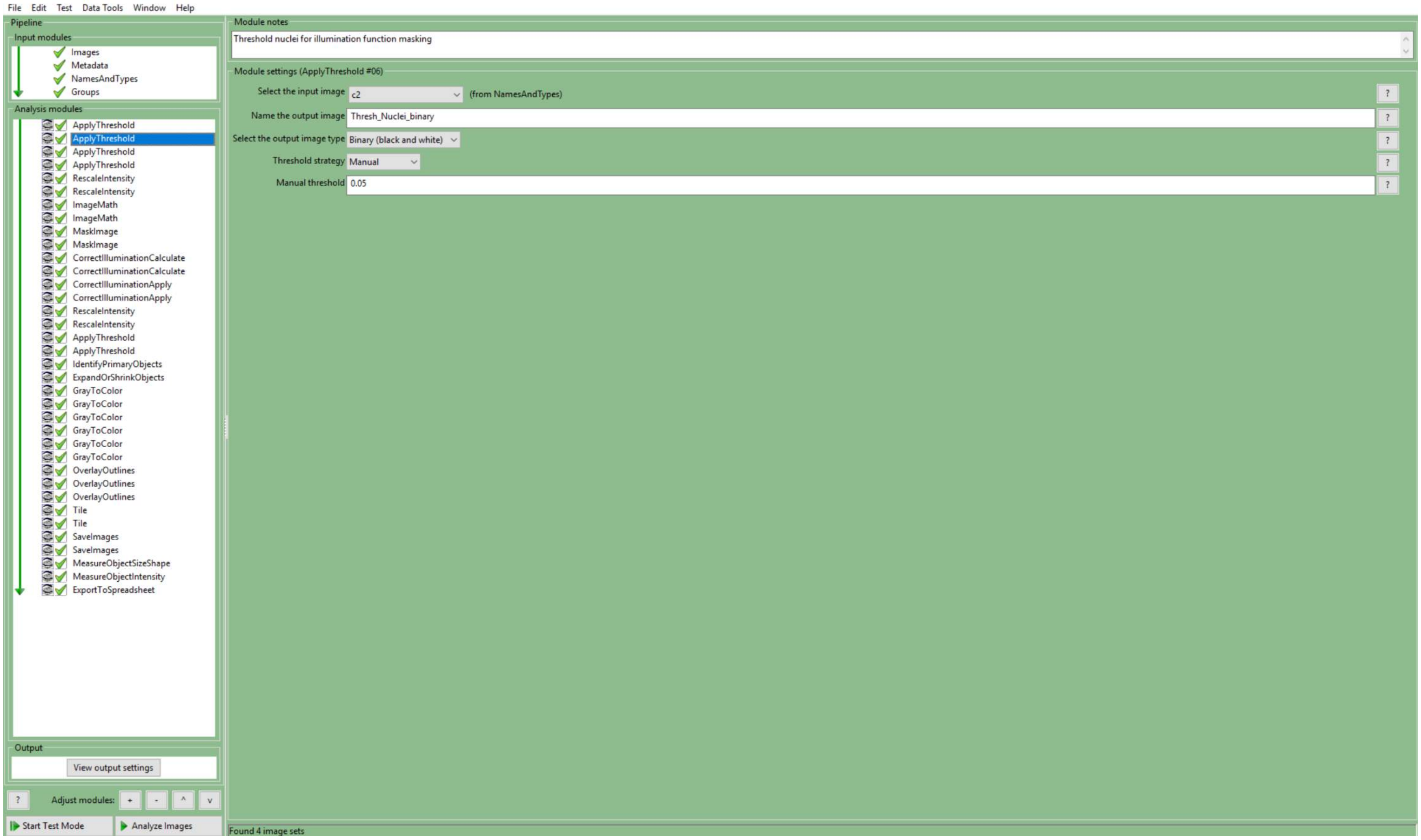
File Edit Test Data Tools Window Help
Pipeline
Input modules
Images
Metadata
NamesAndTypes
Groups
Analysis modules
ApplyThreshold
ApplyThreshold
ApplyThreshold
ApplyThreshold
RescaleIntensity
RescaleIntensity
ImageMath
ImageMath
MaskImage
MaskImage
CorrectIlluminationCalculate
CorrectIlluminationCalculate
CorrectIlluminationApply
CorrectIlluminationApply
RescaleIntensity
RescaleIntensity
ApplyThreshold
ApplyThreshold
IdentifyPrimaryObjects
ExpandOrShrinkObjects
GrayToColor
GrayToColor
GrayToColor
GrayToColor
GrayToColor
GrayToColor
OverlayOutlines
OverlayOutlines
OverlayOutlines
Tile
Tile
SaveImages
SaveImages
MeasureObjectSizeShape
MeasureObjectIntensity
ExportToSpreadsheet
Output
View output settings
Adjust modules:
Start Test Mode
Analyze Images
Module notes
Threshold nuclei for illumination function masking
Module settings (ApplyThreshold #06)
Select the input image
c2
(from NamesAndTypes)
Name the output image
Thresh_Nuclei_binary
Select the output image type
Binary (black and white)
Threshold strategy
Manual
Manual threshold
0.05
Found 4 image sets

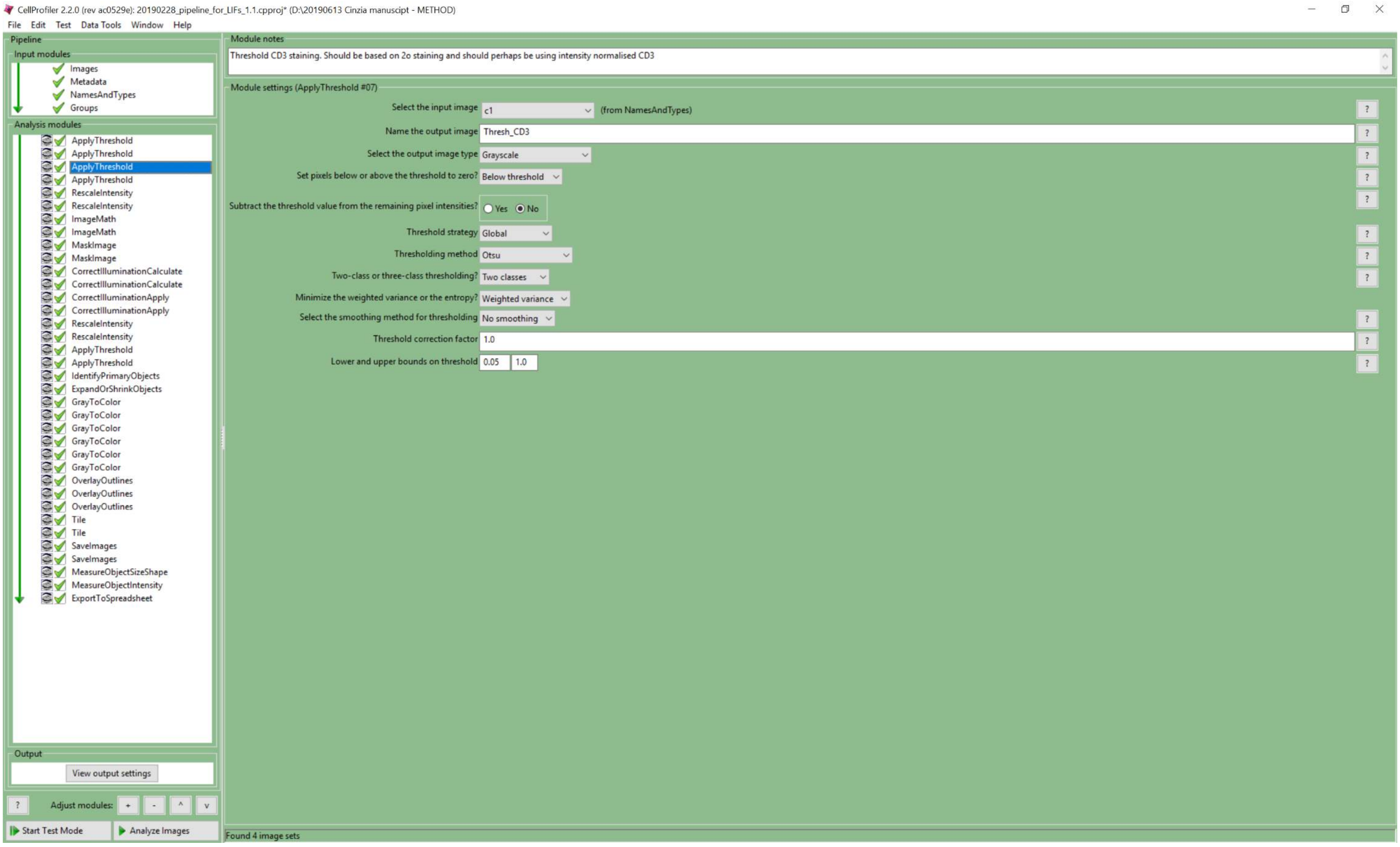
CellProfiler 2.2.0 (rev ac0529e): 20190228_pipeline_for_LIFs_1.1.cpproj* (D:\20190613 Cinzia manuscipt - METHOD)
File Edit Test Data Tools Window Help
Pipeline
Input modules
Images
Metadata
NamesAndTypes
Groups
Analysis modules
ApplyThreshold
ApplyThreshold
ApplyThreshold
ApplyThreshold
RescaleIntensity
RescaleIntensity
ImageMath
ImageMath
MaskImage
MaskImage
CorrectIlluminationCalculate
CorrectIlluminationCalculate
CorrectIlluminationApply
CorrectIlluminationApply
RescaleIntensity
RescaleIntensity
ApplyThreshold
ApplyThreshold
IdentifyPrimaryObjects
ExpandOrShrinkObjects
GrayToColor
GrayToColor
GrayToColor
GrayToColor
GrayToColor
GrayToColor
OverlayOutlines
OverlayOutlines
OverlayOutlines
Tile
Tile
SaveImages
SaveImages
MeasureObjectSizeShape
MeasureObjectIntensity
ExportToSpreadsheet
Output
View output settings
Adjust modules:
Start Test Mode
Analyze Images
Module notes
Threshold CD3 staining. Should be based on 2o staining and should perhaps be using intensity normalised CD3
Module settings (ApplyThreshold #07)
Select the input image c1 (from NamesAndTypes)
Name the output image Thresh_CD3
Select the output image type Grayscale
Set pixels below or above the threshold to zero? Below threshold
Subtract the threshold value from the remaining pixel intensities? Yes No
Threshold strategy Global
Thresholding method Otsu
Two-class or three-class thresholding? Two classes
Minimize the weighted variance or the entropy? Weighted variance
Select the smoothing method for thresholding No smoothing
Threshold correction factor 1.0
Lower and upper bounds on threshold 0.05 1.0
Found 4 image sets

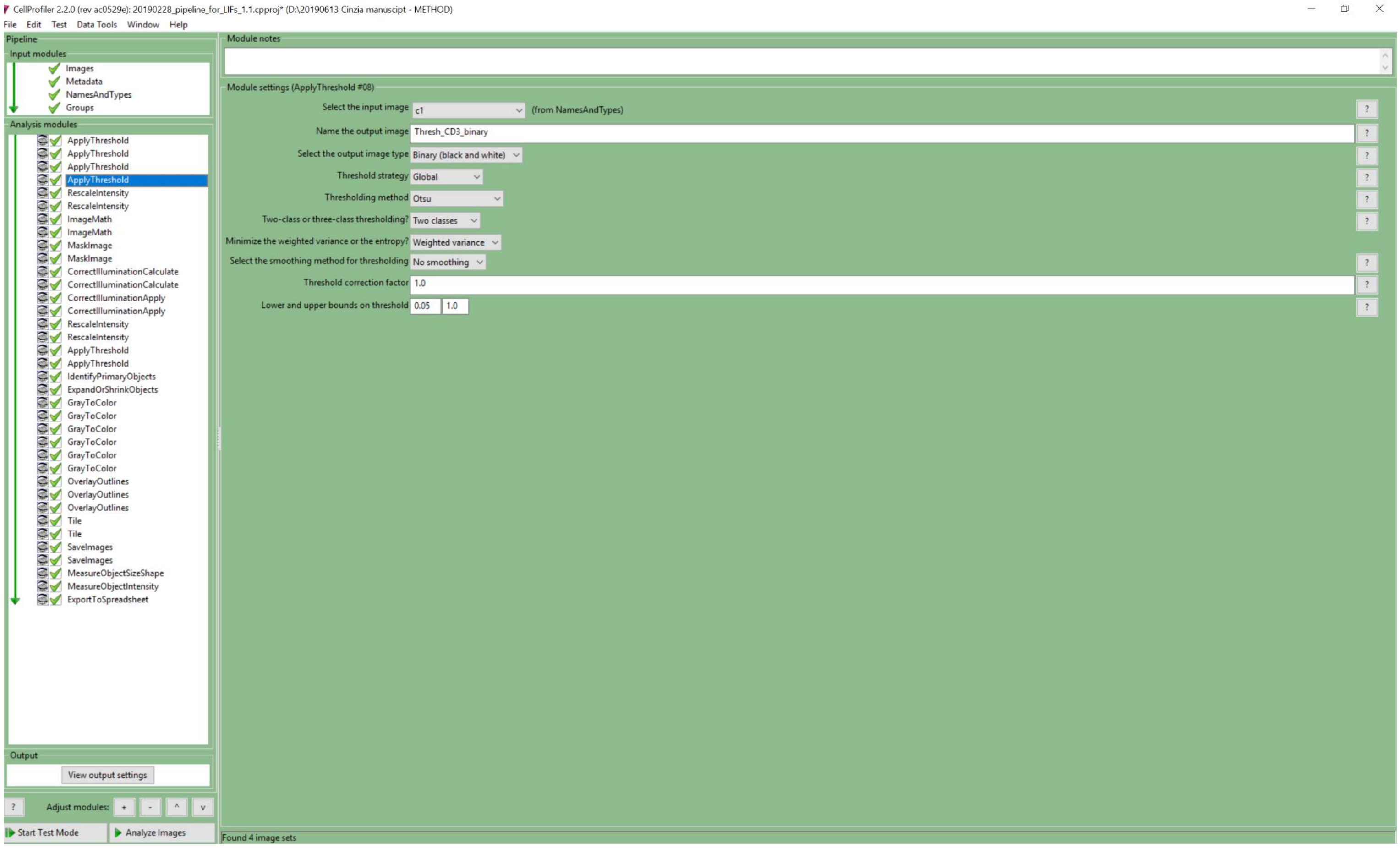
CellProfiler 2.2.0 (rev ac0529e): 20190228_pipeline_for_LIFs_1.1.cpproj* (D:\20190613 Cinzia manuscipt - METHOD)
File Edit Test Data Tools Window Help
Pipeline
Input modules
Images
Metadata
NamesAndTypes
Groups
Analysis modules
ApplyThreshold
ApplyThreshold
ApplyThreshold
ApplyThreshold
RescaleIntensity
RescaleIntensity
ImageMath
ImageMath
MaskImage
MaskImage
CorrectIlluminationCalculate
CorrectIlluminationCalculate
CorrectIlluminationApply
CorrectIlluminationApply
RescaleIntensity
RescaleIntensity
ApplyThreshold
ApplyThreshold
IdentifyPrimaryObjects
ExpandOrShrinkObjects
GrayToColor
GrayToColor
GrayToColor
GrayToColor
GrayToColor
GrayToColor
OverlayOutlines
OverlayOutlines
OverlayOutlines
Tile
Tile
SaveImages
SaveImages
MeasureObjectSizeShape
MeasureObjectIntensity
ExportToSpreadsheet
Output
View output settings
Adjust modules:
Start Test Mode
Analyze Images
Module notes
Module settings (ApplyThreshold #08)
Select the input image c1 (from NamesAndTypes)
Name the output image Thresh_CD3_binary
Select the output image type Binary (black and white)
Threshold strategy Global
Thresholding method Otsu
Two-class or three-class thresholding? Two classes
Minimize the weighted variance or the entropy? Weighted variance
Select the smoothing method for thresholding No smoothing
Threshold correction factor 1.0
Lower and upper bounds on threshold 0.05 1.0
Found 4 image sets

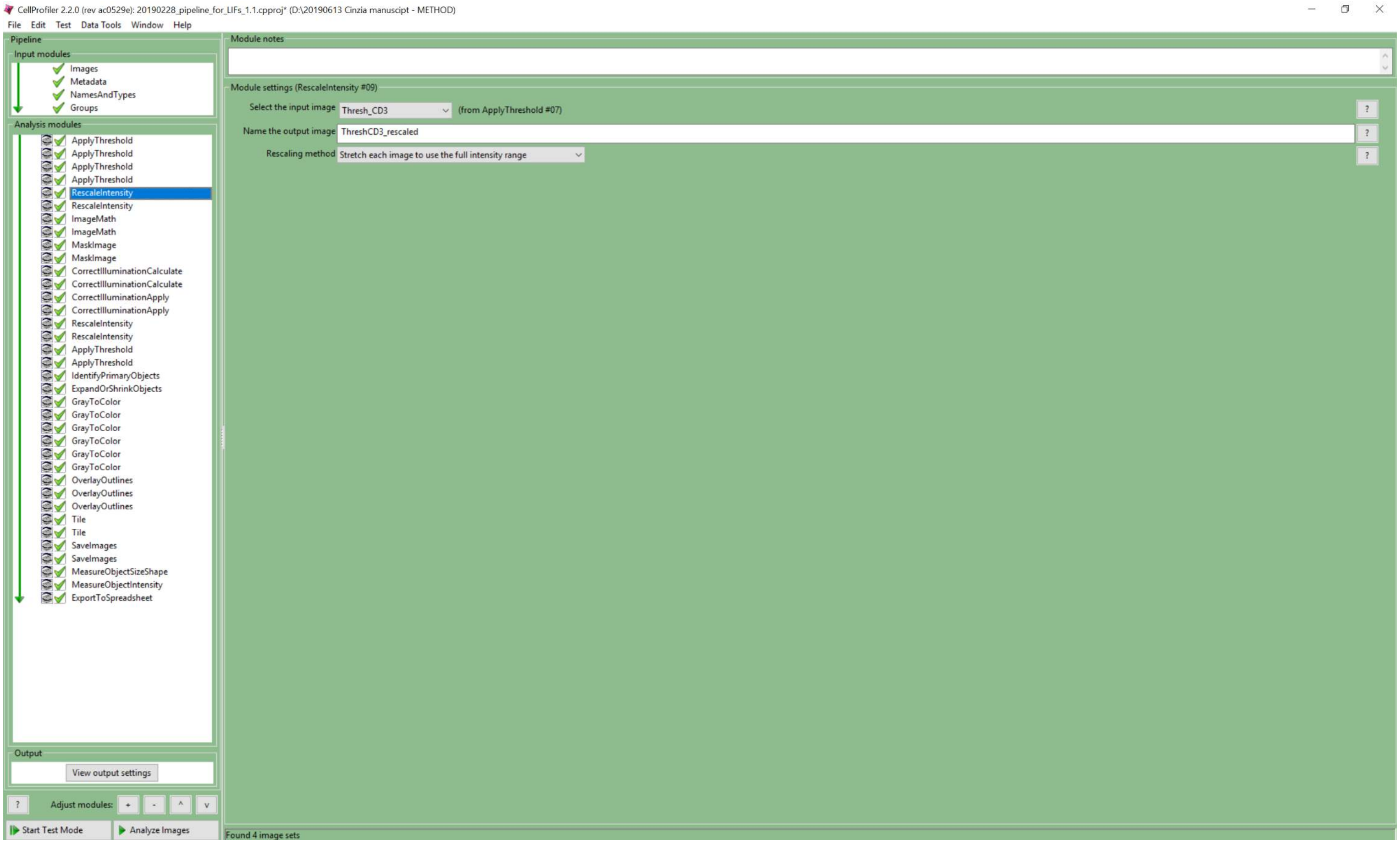
CellProfiler 2.2.0 (rev ac0529e): 20190228_pipeline_for_LIFs_1.1.cpproj* (D:\20190613 Cinzia manuscipt - METHOD)
File Edit Test Data Tools Window Help
Pipeline
Input modules
Images
Metadata
NamesAndTypes
Groups
Analysis modules
ApplyThreshold
ApplyThreshold
ApplyThreshold
ApplyThreshold
RescaleIntensity
RescaleIntensity
ImageMath
ImageMath
MaskImage
MaskImage
CorrectIlluminationCalculate
CorrectIlluminationCalculate
CorrectIlluminationApply
CorrectIlluminationApply
RescaleIntensity
RescaleIntensity
ApplyThreshold
ApplyThreshold
IdentifyPrimaryObjects
ExpandOrShrinkObjects
GrayToColor
GrayToColor
GrayToColor
GrayToColor
GrayToColor
GrayToColor
OverlayOutlines
OverlayOutlines
OverlayOutlines
Tile
Tile
SaveImages
SaveImages
MeasureObjectSizeShape
MeasureObjectIntensity
ExportToSpreadsheet
Output
View output settings
Adjust modules:
Start Test Mode
Analyze Images
Module notes
Module settings (RescaleIntensity #09)
Select the input image
Thresh_CD3
(from ApplyThreshold #07)
Name the output image
ThreshCD3_rescaled
Rescaling method
Stretch each image to use the full intensity range
Found 4 image sets

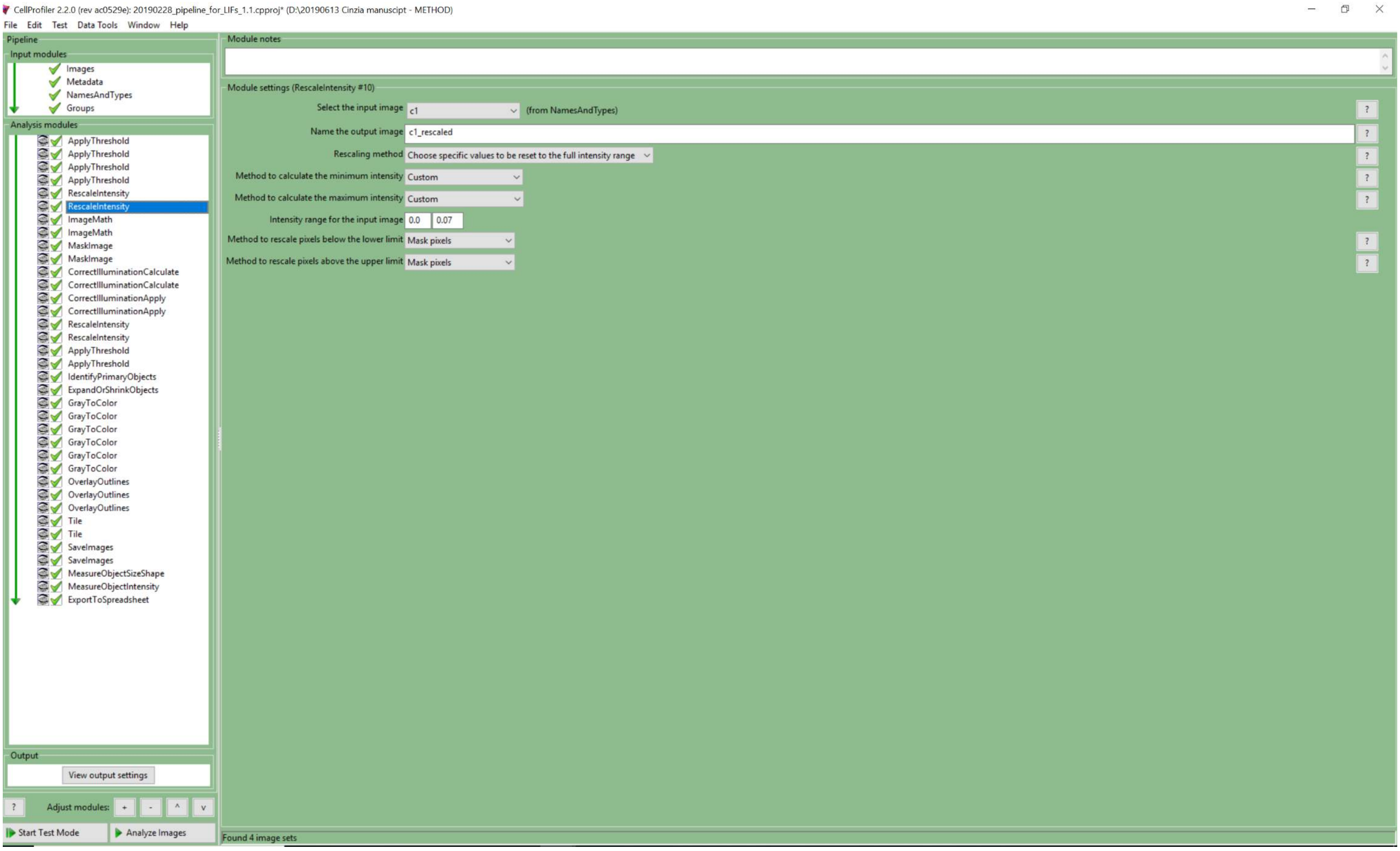
CellProfiler 2.2.0 (rev ac0529e): 20190228_pipeline_for_LIFs_1.1.cpproj* (D:\20190613 Cinzia manuscipt - METHOD)
File Edit Test Data Tools Window Help
Pipeline
Input modules
Images
Metadata
NamesAndTypes
Groups
Analysis modules
ApplyThreshold
ApplyThreshold
ApplyThreshold
ApplyThreshold
RescaleIntensity
RescaleIntensity
ImageMath
ImageMath
MaskImage
MaskImage
CorrectIlluminationCalculate
CorrectIlluminationCalculate
CorrectIlluminationApply
CorrectIlluminationApply
RescaleIntensity
RescaleIntensity
ApplyThreshold
ApplyThreshold
IdentifyPrimaryObjects
ExpandOrShrinkObjects
GrayToColor
GrayToColor
GrayToColor
GrayToColor
GrayToColor
GrayToColor
OverlayOutlines
OverlayOutlines
OverlayOutlines
Tile
Tile
SaveImages
SaveImages
MeasureObjectSizeShape
MeasureObjectIntensity
ExportToSpreadsheet
Output
View output settings
Adjust modules:
Start Test Mode
Analyze Images
Module notes
Module settings (RescaleIntensity #10)
Select the input image
c1
(from NamesAndTypes)
Name the output image
c1_rescaled
Rescaling method
Choose specific values to be reset to the full intensity range
Method to calculate the minimum intensity
Custom
Method to calculate the maximum intensity
Custom
Intensity range for the input image
0.0
0.07
Method to rescale pixels below the lower limit
Mask pixels
Method to rescale pixels above the upper limit
Mask pixels
Found 4 image sets

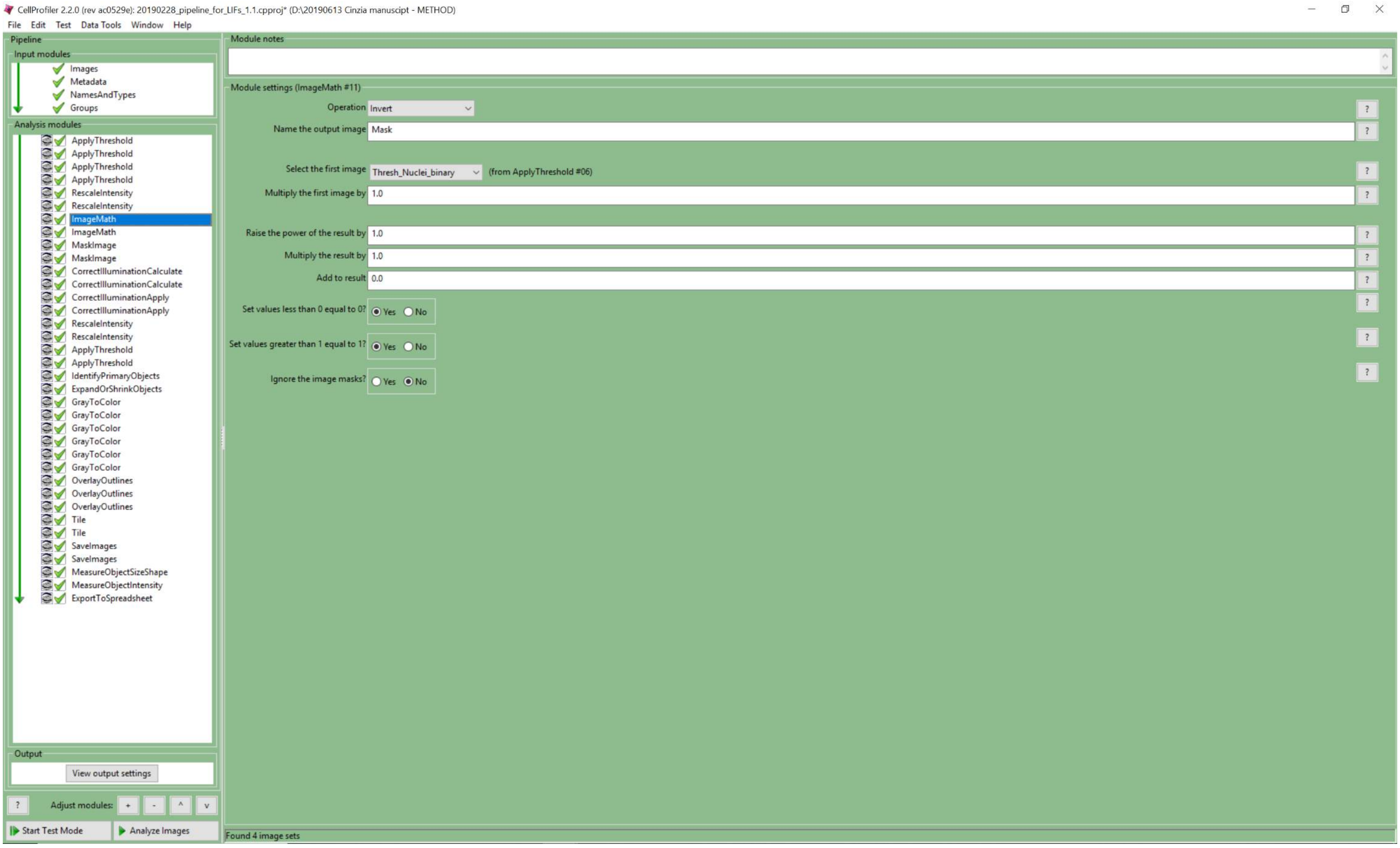
CellProfiler 2.2.0 (rev ac0529e): 20190228_pipeline_for_LIFs_1.1.cpproj* (D:\20190613 Cinzia manuscipt - METHOD)
File Edit Test Data Tools Window Help
Pipeline
Input modules
Images
Metadata
NamesAndTypes
Groups
Analysis modules
ApplyThreshold
ApplyThreshold
ApplyThreshold
ApplyThreshold
RescaleIntensity
RescaleIntensity
ImageMath
ImageMath
MaskImage
MaskImage
CorrectIlluminationCalculate
CorrectIlluminationCalculate
CorrectIlluminationApply
CorrectIlluminationApply
RescaleIntensity
RescaleIntensity
ApplyThreshold
ApplyThreshold
IdentifyPrimaryObjects
ExpandOrShrinkObjects
GrayToColor
GrayToColor
GrayToColor
GrayToColor
GrayToColor
GrayToColor
OverlayOutlines
OverlayOutlines
OverlayOutlines
Tile
Tile
SaveImages
SaveImages
MeasureObjectSizeShape
MeasureObjectIntensity
ExportToSpreadsheet
Output
View output settings
Adjust modules: + - ^ v
Start Test Mode
Analyze Images
Module notes
Module settings (ImageMath #11)
Operation Invert
Name the output image Mask
Select the first image Thresh_Nuclei_binary (from ApplyThreshold #06)
Multiply the first image by 1.0
Raise the power of the result by 1.0
Multiply the result by 1.0
Add to result 0.0
Set values less than 0 equal to 0? Yes No
Set values greater than 1 equal to 1? Yes No
Ignore the image masks? Yes No
Found 4 image sets

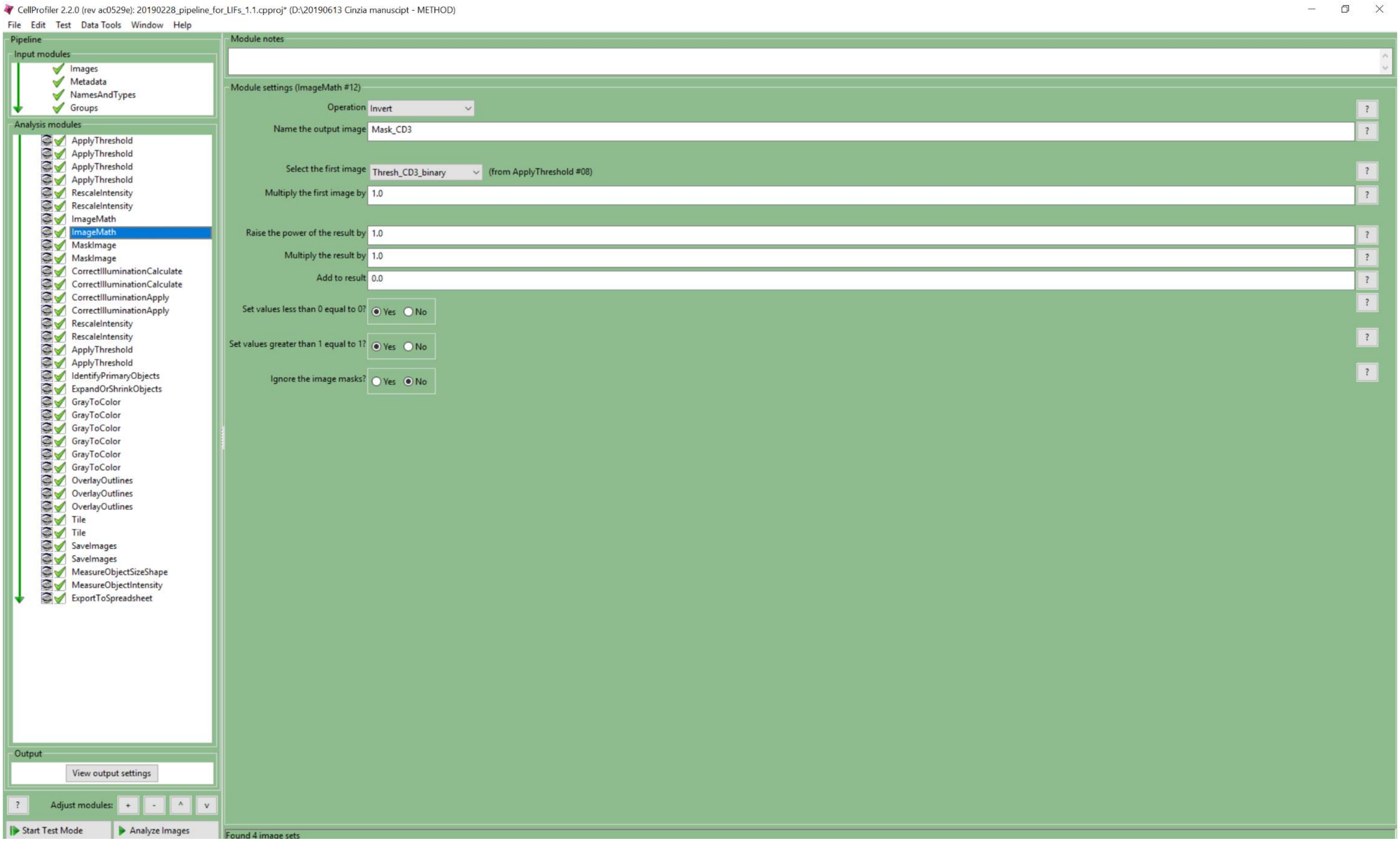
CellProfiler 2.2.0 (rev ac0529e): 20190228_pipeline_for_LIFs_1.1.cpproj* (D:\20190613 Cinzia manuscipt - METHOD)
File Edit Test Data Tools Window Help
Pipeline
Input modules
Images
Metadata
NamesAndTypes
Groups
Analysis modules
ApplyThreshold
ApplyThreshold
ApplyThreshold
ApplyThreshold
RescaleIntensity
RescaleIntensity
ImageMath
ImageMath
MaskImage
MaskImage
CorrectIlluminationCalculate
CorrectIlluminationCalculate
CorrectIlluminationApply
CorrectIlluminationApply
RescaleIntensity
RescaleIntensity
ApplyThreshold
ApplyThreshold
IdentifyPrimaryObjects
ExpandOrShrinkObjects
GrayToColor
GrayToColor
GrayToColor
GrayToColor
GrayToColor
GrayToColor
OverlayOutlines
OverlayOutlines
OverlayOutlines
Tile
Tile
SaveImages
SaveImages
MeasureObjectSizeShape
MeasureObjectIntensity
ExportToSpreadsheet
Output
View output settings
Adjust modules:
Start Test Mode
Analyze Images
Module notes
Module settings (ImageMath #12)
Operation Invert
Name the output image Mask_CD3
Select the first image Thresh_CD3_binary (from ApplyThreshold #08)
Multiply the first image by 1.0
Raise the power of the result by 1.0
Multiply the result by 1.0
Add to result 0.0
Set values less than 0 equal to 0? Yes No
Set values greater than 1 equal to 1? Yes No
Ignore the image masks? Yes No
Found 4 image sets

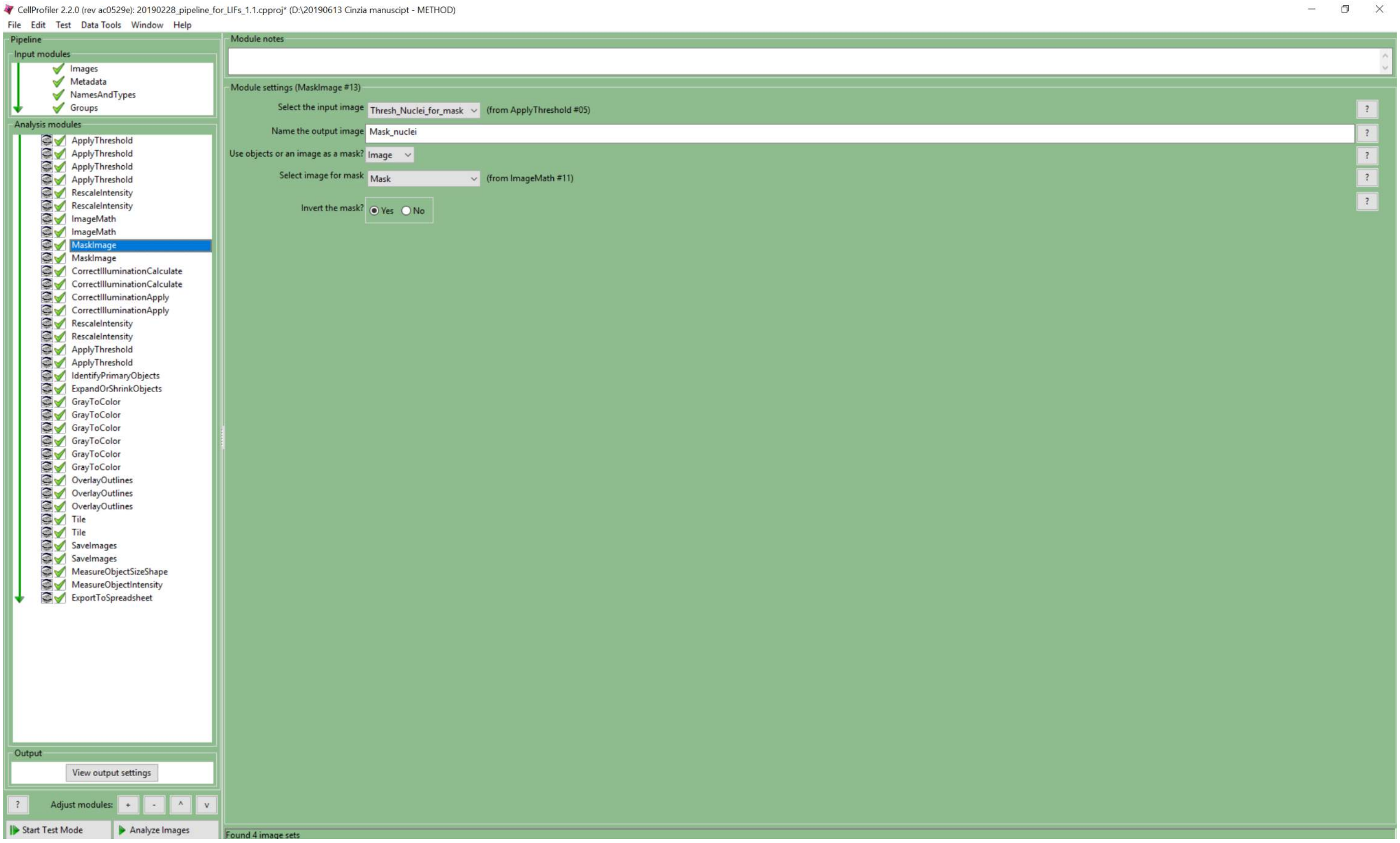
CellProfiler 2.2.0 (rev ac0529e): 20190228_pipeline_for_LIFs_1.1.cpproj* (D:\20190613 Cinzia manuscipt - METHOD)
File Edit Test Data Tools Window Help
Pipeline
Input modules
Images
Metadata
NamesAndTypes
Groups
Analysis modules
ApplyThreshold
ApplyThreshold
ApplyThreshold
ApplyThreshold
RescaleIntensity
RescaleIntensity
ImageMath
ImageMath
MaskImage
MaskImage
CorrectIlluminationCalculate
CorrectIlluminationCalculate
CorrectIlluminationApply
CorrectIlluminationApply
RescaleIntensity
RescaleIntensity
ApplyThreshold
ApplyThreshold
IdentifyPrimaryObjects
ExpandOrShrinkObjects
GrayToColor
GrayToColor
GrayToColor
GrayToColor
GrayToColor
GrayToColor
OverlayOutlines
OverlayOutlines
OverlayOutlines
Tile
Tile
SaveImages
SaveImages
MeasureObjectSizeShape
MeasureObjectIntensity
ExportToSpreadsheet
Output
View output settings
Adjust modules:
Start Test Mode
Analyze Images
Module notes
Module settings (MaskImage #13)
Select the input image
Thresh_Nuclei_for_mask
(from ApplyThreshold #05)
Name the output image
Mask_nuclei
Use objects or an image as a mask?
Image
Select image for mask
Mask
(from ImageMath #11)
Invert the mask?
Yes
No
Found 4 image sets

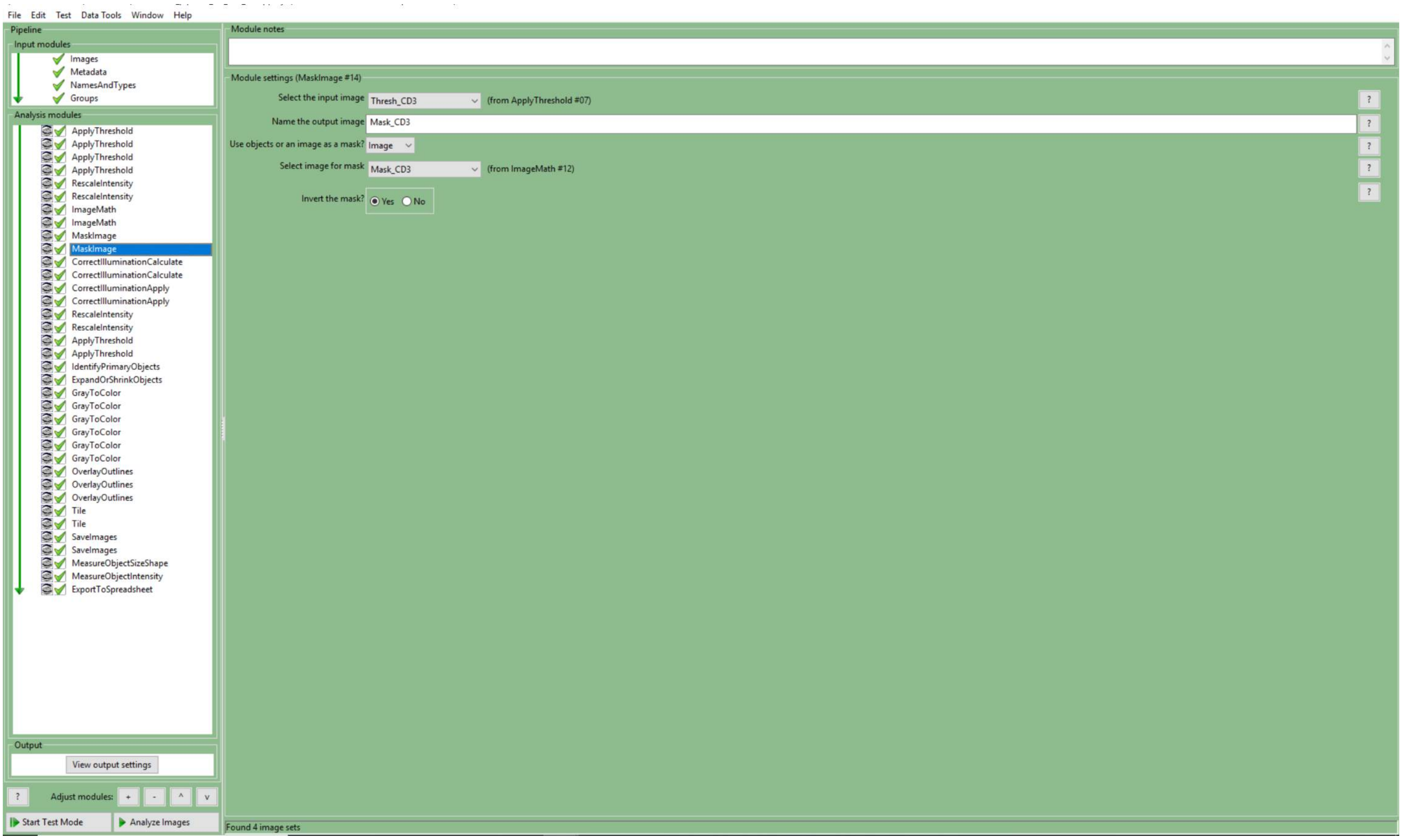
File Edit Test Data Tools Window Help
Pipeline
Input modules
Images
Metadata
NamesAndTypes
Groups
Analysis modules
ApplyThreshold
ApplyThreshold
ApplyThreshold
ApplyThreshold
RescaleIntensity
RescaleIntensity
ImageMath
ImageMath
MaskImage
MaskImage
CorrectIlluminationCalculate
CorrectIlluminationCalculate
CorrectIlluminationApply
CorrectIlluminationApply
RescaleIntensity
RescaleIntensity
ApplyThreshold
ApplyThreshold
IdentifyPrimaryObjects
ExpandOrShrinkObjects
GrayToColor
GrayToColor
GrayToColor
GrayToColor
GrayToColor
GrayToColor
OverlayOutlines
OverlayOutlines
OverlayOutlines
Tile
Tile
SaveImages
SaveImages
MeasureObjectSizeShape
MeasureObjectIntensity
ExportToSpreadsheet
Output
View output settings
Adjust modules:
Start Test Mode
Analyze Images
Module notes
Module settings (MaskImage #14)
Select the input image
Thresh_CD3
(from ApplyThreshold #07)
Name the output image
Mask_CD3
Use objects or an image as a mask?
Image
Select image for mask
Mask_CD3
(from ImageMath #12)
Invert the mask?
Yes
No
Found 4 image sets

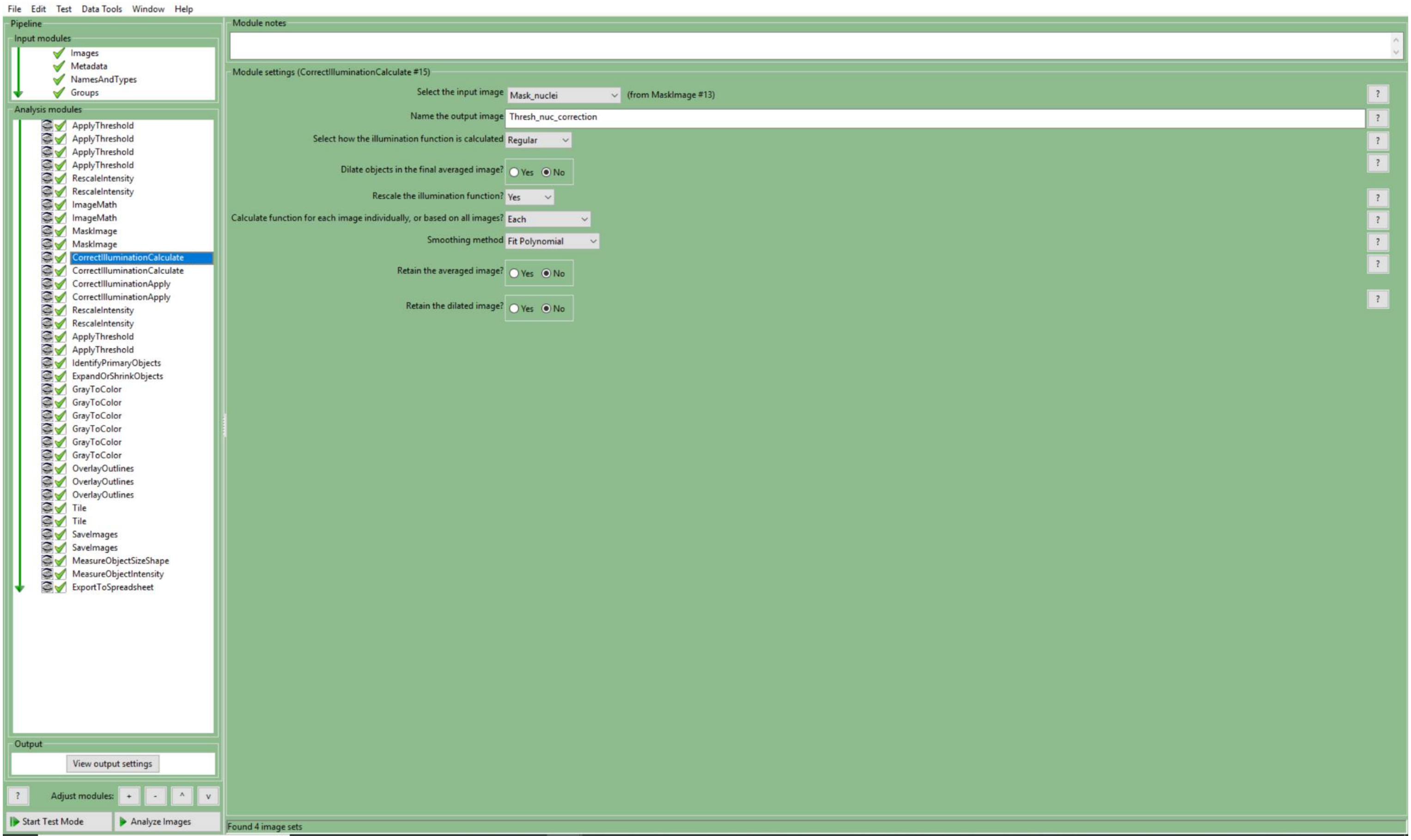
File Edit Test Data Tools Window Help
Pipeline
Input modules
Images
Metadata
NamesAndTypes
Groups
Analysis modules
ApplyThreshold
ApplyThreshold
ApplyThreshold
ApplyThreshold
RescaleIntensity
RescaleIntensity
ImageMath
ImageMath
MaskImage
MaskImage
CorrectIlluminationCalculate
CorrectIlluminationCalculate
CorrectIlluminationApply
CorrectIlluminationApply
RescaleIntensity
RescaleIntensity
ApplyThreshold
ApplyThreshold
IdentifyPrimaryObjects
ExpandOrShrinkObjects
GrayToColor
GrayToColor
GrayToColor
GrayToColor
GrayToColor
GrayToColor
OverlayOutlines
OverlayOutlines
OverlayOutlines
Tile
Tile
SaveImages
SaveImages
MeasureObjectSizeShape
MeasureObjectIntensity
ExportToSpreadsheet
Output
View output settings
Adjust modules:
Start Test Mode
Analyze Images
Module notes
Module settings (CorrectIlluminationCalculate #15)
Select the input image
Mask_nuclei
(from MaskImage #13)
Name the output image
Thresh_nuc_correction
Select how the illumination function is calculated
Regular
Dilate objects in the final averaged image?
Yes No
Rescale the illumination function?
Yes
Calculate function for each image individually, or based on all images?
Each
Smoothing method
Fit Polynomial
Retain the averaged image?
Yes No
Retain the dilated image?
Yes No
Found 4 image sets

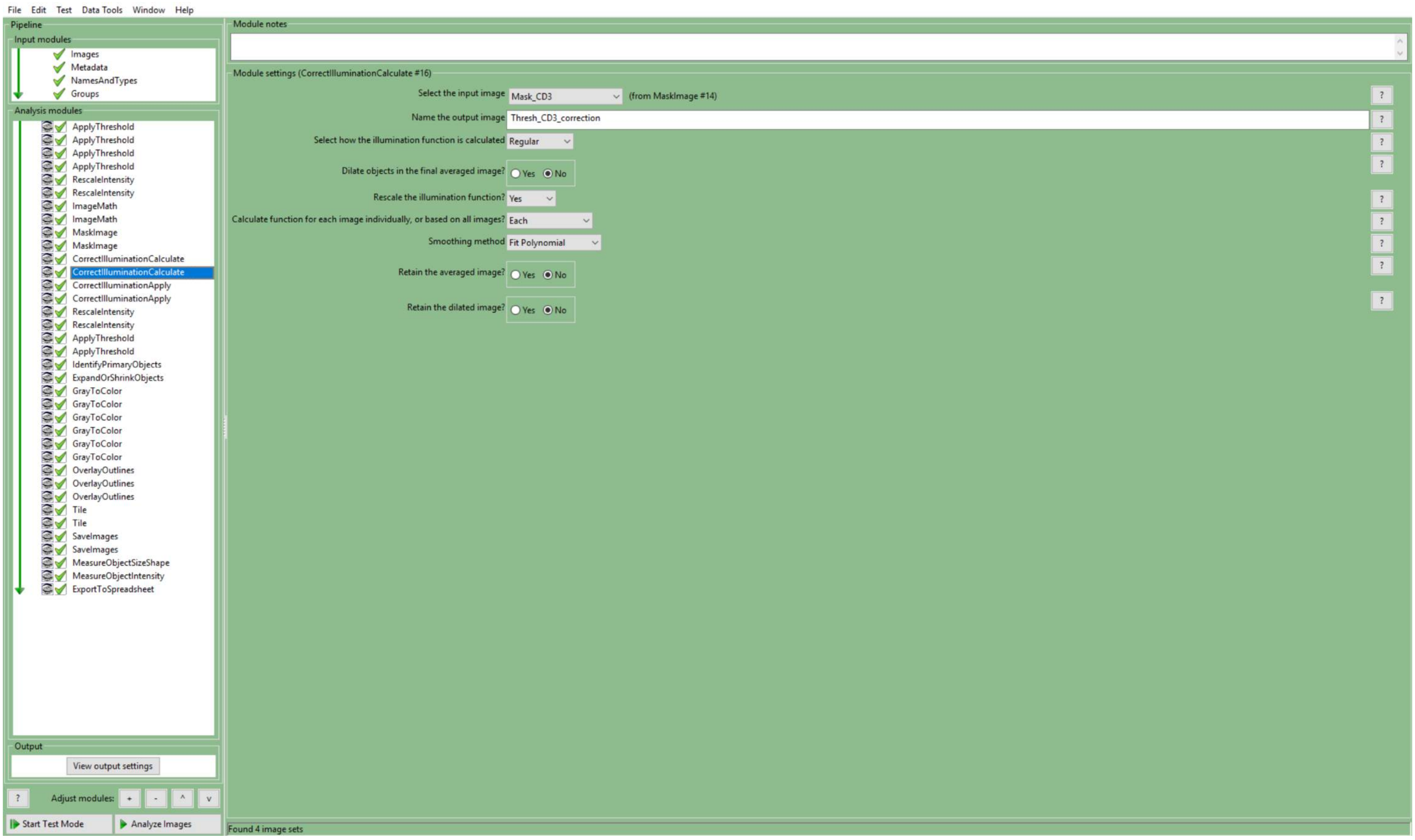
File Edit Test Data Tools Window Help
Pipeline
Input modules
Images
Metadata
NamesAndTypes
Groups
Analysis modules
ApplyThreshold
ApplyThreshold
ApplyThreshold
ApplyThreshold
RescaleIntensity
RescaleIntensity
ImageMath
ImageMath
MaskImage
MaskImage
CorrectIlluminationCalculate
CorrectIlluminationCalculate
CorrectIlluminationApply
CorrectIlluminationApply
RescaleIntensity
RescaleIntensity
ApplyThreshold
ApplyThreshold
IdentifyPrimaryObjects
ExpandOrShrinkObjects
GrayToColor
GrayToColor
GrayToColor
GrayToColor
GrayToColor
GrayToColor
OverlayOutlines
OverlayOutlines
OverlayOutlines
Tile
Tile
SaveImages
SaveImages
MeasureObjectSizeShape
MeasureObjectIntensity
ExportToSpreadsheet
Output
View output settings
Adjust modules:
Start Test Mode
Analyze Images
Module notes
Module settings (CorrectIlluminationCalculate #16)
Select the input image
Mask_CD3
(from MaskImage #14)
Name the output image
Thresh_CD3_correction
Select how the illumination function is calculated
Regular
Dilate objects in the final averaged image?
Yes No
Rescale the illumination function?
Yes
Calculate function for each image individually, or based on all images?
Each
Smoothing method
Fit Polynomial
Retain the averaged image?
Yes No
Retain the dilated image?
Yes No
Found 4 image sets

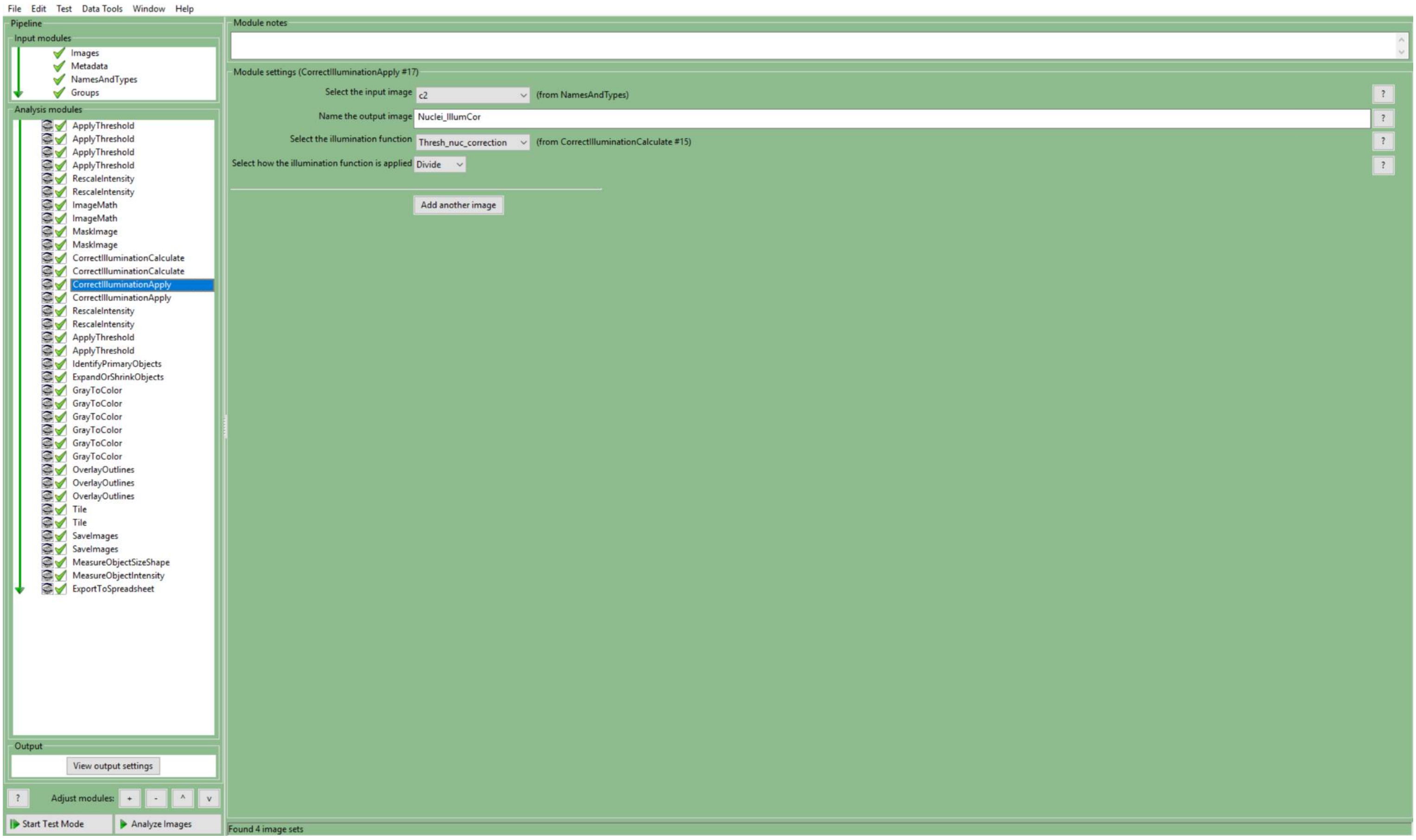
File Edit Test Data Tools Window Help
Pipeline
Input modules
Images
Metadata
NamesAndTypes
Groups
Analysis modules
ApplyThreshold
ApplyThreshold
ApplyThreshold
ApplyThreshold
RescaleIntensity
RescaleIntensity
ImageMath
ImageMath
MaskImage
MaskImage
CorrectIlluminationCalculate
CorrectIlluminationCalculate
CorrectIlluminationApply
CorrectIlluminationApply
RescaleIntensity
RescaleIntensity
ApplyThreshold
ApplyThreshold
IdentifyPrimaryObjects
ExpandOrShrinkObjects
GrayToColor
GrayToColor
GrayToColor
GrayToColor
GrayToColor
GrayToColor
OverlayOutlines
OverlayOutlines
OverlayOutlines
Tile
Tile
SaveImages
SaveImages
MeasureObjectSizeShape
MeasureObjectIntensity
ExportToSpreadsheet
Output
View output settings
Adjust modules:
Start Test Mode
Analyze Images
Module notes
Module settings (CorrectIlluminationApply #17)
Select the input image
c2
(from NamesAndTypes)
Name the output image
Nuclei_IllumCor
Select the illumination function
Thresh_nuc_correction
(from CorrectIlluminationCalculate #15)
Select how the illumination function is applied
Divide
Add another image
Found 4 image sets

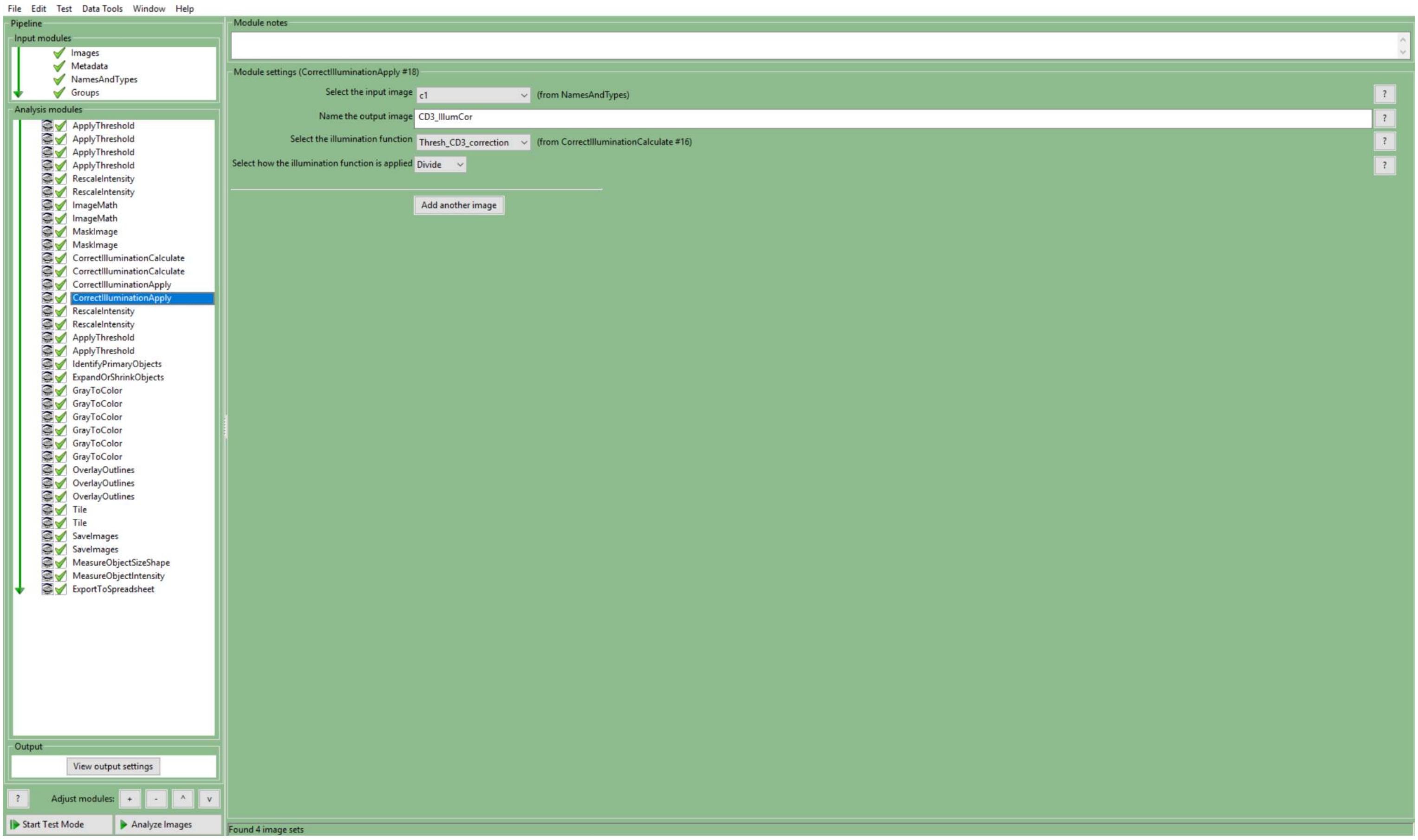
File Edit Test Data Tools Window Help
Pipeline
Input modules
Images
Metadata
NamesAndTypes
Groups
Analysis modules
ApplyThreshold
ApplyThreshold
ApplyThreshold
ApplyThreshold
RescaleIntensity
RescaleIntensity
ImageMath
ImageMath
MaskImage
MaskImage
CorrectIlluminationCalculate
CorrectIlluminationCalculate
CorrectIlluminationApply
CorrectIlluminationApply
RescaleIntensity
RescaleIntensity
ApplyThreshold
ApplyThreshold
IdentifyPrimaryObjects
ExpandOrShrinkObjects
GrayToColor
GrayToColor
GrayToColor
GrayToColor
GrayToColor
GrayToColor
OverlayOutlines
OverlayOutlines
OverlayOutlines
Tile
Tile
SaveImages
SaveImages
MeasureObjectSizeShape
MeasureObjectIntensity
ExportToSpreadsheet
Output
View output settings
Adjust modules:
Start Test Mode
Analyze Images
Module notes
Module settings (CorrectIlluminationApply #18)
Select the input image
c1
(from NamesAndTypes)
Name the output image
CD3_IllumCor
Select the illumination function
Thresh_CD3_correction
(from CorrectIlluminationCalculate #16)
Select how the illumination function is applied
Divide
Add another image
Found 4 image sets

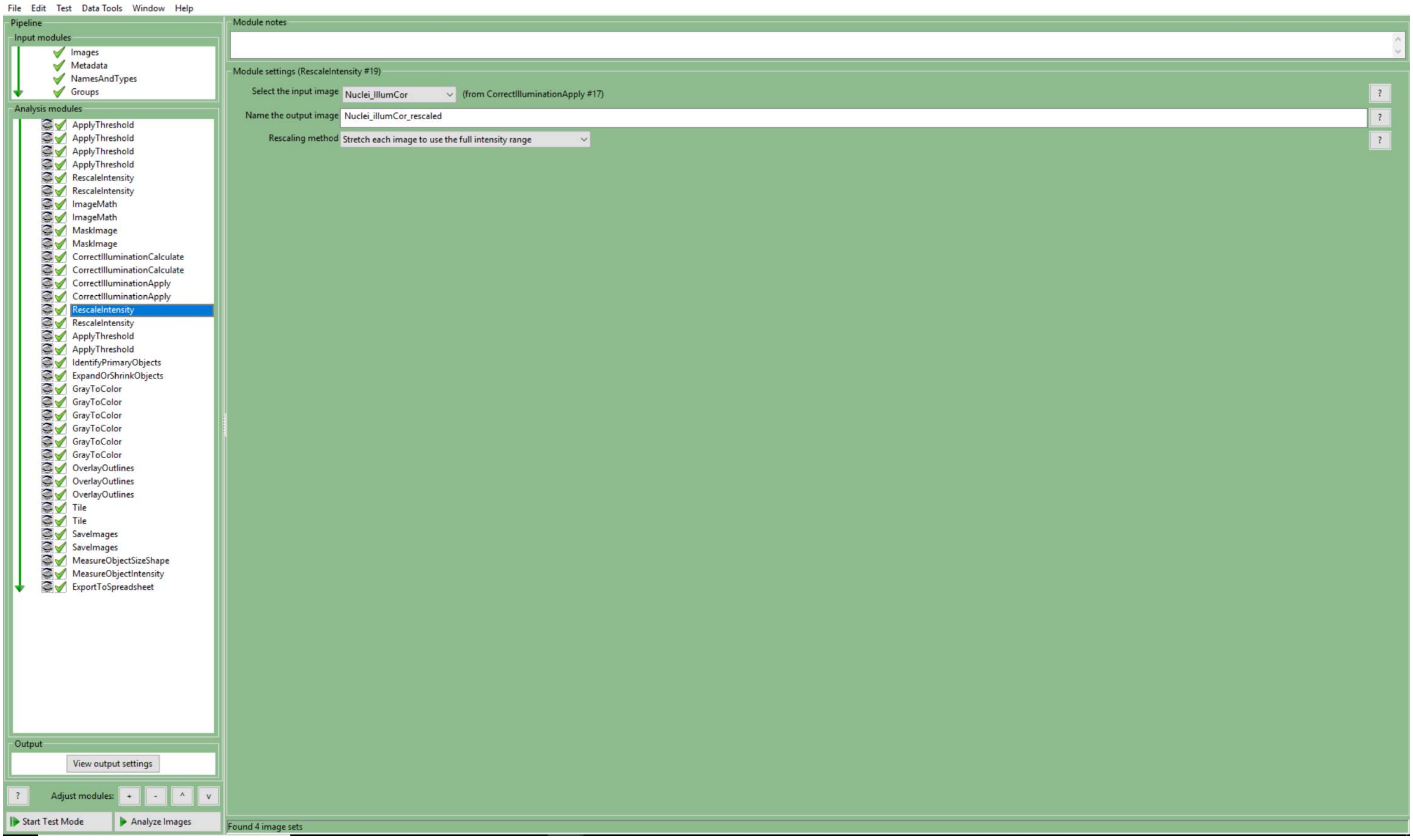
File Edit Test Data Tools Window Help
Pipeline
Input modules
Images
Metadata
NamesAndTypes
Groups
Analysis modules
ApplyThreshold
ApplyThreshold
ApplyThreshold
ApplyThreshold
RescaleIntensity
RescaleIntensity
ImageMath
ImageMath
MaskImage
MaskImage
CorrectIlluminationCalculate
CorrectIlluminationCalculate
CorrectIlluminationApply
CorrectIlluminationApply
RescaleIntensity
RescaleIntensity
ApplyThreshold
ApplyThreshold
IdentifyPrimaryObjects
ExpandOrShrinkObjects
GrayToColor
GrayToColor
GrayToColor
GrayToColor
GrayToColor
GrayToColor
OverlayOutlines
OverlayOutlines
OverlayOutlines
Tile
Tile
SaveImages
SaveImages
MeasureObjectSizeShape
MeasureObjectIntensity
ExportToSpreadsheet
Output
View output settings
Adjust modules:
Start Test Mode
Analyze Images
Module notes
Module settings (RescaleIntensity #19)
Select the input image
Nuclei_IllumCor
(from CorrectIlluminationApply #17)
Name the output image
Nuclei_illumCor_rescaled
Rescaling method
Stretch each image to use the full intensity range
Found 4 image sets

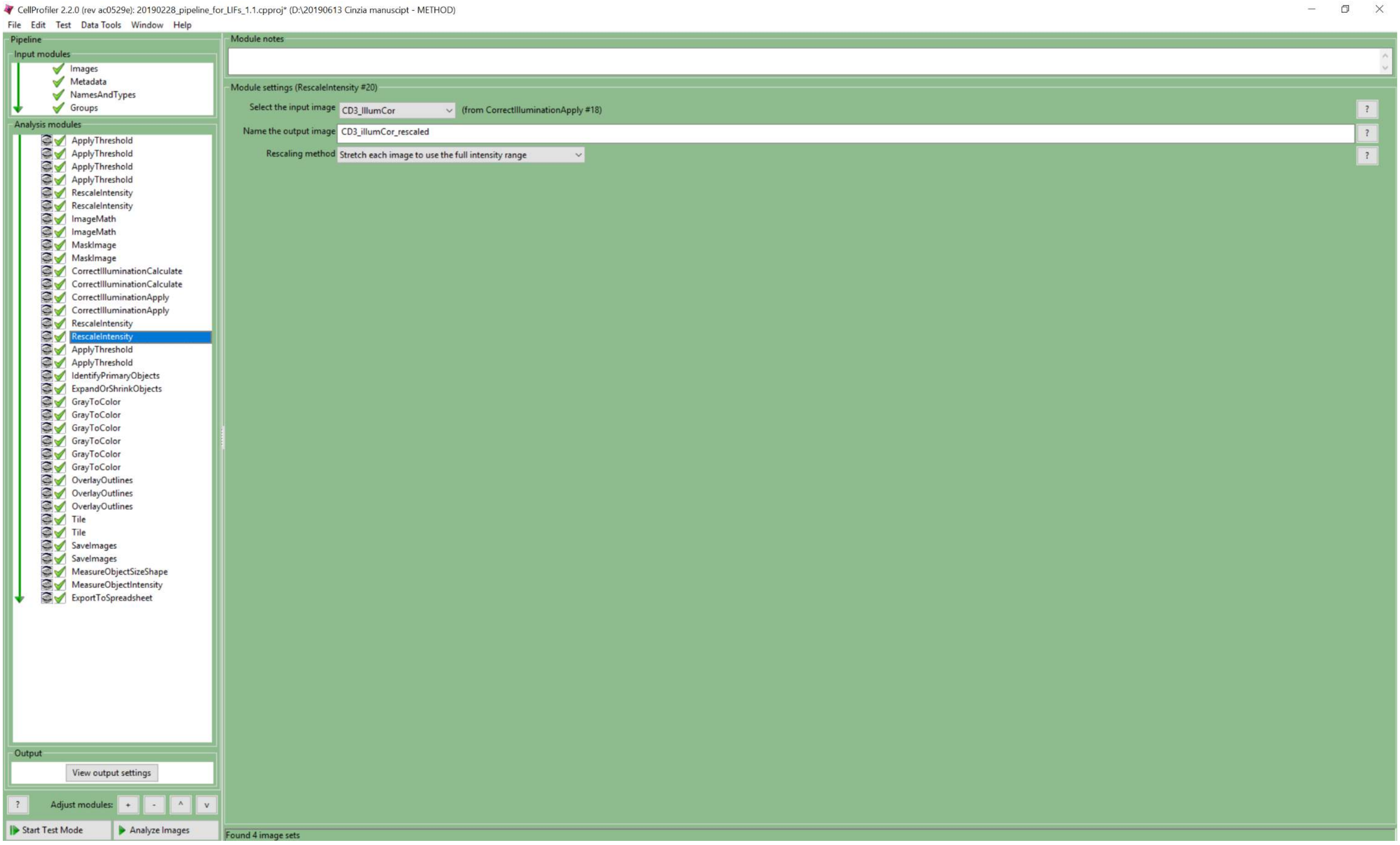
CellProfiler 2.2.0 (rev ac0529e): 20190228_pipeline_for_LIFs_1.1.cpproj* (D:\20190613 Cinzia manuscipt - METHOD)
File Edit Test Data Tools Window Help
Pipeline
Input modules
Images
Metadata
NamesAndTypes
Groups
Analysis modules
ApplyThreshold
ApplyThreshold
ApplyThreshold
ApplyThreshold
RescaleIntensity
RescaleIntensity
ImageMath
ImageMath
MaskImage
MaskImage
CorrectIlluminationCalculate
CorrectIlluminationCalculate
CorrectIlluminationApply
CorrectIlluminationApply
RescaleIntensity
RescaleIntensity
ApplyThreshold
ApplyThreshold
IdentifyPrimaryObjects
ExpandOrShrinkObjects
GrayToColor
GrayToColor
GrayToColor
GrayToColor
GrayToColor
GrayToColor
OverlayOutlines
OverlayOutlines
OverlayOutlines
Tile
Tile
SaveImages
SaveImages
MeasureObjectSizeShape
MeasureObjectIntensity
ExportToSpreadsheet
Output
View output settings
Adjust modules:
Start Test Mode
Analyze Images
Module notes
Module settings (RescaleIntensity #20)
Select the input image
CD3_IllumCor
(from CorrectIlluminationApply #18)
Name the output image
CD3_illumCor_rescaled
Rescaling method
Stretch each image to use the full intensity range
Found 4 image sets

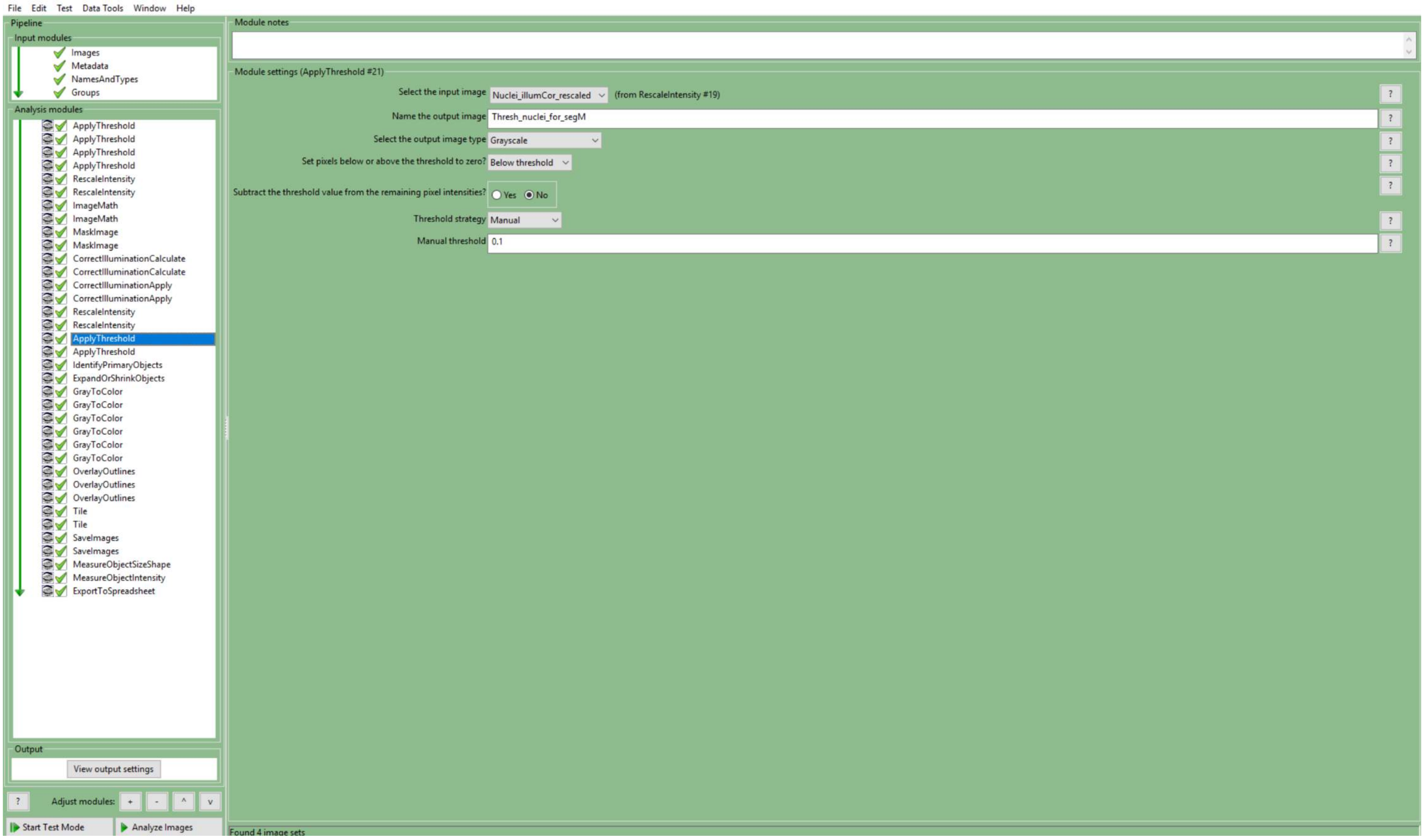
File Edit Test Data Tools Window Help
Pipeline
Input modules
Images
Metadata
NamesAndTypes
Groups
Analysis modules
ApplyThreshold
ApplyThreshold
ApplyThreshold
ApplyThreshold
RescaleIntensity
RescaleIntensity
ImageMath
ImageMath
MaskImage
MaskImage
CorrectIlluminationCalculate
CorrectIlluminationCalculate
CorrectIlluminationApply
CorrectIlluminationApply
RescaleIntensity
RescaleIntensity
ApplyThreshold
ApplyThreshold
IdentifyPrimaryObjects
ExpandOrShrinkObjects
GrayToColor
GrayToColor
GrayToColor
GrayToColor
GrayToColor
GrayToColor
OverlayOutlines
OverlayOutlines
OverlayOutlines
Tile
Tile
SaveImages
SaveImages
MeasureObjectSizeShape
MeasureObjectIntensity
ExportToSpreadsheet
Output
View output settings
Adjust modules:
Start Test Mode
Analyze Images
Module notes
Module settings (ApplyThreshold #21)
Select the input image Nuclei_illumCor_rescaled (from RescaleIntensity #19)
Name the output image Thresh_nuclei_for_segM
Select the output image type Grayscale
Set pixels below or above the threshold to zero? Below threshold
Subtract the threshold value from the remaining pixel intensities? Yes No
Threshold strategy Manual
Manual threshold 0.1
Found 4 image sets

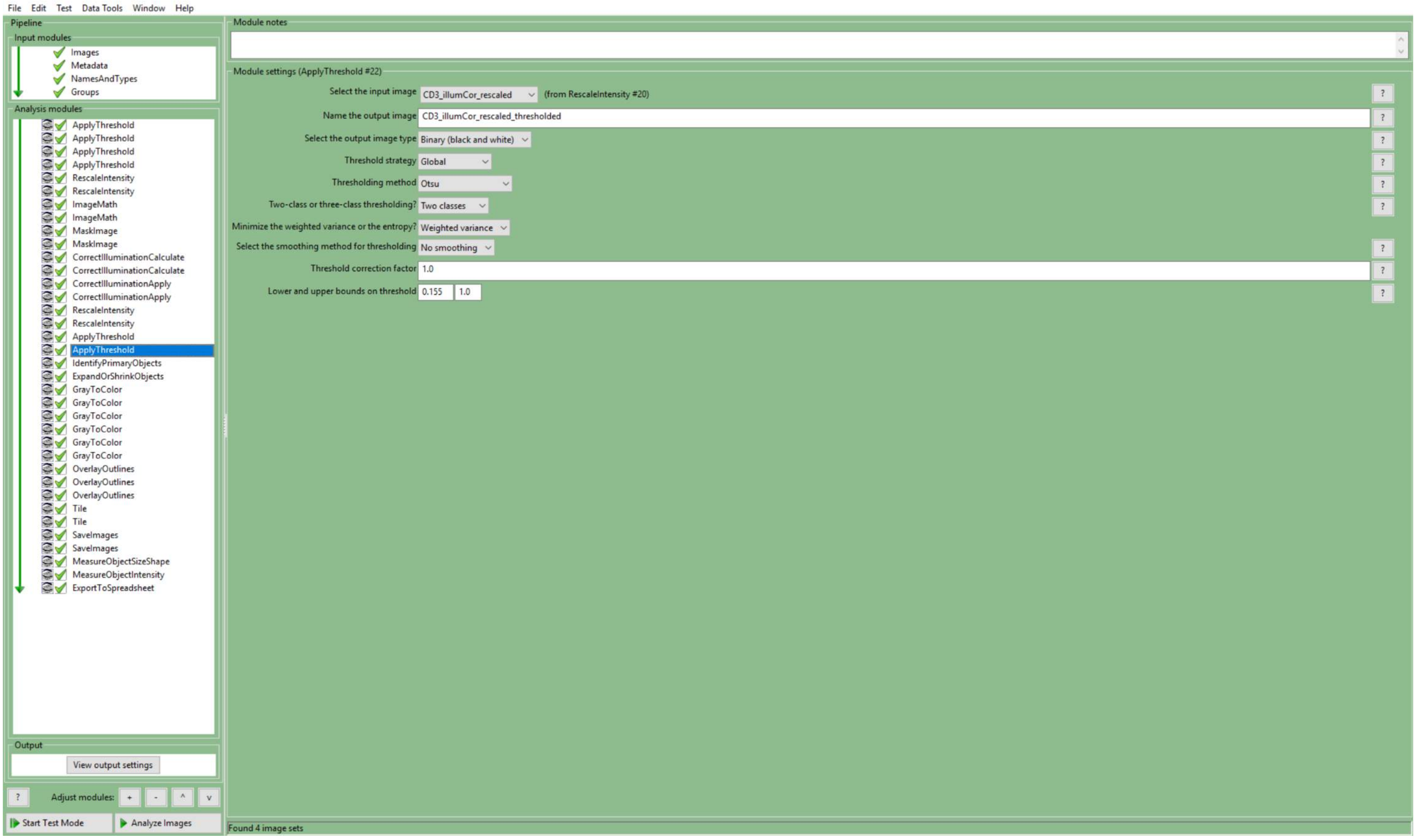
File Edit Test Data Tools Window Help
Pipeline
Input modules
Images
Metadata
NamesAndTypes
Groups
Analysis modules
ApplyThreshold
ApplyThreshold
ApplyThreshold
ApplyThreshold
RescaleIntensity
RescaleIntensity
ImageMath
ImageMath
MaskImage
MaskImage
CorrectIlluminationCalculate
CorrectIlluminationCalculate
CorrectIlluminationApply
CorrectIlluminationApply
RescaleIntensity
RescaleIntensity
ApplyThreshold
ApplyThreshold
IdentifyPrimaryObjects
ExpandOrShrinkObjects
GrayToColor
GrayToColor
GrayToColor
GrayToColor
GrayToColor
GrayToColor
OverlayOutlines
OverlayOutlines
OverlayOutlines
Tile
Tile
SaveImages
SaveImages
MeasureObjectSizeShape
MeasureObjectIntensity
ExportToSpreadsheet
Output
View output settings
Adjust modules:
Start Test Mode
Analyze Images
Module notes
Module settings (ApplyThreshold #22)
Select the input image
CD3_illumCor_rescaled
(from RescaleIntensity #20)
Name the output image
CD3_illumCor_rescaled_thresholded
Select the output image type
Binary (black and white)
Threshold strategy
Global
Thresholding method
Otsu
Two-class or three-class thresholding?
Two classes
Minimize the weighted variance or the entropy?
Weighted variance
Select the smoothing method for thresholding
No smoothing
Threshold correction factor
1.0
Lower and upper bounds on threshold
0.155
1.0
Found 4 image sets

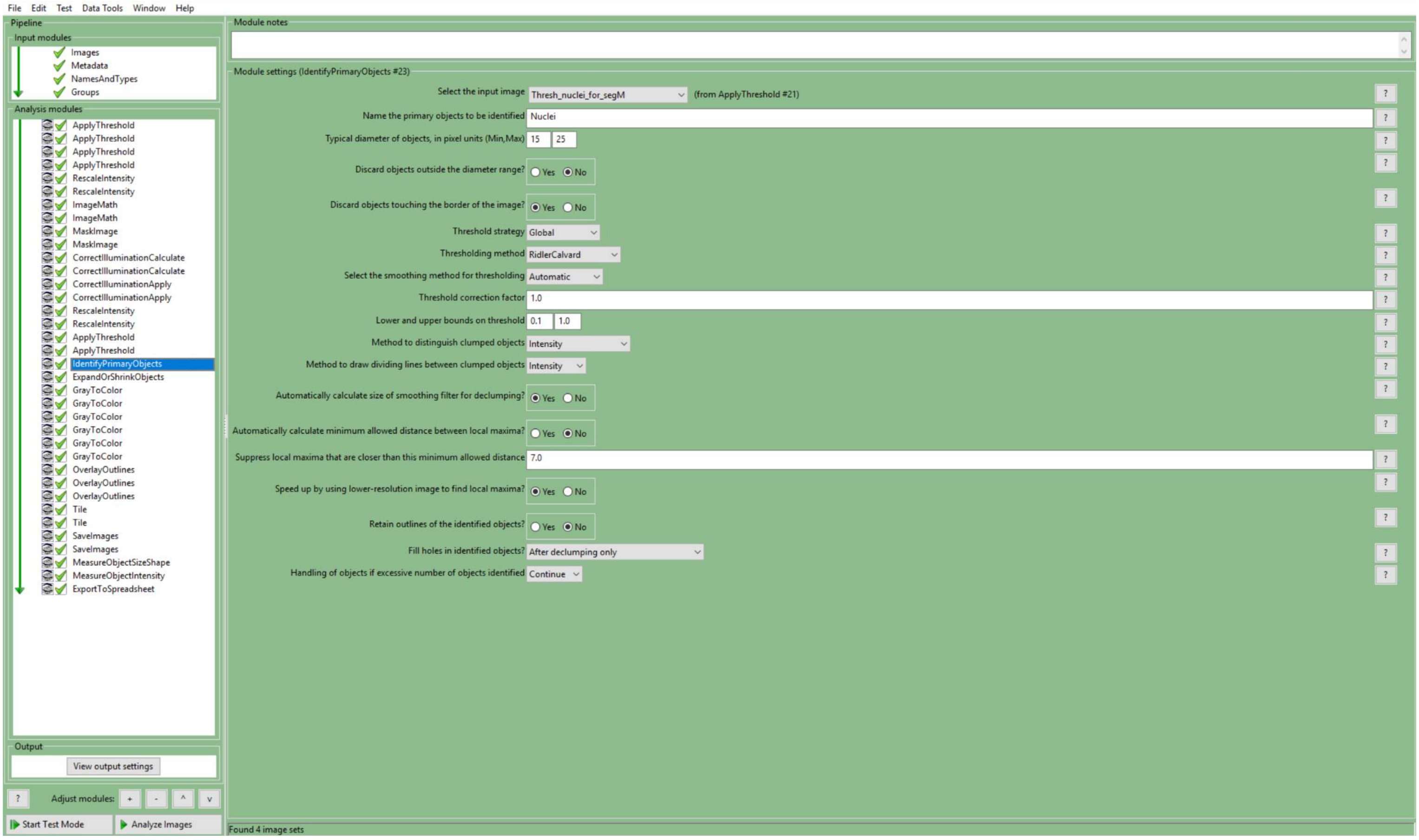
File Edit Test Data Tools Window Help
Pipeline
Input modules
Images
Metadata
NamesAndTypes
Groups
Analysis modules
ApplyThreshold
ApplyThreshold
ApplyThreshold
ApplyThreshold
RescaleIntensity
RescaleIntensity
ImageMath
ImageMath
MaskImage
MaskImage
CorrectIlluminationCalculate
CorrectIlluminationCalculate
CorrectIlluminationApply
CorrectIlluminationApply
RescaleIntensity
RescaleIntensity
ApplyThreshold
ApplyThreshold
IdentifyPrimaryObjects
ExpandOrShrinkObjects
GrayToColor
GrayToColor
GrayToColor
GrayToColor
GrayToColor
GrayToColor
OverlayOutlines
OverlayOutlines
OverlayOutlines
Tile
Tile
SaveImages
SaveImages
MeasureObjectSizeShape
MeasureObjectIntensity
ExportToSpreadsheet
Output
View output settings
Adjust modules:
Start Test Mode
Analyze Images
Module notes
Module settings (IdentifyPrimaryObjects #23)
Select the input image Thresh_nuclei_for_segM (from ApplyThreshold #21)
Name the primary objects to be identified Nuclei
Typical diameter of objects, in pixel units (Min,Max) 15 25
Discard objects outside the diameter range? Yes No
Discard objects touching the border of the image? Yes No
Threshold strategy Global
Thresholding method RidlerCalvard
Select the smoothing method for thresholding Automatic
Threshold correction factor 1.0
Lower and upper bounds on threshold 0.1 1.0
Method to distinguish clumped objects Intensity
Method to draw dividing lines between clumped objects Intensity
Automatically calculate size of smoothing filter for declumping? Yes No
Automatically calculate minimum allowed distance between local maxima? Yes No
Suppress local maxima that are closer than this minimum allowed distance 7.0
Speed up by using lower-resolution image to find local maxima? Yes No
Retain outlines of the identified objects? Yes No
Fill holes in identified objects? After declumping only
Handling of objects if excessive number of objects identified Continue
Found 4 image sets

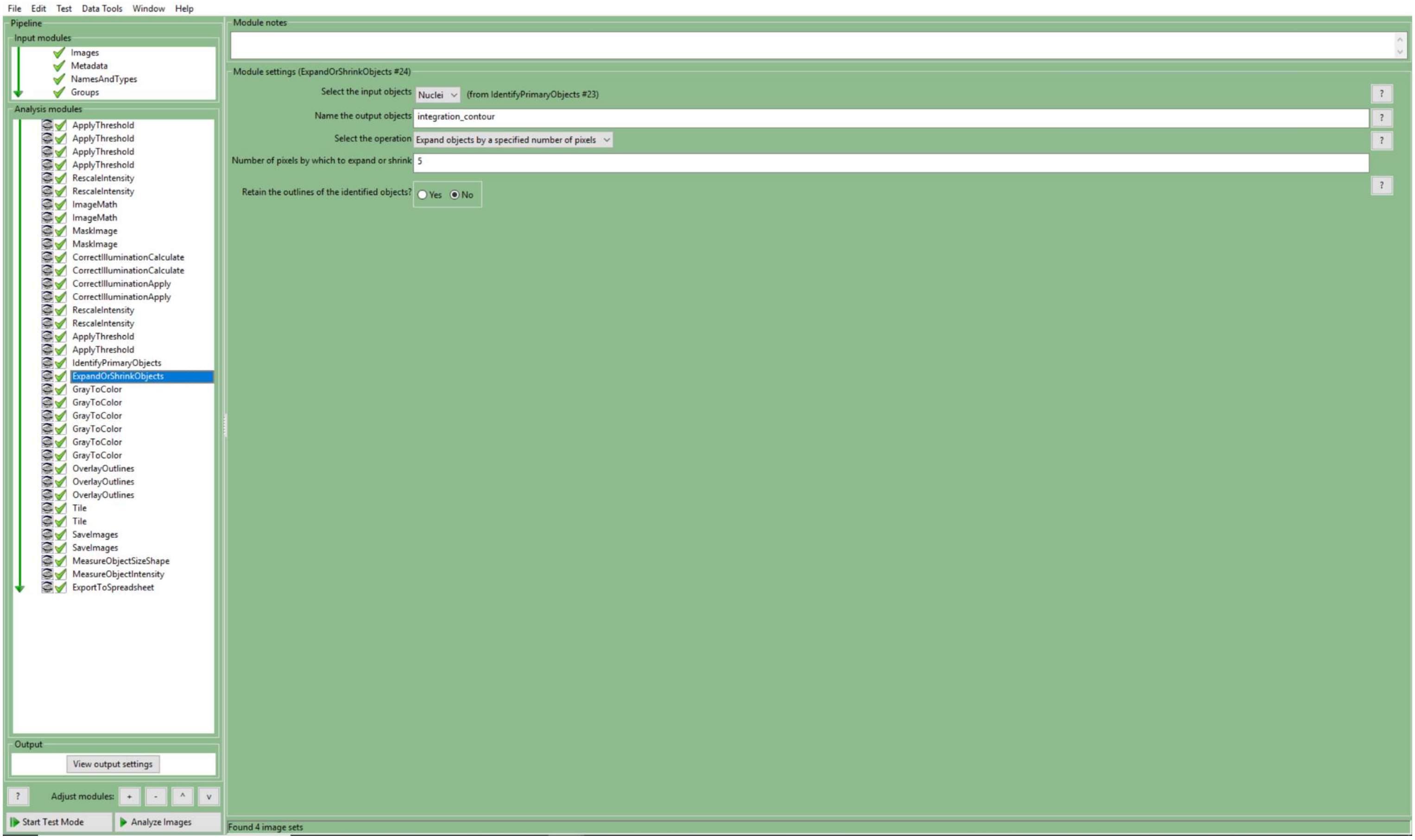

File Edit Test Data Tools Window Help
Pipeline
Input modules
Images
Metadata
NamesAndTypes
Groups
Analysis modules
ApplyThreshold
ApplyThreshold
ApplyThreshold
ApplyThreshold
RescaleIntensity
RescaleIntensity
ImageMath
ImageMath
MaskImage
MaskImage
CorrectIlluminationCalculate
CorrectIlluminationCalculate
CorrectIlluminationApply
CorrectIlluminationApply
RescaleIntensity
RescaleIntensity
ApplyThreshold
ApplyThreshold
IdentifyPrimaryObjects
ExpandOrShrinkObjects
GrayToColor
GrayToColor
GrayToColor
GrayToColor
GrayToColor
GrayToColor
OverlayOutlines
OverlayOutlines
OverlayOutlines
Tile
Tile
SaveImages
SaveImages
MeasureObjectSizeShape
MeasureObjectIntensity
ExportToSpreadsheet
Output
View output settings
Adjust modules:
Start Test Mode
Analyze Images
Module notes
Module settings (ExpandOrShrinkObjects #24)
Select the input objects
Nuclei
(from IdentifyPrimaryObjects #23)
Name the output objects
integration_contour
Select the operation
Expand objects by a specified number of pixels
Number of pixels by which to expand or shrink
5
Retain the outlines of the identified objects?
Yes
No
Found 4 image sets

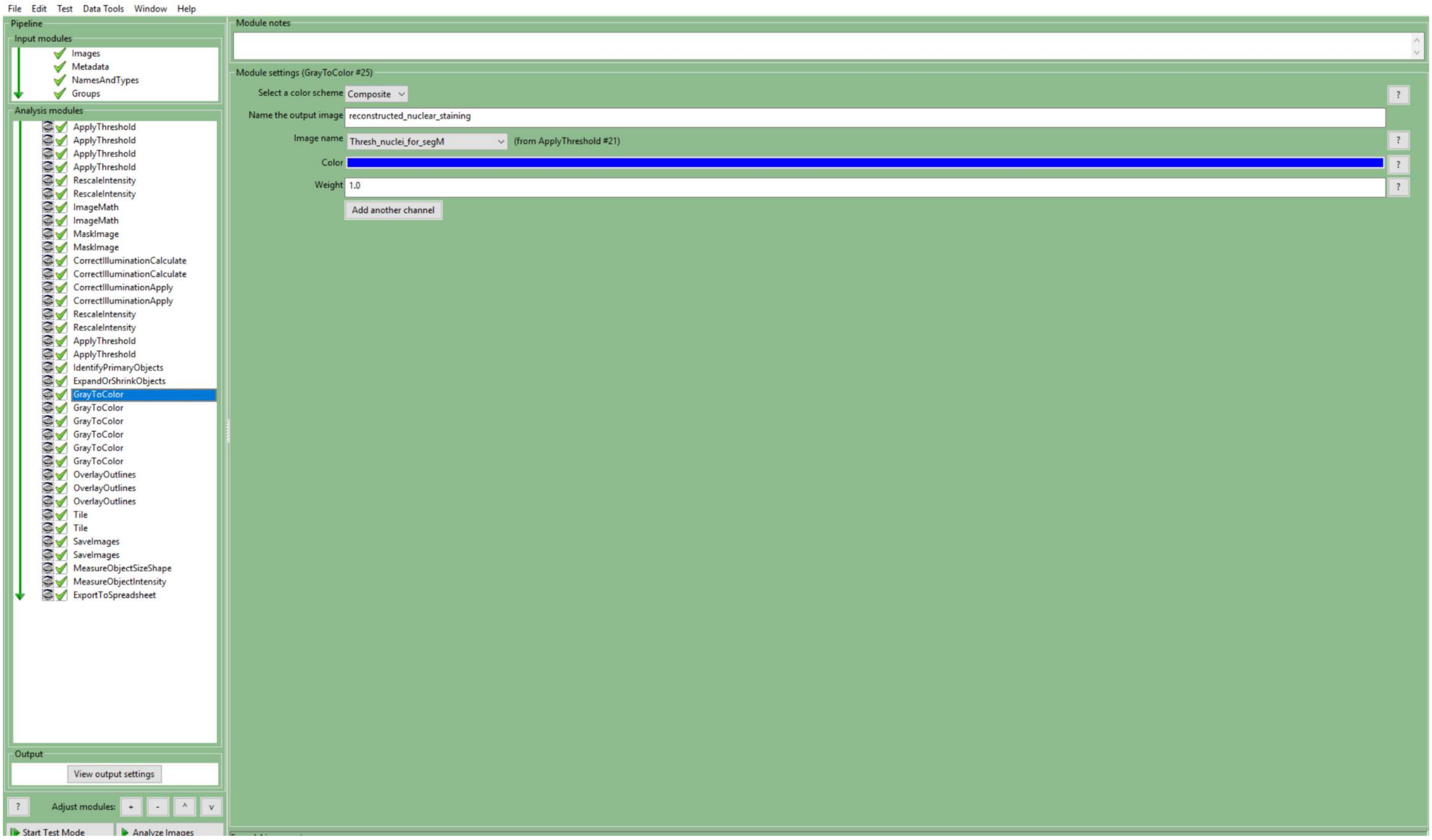

File Edit Test Data Tools Window Help
Pipeline
Input modules
Images
Metadata
NamesAndTypes
Groups
Analysis modules
ApplyThreshold
ApplyThreshold
ApplyThreshold
ApplyThreshold
RescaleIntensity
RescaleIntensity
ImageMath
ImageMath
MaskImage
MaskImage
CorrectIlluminationCalculate
CorrectIlluminationCalculate
CorrectIlluminationApply
CorrectIlluminationApply
RescaleIntensity
RescaleIntensity
ApplyThreshold
ApplyThreshold
IdentifyPrimaryObjects
ExpandOrShrinkObjects
GrayToColor
GrayToColor
GrayToColor
GrayToColor
GrayToColor
GrayToColor
OverlayOutlines
OverlayOutlines
OverlayOutlines
Tile
Tile
SaveImages
SaveImages
MeasureObjectSizeShape
MeasureObjectIntensity
ExportToSpreadsheet
Output
View output settings
Adjust modules:
Start Test Mode
Analyze Images
Module notes
Module settings (GrayToColor #25)
Select a color scheme Composite
Name the output image reconstructed_nuclear_staining
Image name Thresh_nuclei_for_segM (from ApplyThreshold #21)
Color
Weight 1.0
Add another channel

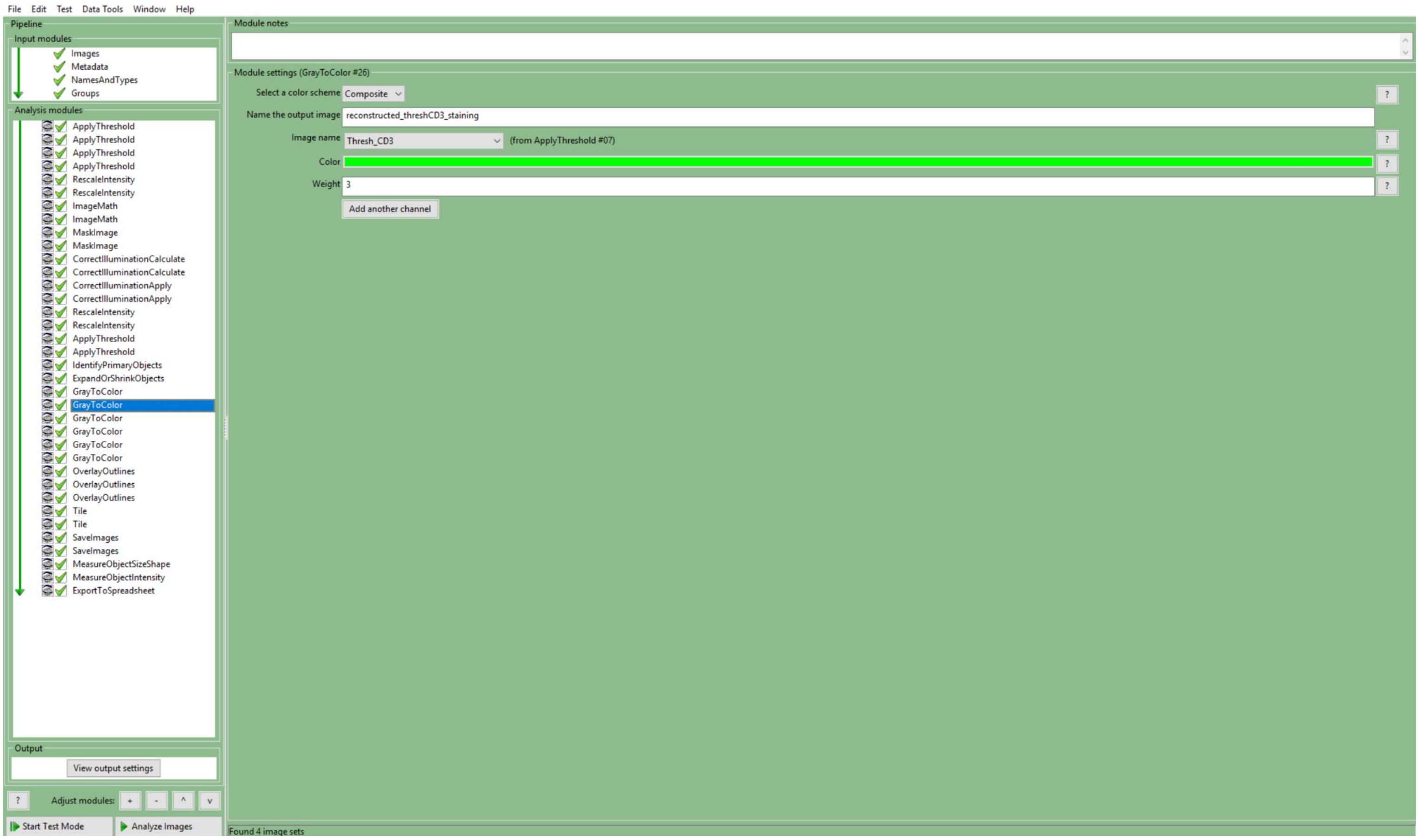
File Edit Test Data Tools Window Help
Pipeline
Input modules
Images
Metadata
NamesAndTypes
Groups
Analysis modules
ApplyThreshold
ApplyThreshold
ApplyThreshold
ApplyThreshold
RescaleIntensity
RescaleIntensity
ImageMath
ImageMath
MaskImage
MaskImage
CorrectIlluminationCalculate
CorrectIlluminationCalculate
CorrectIlluminationApply
CorrectIlluminationApply
RescaleIntensity
RescaleIntensity
ApplyThreshold
ApplyThreshold
IdentifyPrimaryObjects
ExpandOrShrinkObjects
GrayToColor
GrayToColor
GrayToColor
GrayToColor
GrayToColor
GrayToColor
OverlayOutlines
OverlayOutlines
OverlayOutlines
Tile
Tile
SaveImages
SaveImages
MeasureObjectSizeShape
MeasureObjectIntensity
ExportToSpreadsheet
Output
View output settings
Adjust modules:
Start Test Mode
Analyze Images
Module notes
Module settings (GrayToColor #26)
Select a color scheme
Composite
Name the output image
reconstructed_threshCD3_staining
Image name
Thresh_CD3
(from ApplyThreshold #07)
Color
Weight
3
Add another channel
Found 4 image sets

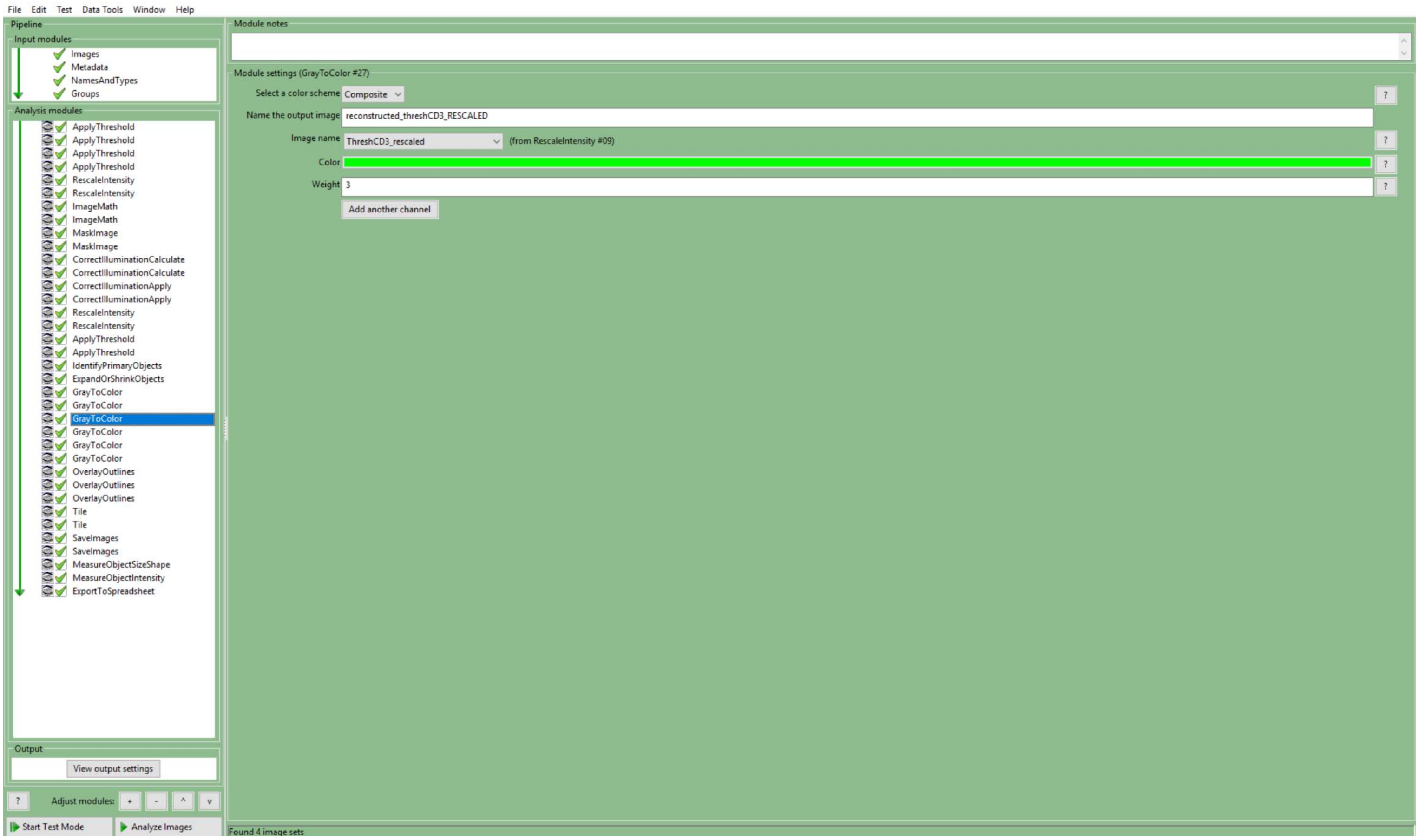
File Edit Test Data Tools Window Help
Pipeline
Input modules
Images
Metadata
NamesAndTypes
Groups
Analysis modules
ApplyThreshold
ApplyThreshold
ApplyThreshold
ApplyThreshold
RescaleIntensity
RescaleIntensity
ImageMath
ImageMath
MaskImage
MaskImage
CorrectIlluminationCalculate
CorrectIlluminationCalculate
CorrectIlluminationApply
CorrectIlluminationApply
RescaleIntensity
RescaleIntensity
ApplyThreshold
ApplyThreshold
IdentifyPrimaryObjects
ExpandOrShrinkObjects
GrayToColor
GrayToColor
GrayToColor
GrayToColor
GrayToColor
GrayToColor
OverlayOutlines
OverlayOutlines
OverlayOutlines
Tile
Tile
SaveImages
SaveImages
MeasureObjectSizeShape
MeasureObjectIntensity
ExportToSpreadsheet
Output
View output settings
Adjust modules:
Start Test Mode
Analyze Images
Module notes
Module settings (GrayToColor #27)
Select a color scheme Composite
Name the output image reconstructed_threshCD3_RESCALED
Image name ThreshCD3_rescaled (from RescaleIntensity #09)
Color
Weight 3
Add another channel
Found 4 image sets

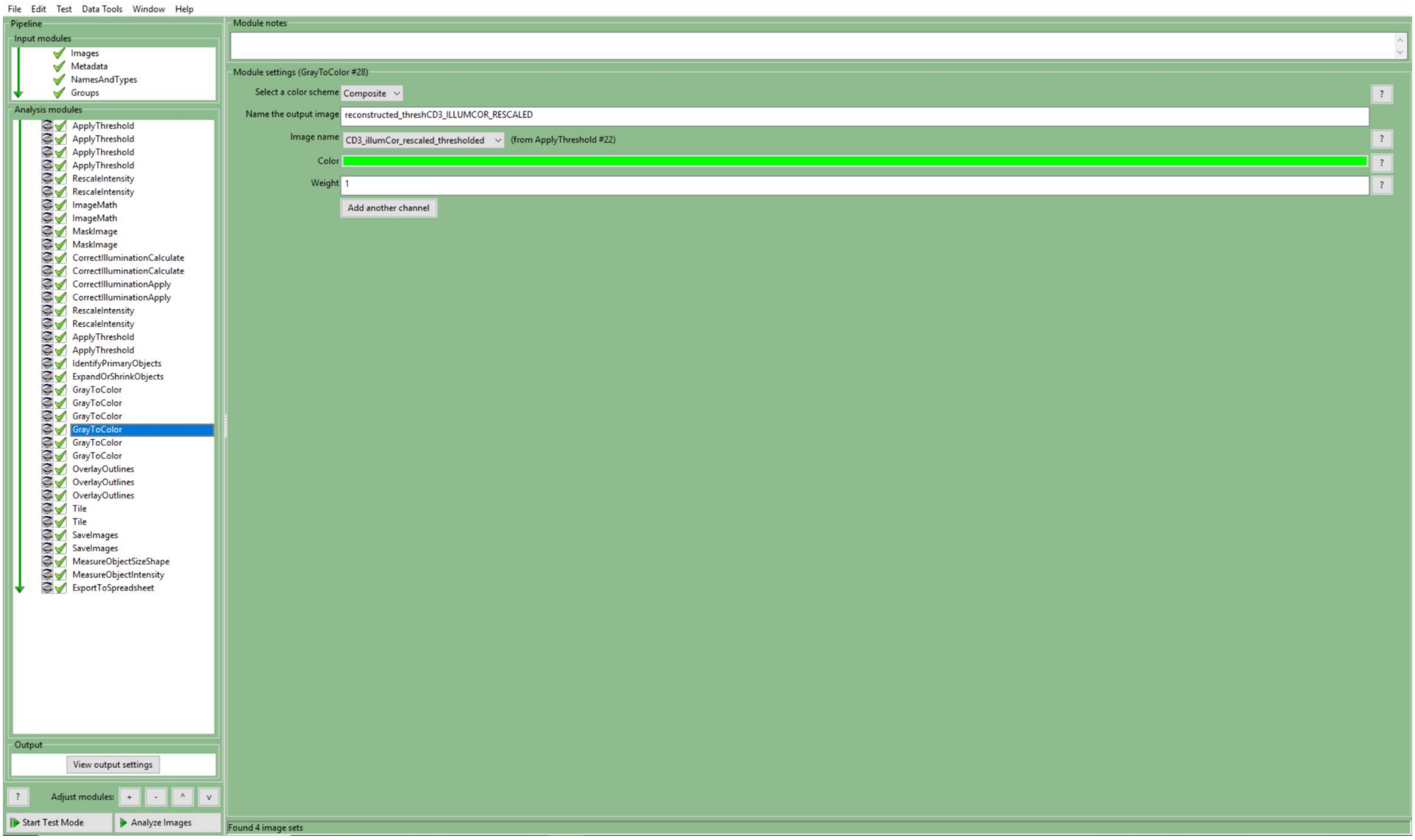
File Edit Test Data Tools Window Help
Pipeline
Input modules
Images
Metadata
NamesAndTypes
Groups
Analysis modules
ApplyThreshold
ApplyThreshold
ApplyThreshold
ApplyThreshold
RescaleIntensity
RescaleIntensity
ImageMath
ImageMath
MaskImage
MaskImage
CorrectIlluminationCalculate
CorrectIlluminationCalculate
CorrectIlluminationApply
CorrectIlluminationApply
RescaleIntensity
RescaleIntensity
ApplyThreshold
ApplyThreshold
IdentifyPrimaryObjects
ExpandOrShrinkObjects
GrayToColor
GrayToColor
GrayToColor
GrayToColor
GrayToColor
GrayToColor
OverlayOutlines
OverlayOutlines
OverlayOutlines
Tile
Tile
SaveImages
SaveImages
MeasureObjectSizeShape
MeasureObjectIntensity
ExportToSpreadsheet
Output
View output settings
Adjust modules:
Start Test Mode
Analyze Images
Module notes
Module settings (GrayToColor #28)
Select a color scheme
Composite
Name the output image
reconstructed_threshCD3_ILLUMCOR_RESCALED
Image name
CD3_illumCor_rescaled_thresholded
(from ApplyThreshold #22)
Color
Weight
1
Add another channel
Found 4 image sets

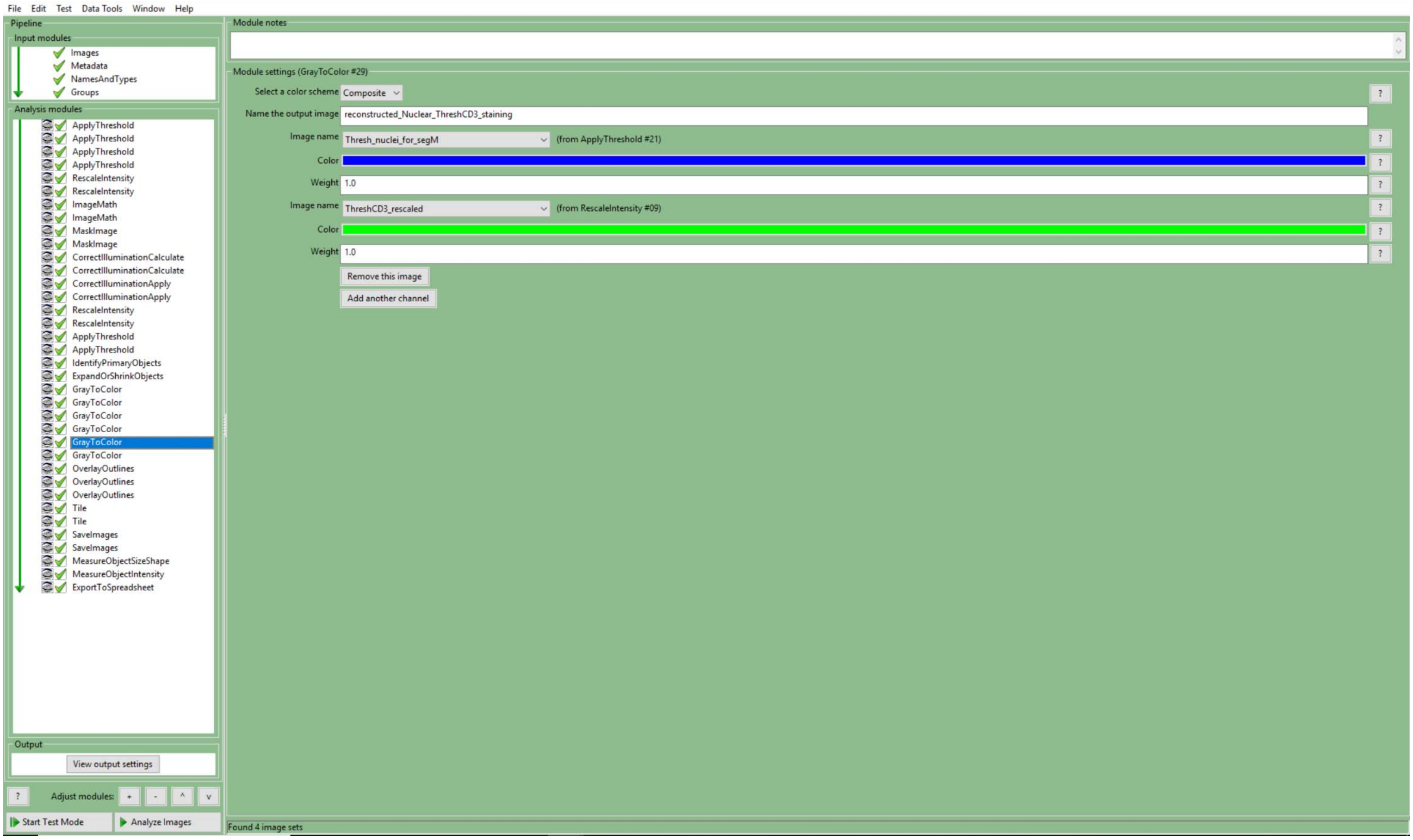
File Edit Test Data Tools Window Help
Pipeline
Input modules
Images
Metadata
NamesAndTypes
Groups
Analysis modules
ApplyThreshold
ApplyThreshold
ApplyThreshold
ApplyThreshold
RescaleIntensity
RescaleIntensity
ImageMath
ImageMath
MaskImage
MaskImage
CorrectIlluminationCalculate
CorrectIlluminationCalculate
CorrectIlluminationApply
CorrectIlluminationApply
RescaleIntensity
RescaleIntensity
ApplyThreshold
ApplyThreshold
IdentifyPrimaryObjects
ExpandOrShrinkObjects
GrayToColor
GrayToColor
GrayToColor
GrayToColor
GrayToColor
GrayToColor
OverlayOutlines
OverlayOutlines
OverlayOutlines
Tile
Tile
SaveImages
SaveImages
MeasureObjectSizeShape
MeasureObjectIntensity
ExportToSpreadsheet
Output
View output settings
Adjust modules:
Start Test Mode
Analyze Images
Module notes
Module settings (GrayToColor #29)
Select a color scheme
Composite
Name the output image
reconstructed_Nuclear_ThreshCD3_staining
Image name
Thresh_nuclei_for_segM
(from ApplyThreshold #21)
Color
Weight
1.0
Image name
ThreshCD3_rescaled
(from RescaleIntensity #09)
Color
Weight
1.0
Remove this image
Add another channel
Found 4 image sets

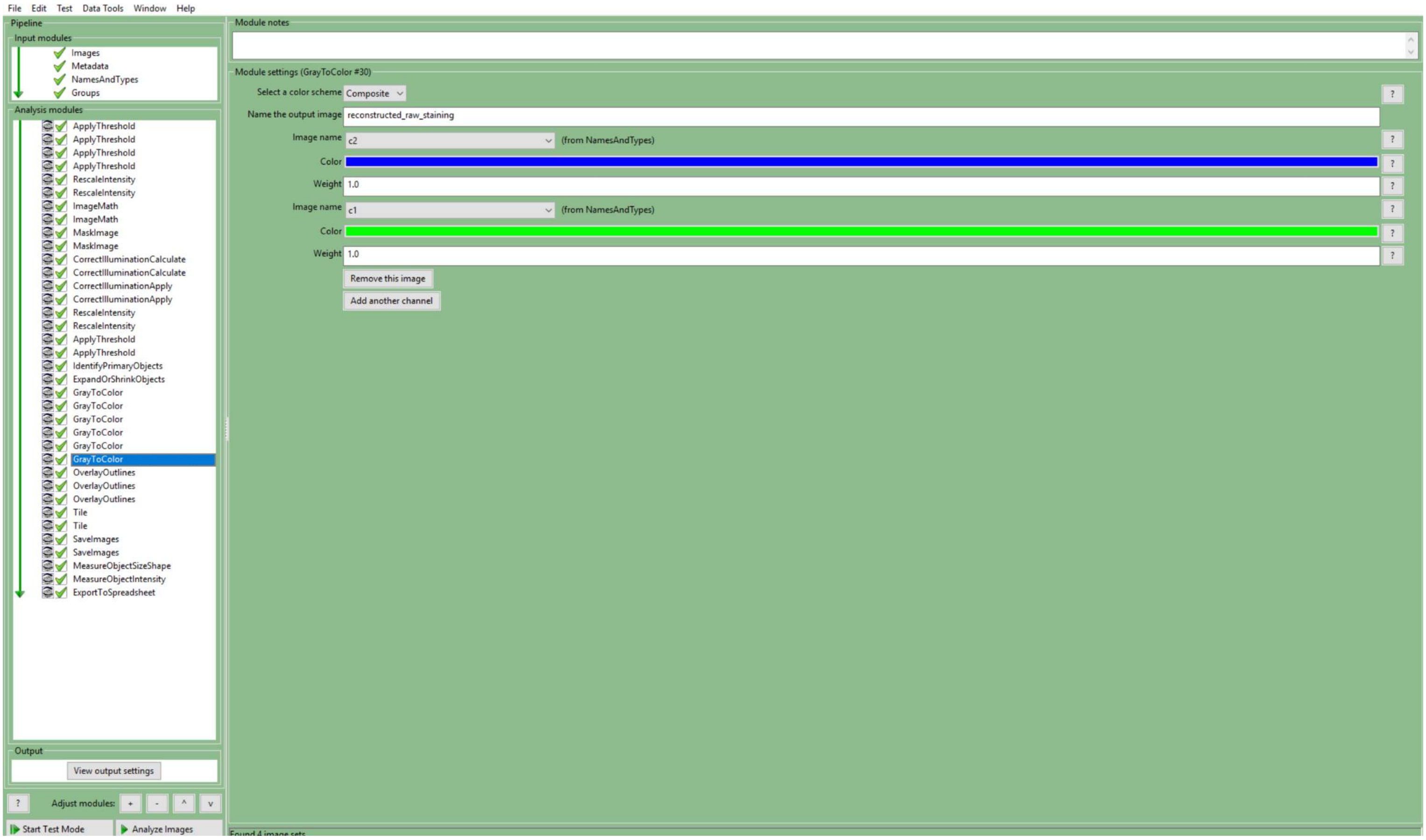

File Edit Test Data Tools Window Help
Pipeline
Input modules
Images
Metadata
NamesAndTypes
Groups
Analysis modules
ApplyThreshold
ApplyThreshold
ApplyThreshold
ApplyThreshold
RescaleIntensity
RescaleIntensity
ImageMath
ImageMath
MaskImage
MaskImage
CorrectIlluminationCalculate
CorrectIlluminationCalculate
CorrectIlluminationApply
CorrectIlluminationApply
RescaleIntensity
RescaleIntensity
ApplyThreshold
ApplyThreshold
IdentifyPrimaryObjects
ExpandOrShrinkObjects
GrayToColor
GrayToColor
GrayToColor
GrayToColor
GrayToColor
GrayToColor
OverlayOutlines
OverlayOutlines
OverlayOutlines
Tile
Tile
SaveImages
SaveImages
MeasureObjectSizeShape
MeasureObjectIntensity
ExportToSpreadsheet
Output
View output settings
Adjust modules:
Start Test Mode
Analyze Images
Module notes
Module settings (GrayToColor #30)
Select a color scheme
Composite
Name the output image
reconstructed_raw_staining
Image name
c2
(from NamesAndTypes)
Color
Weight
1.0
Image name
c1
(from NamesAndTypes)
Color
Weight
1.0
Remove this image
Add another channel

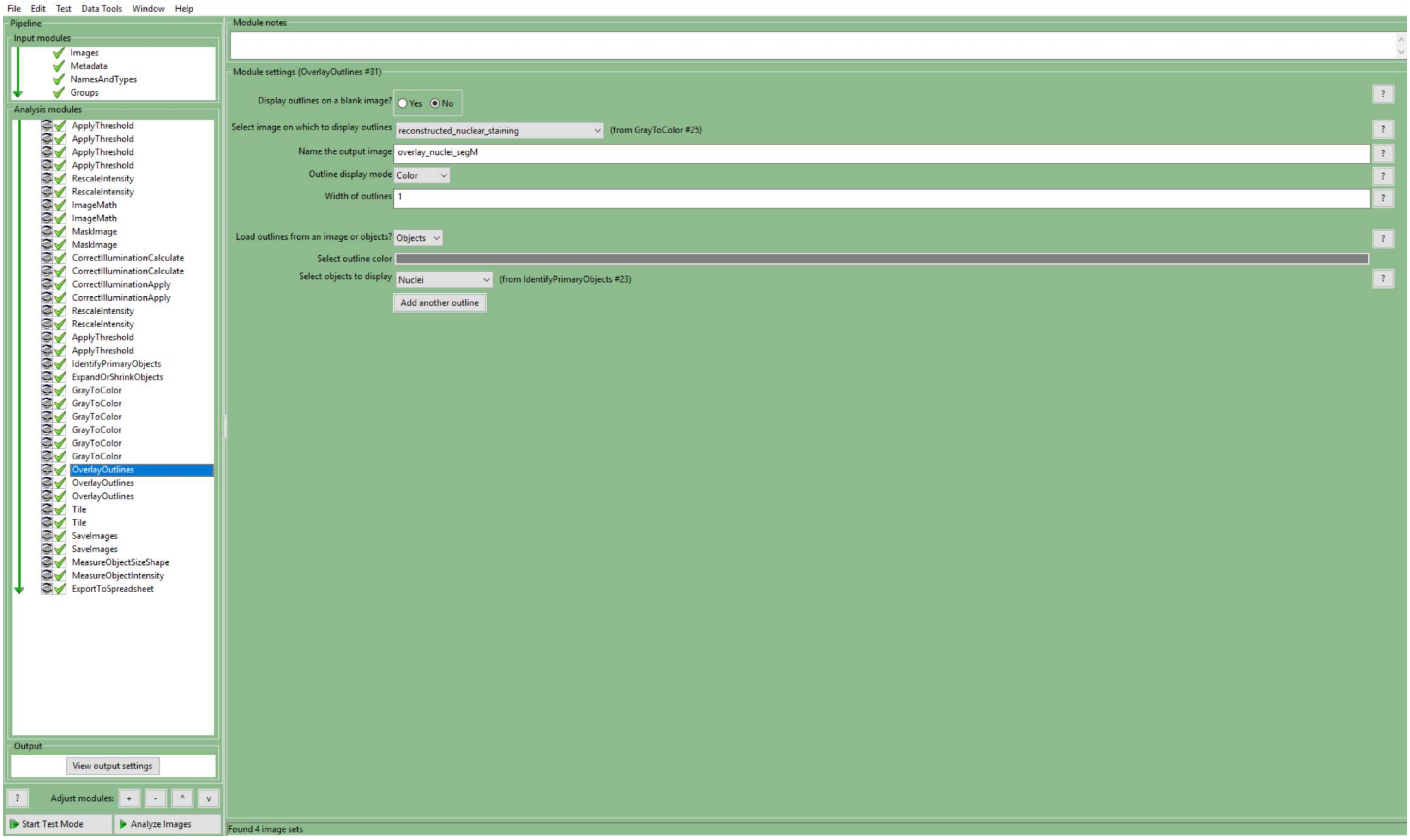
File Edit Test Data Tools Window Help
Pipeline
Input modules
Images
Metadata
NamesAndTypes
Groups
Analysis modules
ApplyThreshold
ApplyThreshold
ApplyThreshold
ApplyThreshold
RescaleIntensity
RescaleIntensity
ImageMath
ImageMath
MaskImage
MaskImage
CorrectIlluminationCalculate
CorrectIlluminationCalculate
CorrectIlluminationApply
CorrectIlluminationApply
RescaleIntensity
RescaleIntensity
ApplyThreshold
ApplyThreshold
IdentifyPrimaryObjects
ExpandOrShrinkObjects
GrayToColor
GrayToColor
GrayToColor
GrayToColor
GrayToColor
GrayToColor
OverlayOutlines
OverlayOutlines
OverlayOutlines
Tile
Tile
SaveImages
SaveImages
MeasureObjectSizeShape
MeasureObjectIntensity
ExportToSpreadsheet
Output
View output settings
Adjust modules:
Start Test Mode
Analyze Images
Module notes
Module settings (OverlayOutlines #31)
Display outlines on a blank image?
Yes
No
Select image on which to display outlines
reconstructed_nuclear_staining
(from GrayToColor #25)
Name the output image
overlay_nuclei_segM
Outline display mode
Color
Width of outlines
1
Load outlines from an image or objects?
Objects
Select outline color
Select objects to display
Nuclei
(from IdentifyPrimaryObjects #23)
Add another outline
Found 4 image sets

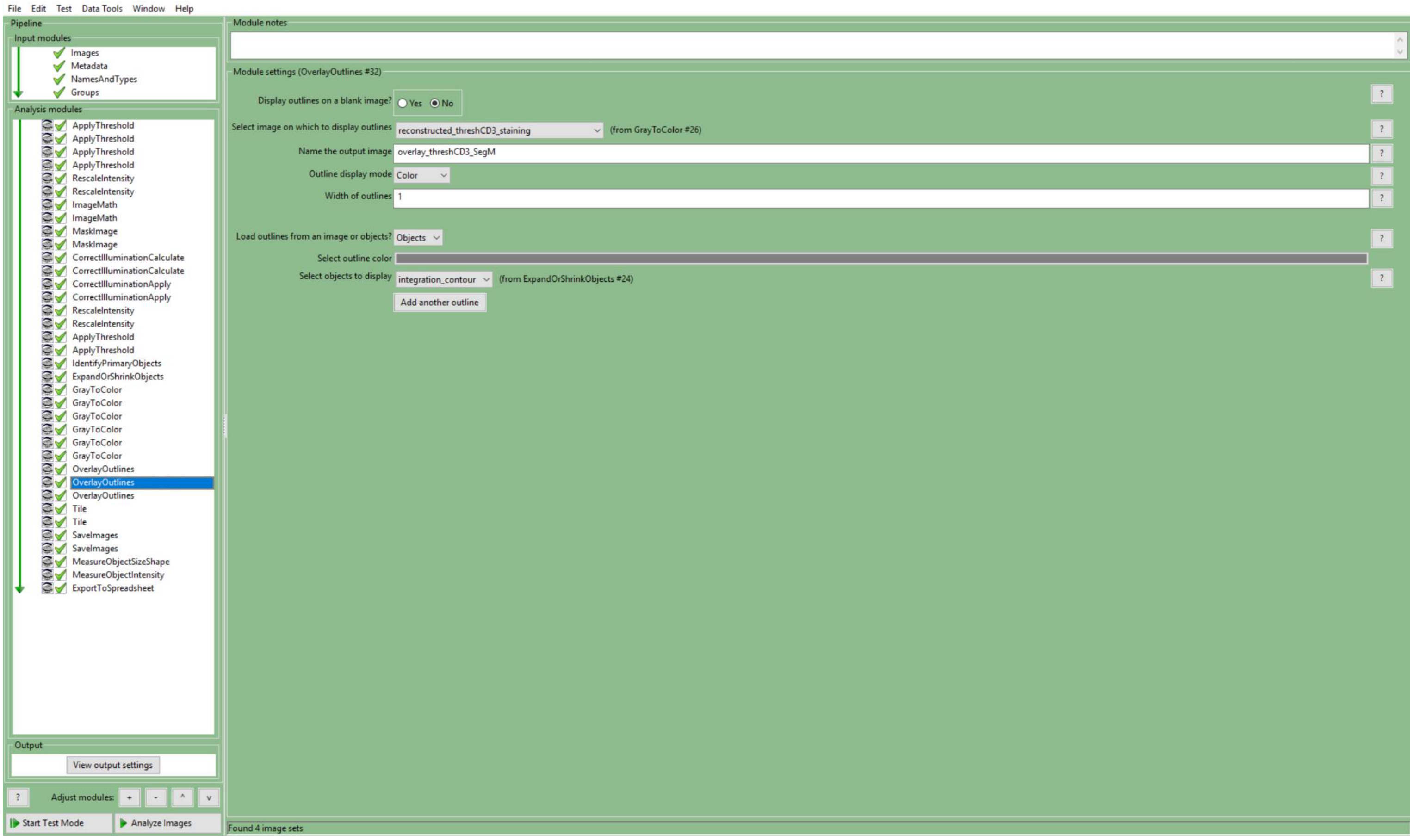
File Edit Test Data Tools Window Help
Pipeline
Input modules
Images
Metadata
NamesAndTypes
Groups
Analysis modules
ApplyThreshold
ApplyThreshold
ApplyThreshold
ApplyThreshold
RescaleIntensity
RescaleIntensity
ImageMath
ImageMath
MaskImage
MaskImage
CorrectIlluminationCalculate
CorrectIlluminationCalculate
CorrectIlluminationApply
CorrectIlluminationApply
RescaleIntensity
RescaleIntensity
ApplyThreshold
ApplyThreshold
IdentifyPrimaryObjects
ExpandOrShrinkObjects
GrayToColor
GrayToColor
GrayToColor
GrayToColor
GrayToColor
GrayToColor
OverlayOutlines
OverlayOutlines
OverlayOutlines
Tile
Tile
SaveImages
SaveImages
MeasureObjectSizeShape
MeasureObjectIntensity
ExportToSpreadsheet
Output
View output settings
Adjust modules:
Start Test Mode
Analyze Images
Module notes
Module settings (OverlayOutlines #32)
Display outlines on a blank image? Yes No
Select image on which to display outlines reconstructed_threshCD3_staining (from GrayToColor #26)
Name the output image overlay_threshCD3_SegM
Outline display mode Color
Width of outlines 1
Load outlines from an image or objects? Objects
Select outline color
Select objects to display integration_contour (from ExpandOrShrinkObjects #24)
Add another outline
Found 4 image sets

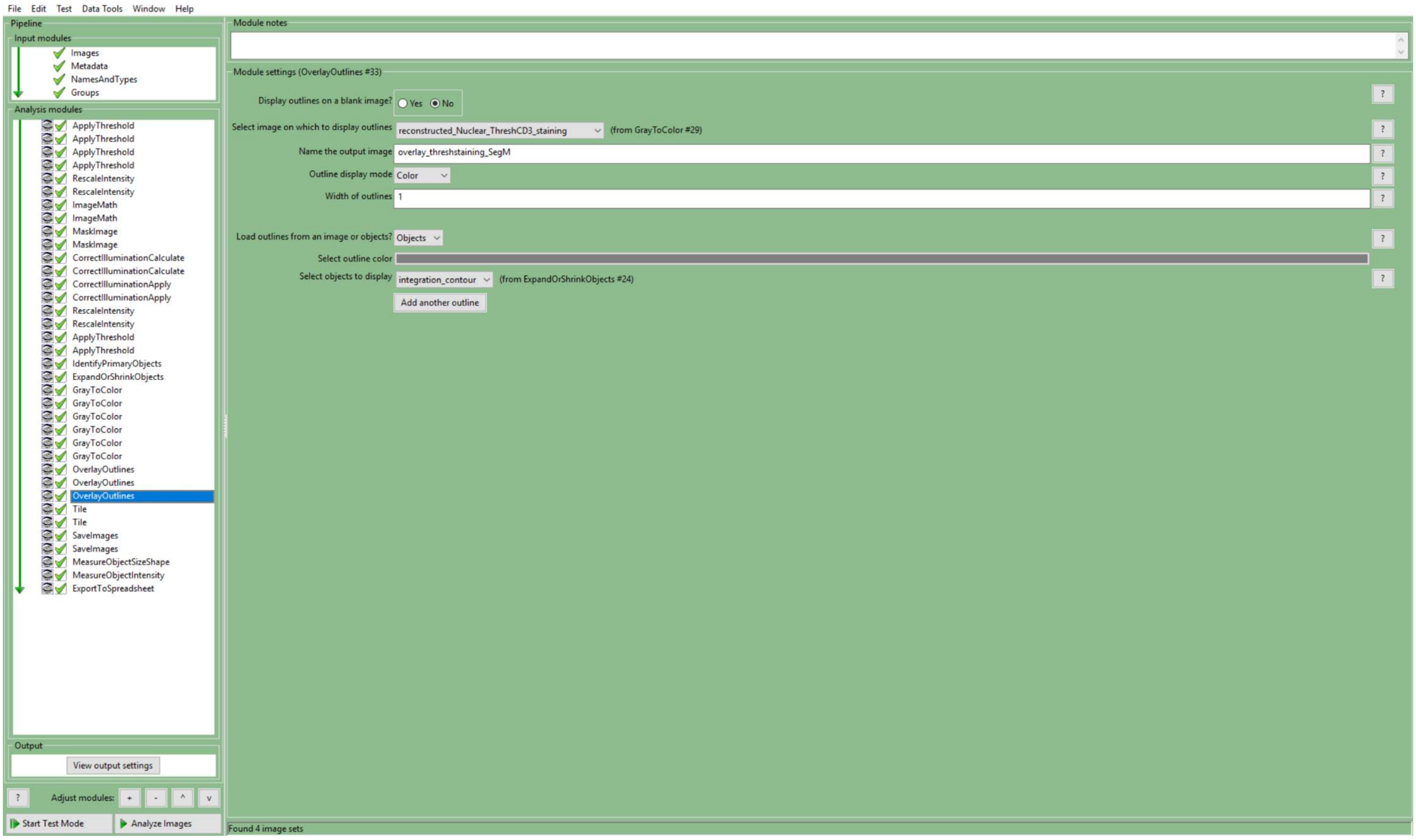
File Edit Test Data Tools Window Help
Pipeline
Input modules
Images
Metadata
NamesAndTypes
Groups
Analysis modules
ApplyThreshold
ApplyThreshold
ApplyThreshold
ApplyThreshold
RescaleIntensity
RescaleIntensity
ImageMath
ImageMath
MaskImage
MaskImage
CorrectIlluminationCalculate
CorrectIlluminationCalculate
CorrectIlluminationApply
CorrectIlluminationApply
RescaleIntensity
RescaleIntensity
ApplyThreshold
ApplyThreshold
IdentifyPrimaryObjects
ExpandOrShrinkObjects
GrayToColor
GrayToColor
GrayToColor
GrayToColor
GrayToColor
GrayToColor
OverlayOutlines
OverlayOutlines
OverlayOutlines
Tile
Tile
SaveImages
SaveImages
MeasureObjectSizeShape
MeasureObjectIntensity
ExportToSpreadsheet
Output
View output settings
Adjust modules: + - ^ v
Start Test Mode
Analyze Images
Module notes
Module settings (OverlayOutlines #33)
Display outlines on a blank image? Yes No
Select image on which to display outlines reconstructed_Nuclear_ThreshCD3_staining (from GrayToColor #29)
Name the output image overlay_threshstaining_SegM
Outline display mode Color
Width of outlines 1
Load outlines from an image or objects? Objects
Select outline color
Select objects to display integration_contour (from ExpandOrShrinkObjects #24)
Add another outline
Found 4 image sets

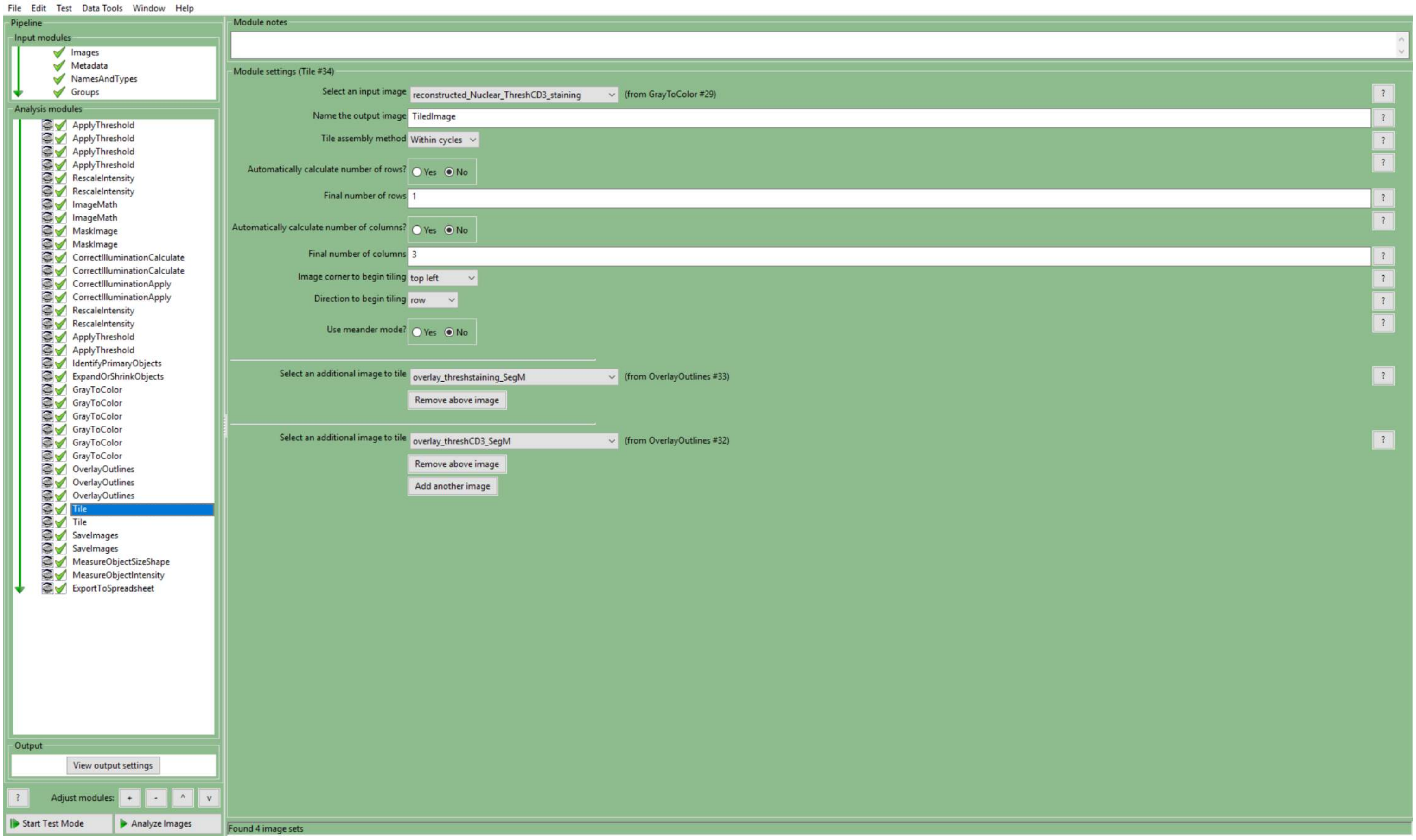
File Edit Test Data Tools Window Help
Pipeline
Input modules
Images
Metadata
NamesAndTypes
Groups
Analysis modules
ApplyThreshold
ApplyThreshold
ApplyThreshold
ApplyThreshold
RescaleIntensity
RescaleIntensity
ImageMath
ImageMath
MaskImage
MaskImage
CorrectIlluminationCalculate
CorrectIlluminationCalculate
CorrectIlluminationApply
CorrectIlluminationApply
RescaleIntensity
RescaleIntensity
ApplyThreshold
ApplyThreshold
IdentifyPrimaryObjects
ExpandOrShrinkObjects
GrayToColor
GrayToColor
GrayToColor
GrayToColor
GrayToColor
GrayToColor
OverlayOutlines
OverlayOutlines
OverlayOutlines
Tile
Tile
SaveImages
SaveImages
MeasureObjectSizeShape
MeasureObjectIntensity
ExportToSpreadsheet
Output
View output settings
Adjust modules: + - ^ v
Start Test Mode
Analyze Images
Module notes
Module settings (Tile #34)
Select an input image reconstructed_Nuclear_ThreshCD3_staining (from GrayToColor #29)
Name the output image TiledImage
Tile assembly method Within cycles
Automatically calculate number of rows? Yes No
Final number of rows 1
Automatically calculate number of columns? Yes No
Final number of columns 3
Image corner to begin tiling top left
Direction to begin tiling row
Use meander mode? Yes No
Select an additional image to tile overlay_threshstaining_SegM (from OverlayOutlines #33)
Remove above image
Select an additional image to tile overlay_threshCD3_SegM (from OverlayOutlines #32)
Remove above image
Add another image
Found 4 image sets

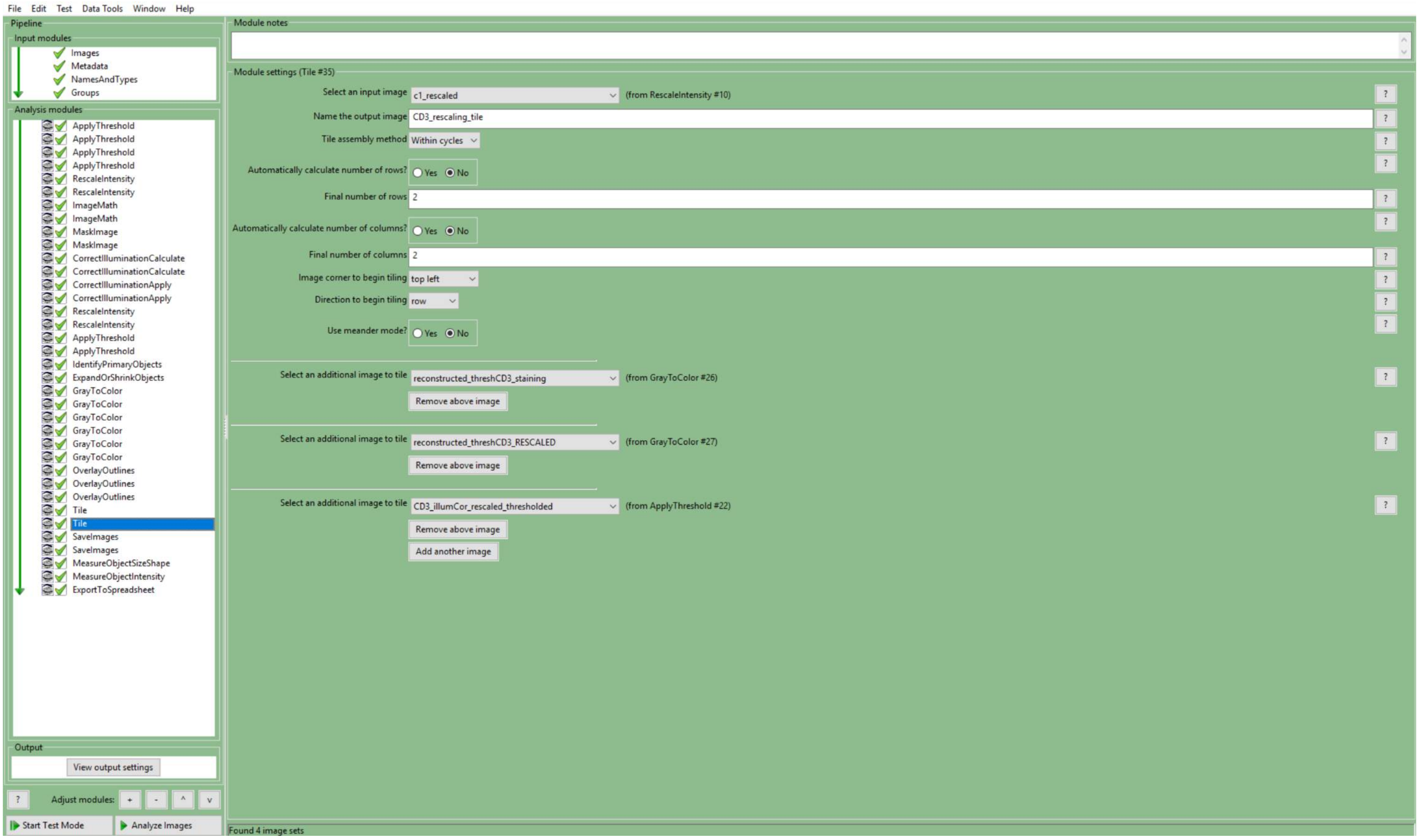
File Edit Test Data Tools Window Help
Pipeline
Input modules
Images
Metadata
NamesAndTypes
Groups
Analysis modules
ApplyThreshold
ApplyThreshold
ApplyThreshold
ApplyThreshold
RescaleIntensity
RescaleIntensity
ImageMath
ImageMath
MaskImage
MaskImage
CorrectIlluminationCalculate
CorrectIlluminationCalculate
CorrectIlluminationApply
CorrectIlluminationApply
RescaleIntensity
RescaleIntensity
ApplyThreshold
ApplyThreshold
IdentifyPrimaryObjects
ExpandOrShrinkObjects
GrayToColor
GrayToColor
GrayToColor
GrayToColor
GrayToColor
GrayToColor
OverlayOutlines
OverlayOutlines
OverlayOutlines
Tile
Tile
SaveImages
SaveImages
MeasureObjectSizeShape
MeasureObjectIntensity
ExportToSpreadsheet
Output
View output settings
Adjust modules:
Start Test Mode
Analyze Images
Module notes
Module settings (Tile #35)
Select an input image
c1_rescaled
(from RescaleIntensity #10)
Name the output image
CD3_rescaling_tile
Tile assembly method
Within cycles
Automatically calculate number of rows?
Yes No
Final number of rows
2
Automatically calculate number of columns?
Yes No
Final number of columns
2
Image corner to begin tiling
top left
Direction to begin tiling
row
Use meander mode?
Yes No
Select an additional image to tile
reconstructed_threshCD3_staining
(from GrayToColor #26)
Remove above image
Select an additional image to tile
reconstructed_threshCD3_RESCALED
(from GrayToColor #27)
Remove above image
Select an additional image to tile
CD3_illumCor_rescaled_thresholded
(from ApplyThreshold #22)
Remove above image
Add another image
Found 4 image sets

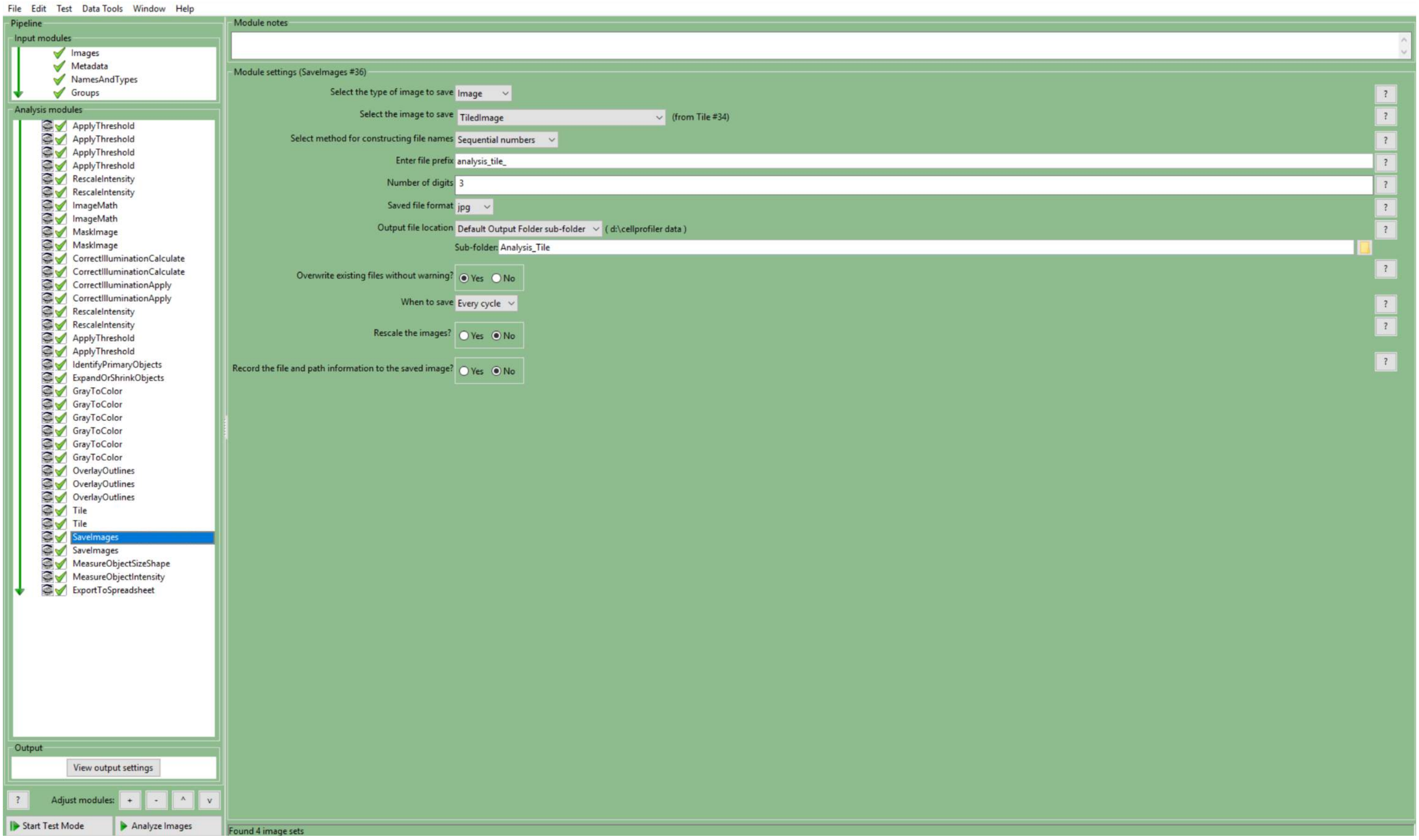
File Edit Test Data Tools Window Help
Pipeline
Input modules
Images
Metadata
NamesAndTypes
Groups
Analysis modules
ApplyThreshold
ApplyThreshold
ApplyThreshold
ApplyThreshold
RescaleIntensity
RescaleIntensity
ImageMath
ImageMath
MaskImage
MaskImage
CorrectIlluminationCalculate
CorrectIlluminationCalculate
CorrectIlluminationApply
CorrectIlluminationApply
RescaleIntensity
RescaleIntensity
ApplyThreshold
ApplyThreshold
IdentifyPrimaryObjects
ExpandOrShrinkObjects
GrayToColor
GrayToColor
GrayToColor
GrayToColor
GrayToColor
GrayToColor
OverlayOutlines
OverlayOutlines
OverlayOutlines
Tile
Tile
SaveImages
SaveImages
MeasureObjectSizeShape
MeasureObjectIntensity
ExportToSpreadsheet
Output
View output settings
Adjust modules:
Start Test Mode
Analyze Images
Module notes
Module settings (SaveImages #36)
Select the type of image to save Image
Select the image to save TiledImage (from Tile #34)
Select method for constructing file names Sequential numbers
Enter file prefix analysis_tile_
Number of digits 3
Saved file format jpg
Output file location Default Output Folder sub-folder (d:\cellprofiler data)
Sub-folder: Analysis_Tile
Overwrite existing files without warning? Yes No
When to save Every cycle
Rescale the images? Yes No
Record the file and path information to the saved image? Yes No
Found 4 image sets

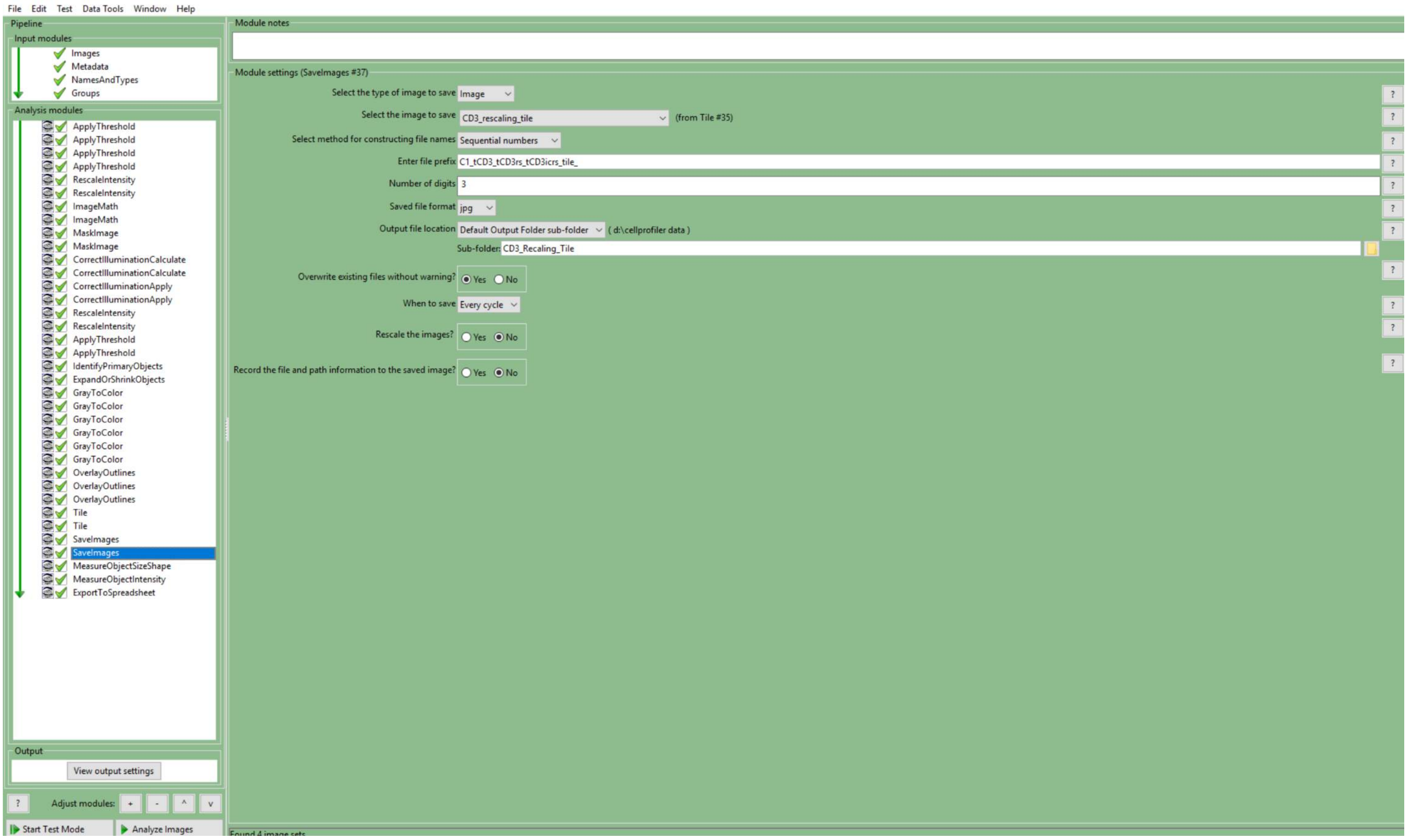
File Edit Test Data Tools Window Help
Pipeline
Input modules
Images
Metadata
NamesAndTypes
Groups
Analysis modules
ApplyThreshold
ApplyThreshold
ApplyThreshold
ApplyThreshold
RescaleIntensity
RescaleIntensity
ImageMath
ImageMath
MaskImage
MaskImage
CorrectIlluminationCalculate
CorrectIlluminationCalculate
CorrectIlluminationApply
CorrectIlluminationApply
RescaleIntensity
RescaleIntensity
ApplyThreshold
ApplyThreshold
IdentifyPrimaryObjects
ExpandOrShrinkObjects
GrayToColor
GrayToColor
GrayToColor
GrayToColor
GrayToColor
GrayToColor
OverlayOutlines
OverlayOutlines
OverlayOutlines
Tile
Tile
SaveImages
SaveImages
MeasureObjectSizeShape
MeasureObjectIntensity
ExportToSpreadsheet
Output
View output settings
Adjust modules:
Start Test Mode
Analyze Images
Module notes
Module settings (SaveImages #37)
Select the type of image to save Image
Select the image to save CD3_rescaling_tile (from Tile #35)
Select method for constructing file names Sequential numbers
Enter file prefix C1_tCD3_tCD3rs_tCD3icrs_tile_
Number of digits 3
Saved file format jpg
Output file location Default Output Folder sub-folder (d:\cellprofiler data)
Sub-folder: CD3_Recaling_Tile
Overwrite existing files without warning? Yes No
When to save Every cycle
Rescale the images? Yes No
Record the file and path information to the saved image? Yes No
Found 4 image sets

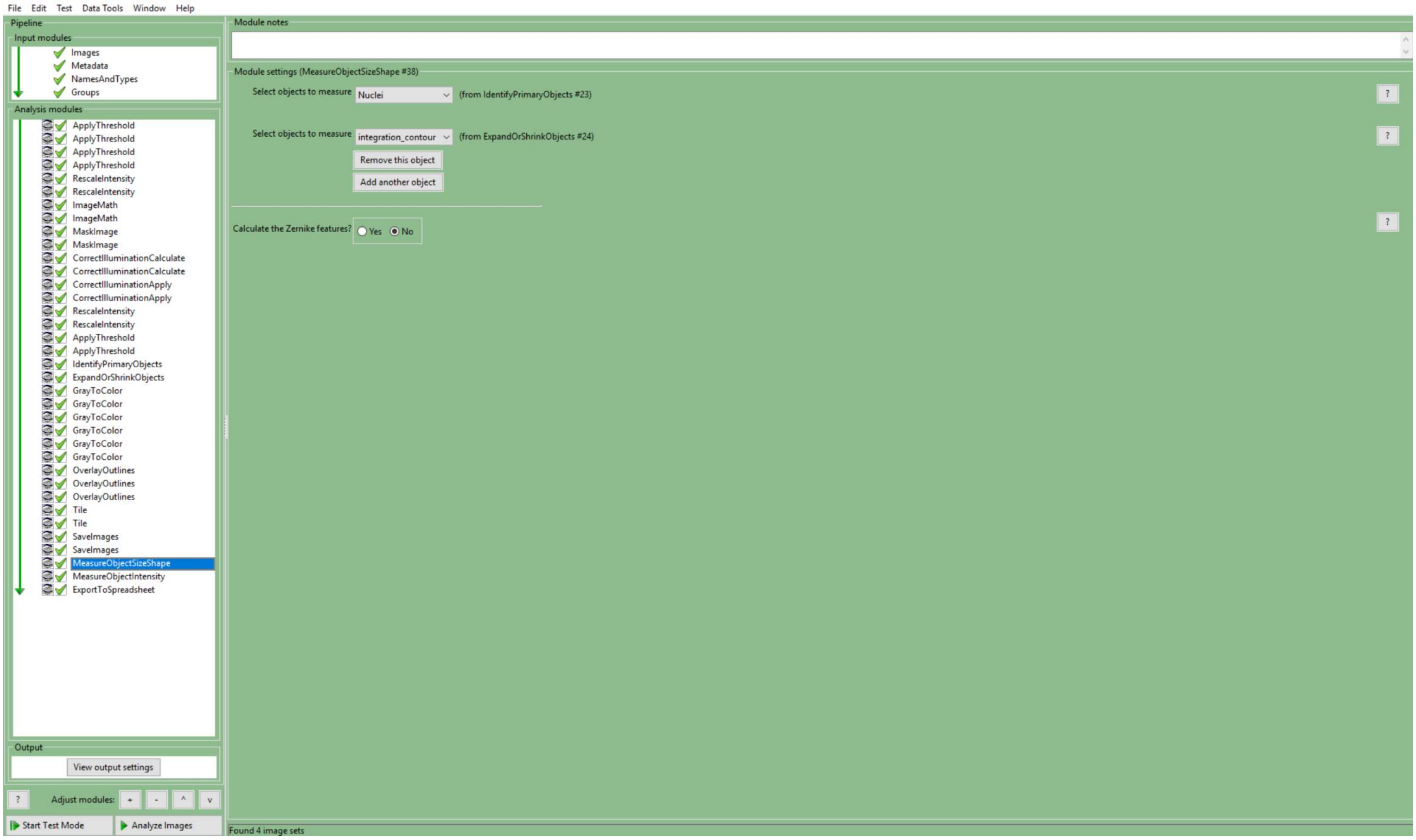
File Edit Test Data Tools Window Help
Pipeline
Input modules
Images
Metadata
NamesAndTypes
Groups
Analysis modules
ApplyThreshold
ApplyThreshold
ApplyThreshold
ApplyThreshold
RescaleIntensity
RescaleIntensity
ImageMath
ImageMath
MaskImage
MaskImage
CorrectIlluminationCalculate
CorrectIlluminationCalculate
CorrectIlluminationApply
CorrectIlluminationApply
RescaleIntensity
RescaleIntensity
ApplyThreshold
ApplyThreshold
IdentifyPrimaryObjects
ExpandOrShrinkObjects
GrayToColor
GrayToColor
GrayToColor
GrayToColor
GrayToColor
GrayToColor
OverlayOutlines
OverlayOutlines
OverlayOutlines
Tile
Tile
SaveImages
SaveImages
MeasureObjectSizeShape
MeasureObjectIntensity
ExportToSpreadsheet
Output
View output settings
Adjust modules:
Start Test Mode
Analyze Images
Module notes
Module settings (MeasureObjectSizeShape #38)
Select objects to measure
Nuclei
(from IdentifyPrimaryObjects #23)
Select objects to measure
integration_contour
(from ExpandOrShrinkObjects #24)
Remove this object
Add another object
Calculate the Zernike features?
Yes
No
Found 4 image sets

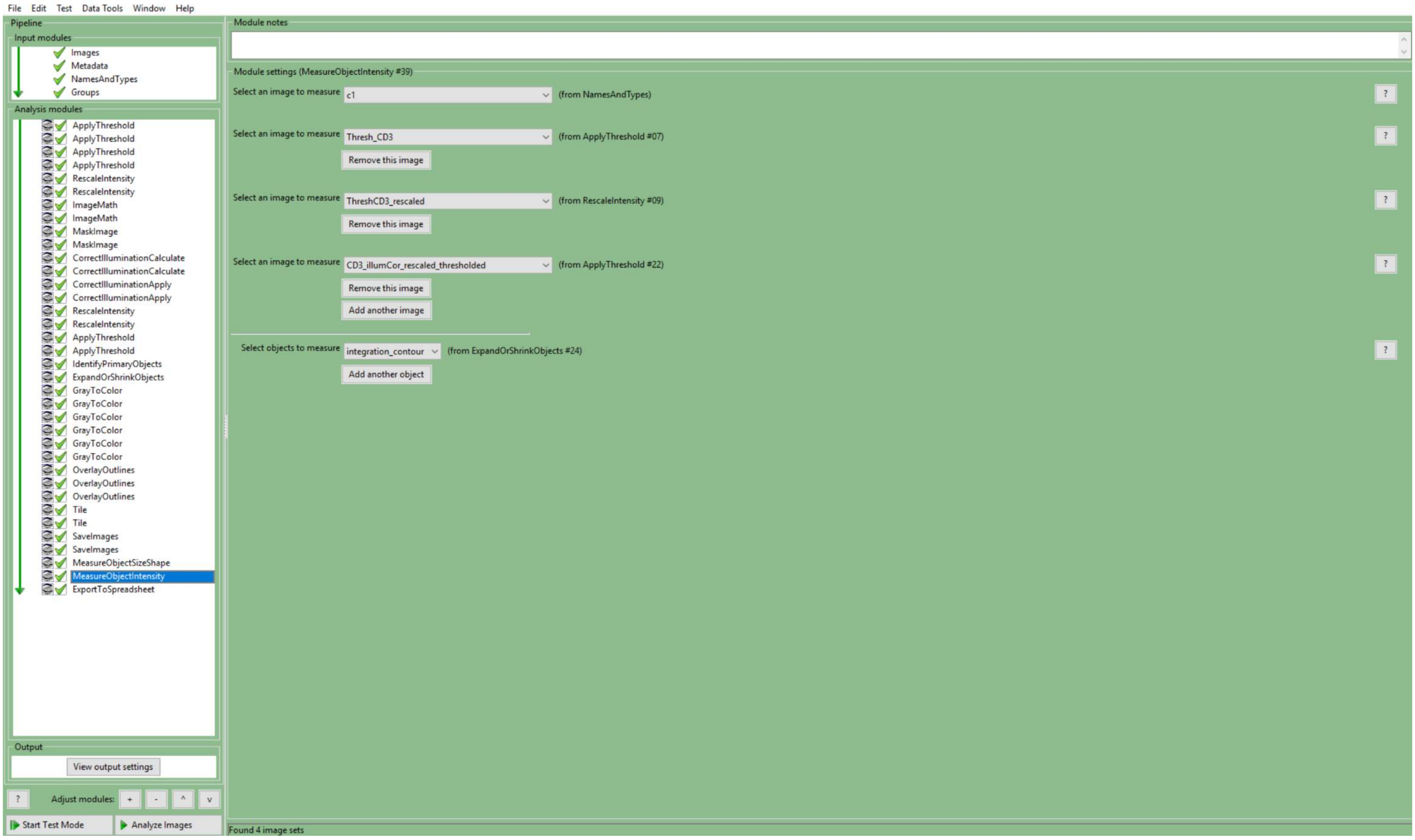
File Edit Test Data Tools Window Help
Pipeline
Input modules
Images
Metadata
NamesAndTypes
Groups
Analysis modules
ApplyThreshold
ApplyThreshold
ApplyThreshold
ApplyThreshold
RescaleIntensity
RescaleIntensity
ImageMath
ImageMath
MaskImage
MaskImage
CorrectIlluminationCalculate
CorrectIlluminationCalculate
CorrectIlluminationApply
CorrectIlluminationApply
RescaleIntensity
RescaleIntensity
ApplyThreshold
ApplyThreshold
IdentifyPrimaryObjects
ExpandOrShrinkObjects
GrayToColor
GrayToColor
GrayToColor
GrayToColor
GrayToColor
GrayToColor
OverlayOutlines
OverlayOutlines
OverlayOutlines
Tile
Tile
SaveImages
SaveImages
MeasureObjectSizeShape
MeasureObjectIntensity
ExportToSpreadsheet
Output
View output settings
Adjust modules:
Start Test Mode
Analyze Images
Module notes
Module settings (MeasureObjectIntensity #39)
Select an image to measure
c1
(from NamesAndTypes)
Select an image to measure
Thresh_CD3
(from ApplyThreshold #07)
Remove this image
Select an image to measure
ThreshCD3_rescaled
(from RescaleIntensity #09)
Remove this image
Select an image to measure
CD3_illumCor_rescaled_thresholded
(from ApplyThreshold #22)
Remove this image
Add another image
Select objects to measure
integration_contour
(from ExpandOrShrinkObjects #24)
Add another object
Found 4 image sets

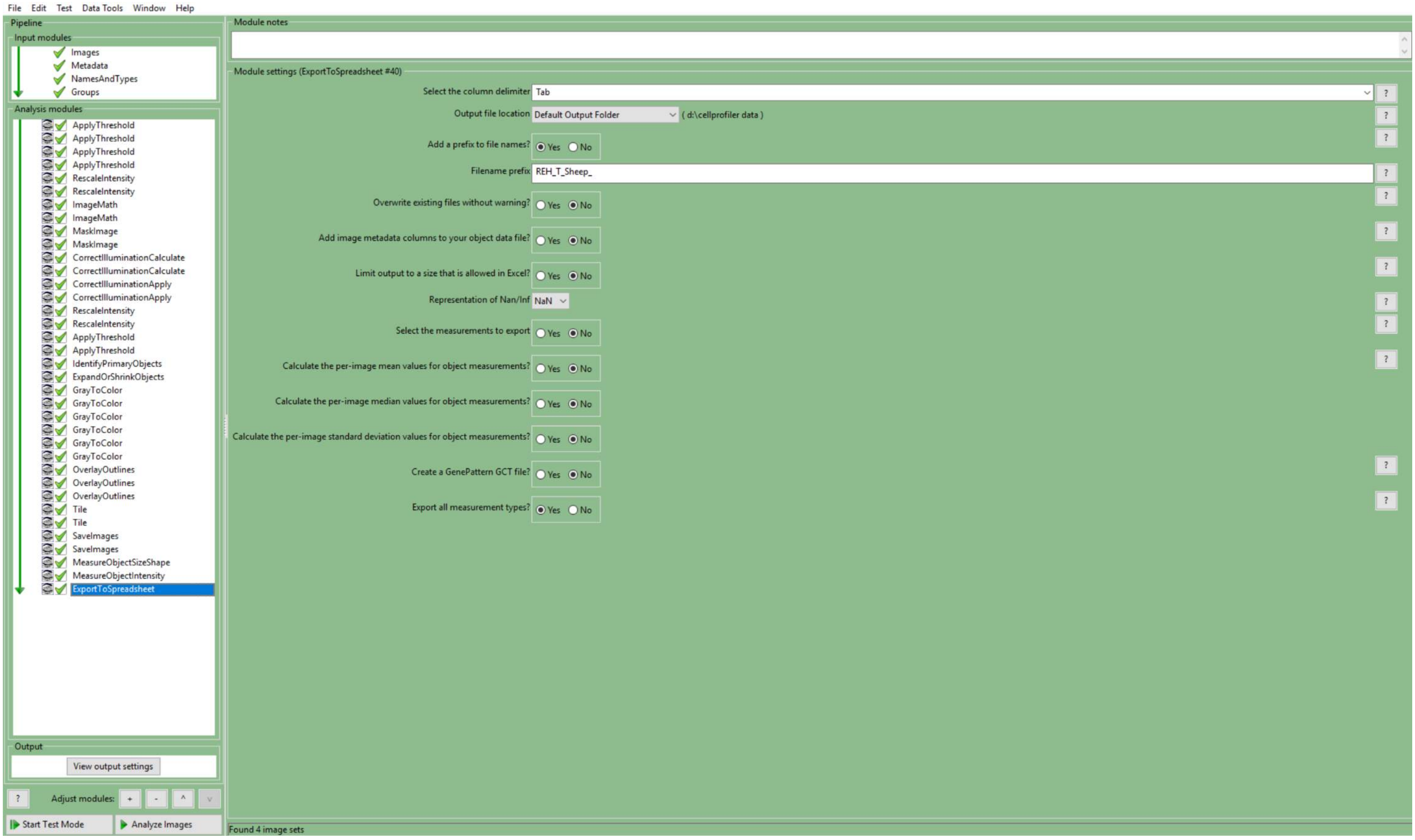
File Edit Test Data Tools Window Help
Pipeline
Input modules
Images
Metadata
NamesAndTypes
Groups
Analysis modules
ApplyThreshold
ApplyThreshold
ApplyThreshold
ApplyThreshold
RescaleIntensity
RescaleIntensity
ImageMath
ImageMath
MaskImage
MaskImage
CorrectIlluminationCalculate
CorrectIlluminationCalculate
CorrectIlluminationApply
CorrectIlluminationApply
RescaleIntensity
RescaleIntensity
ApplyThreshold
ApplyThreshold
IdentifyPrimaryObjects
ExpandOrShrinkObjects
GrayToColor
GrayToColor
GrayToColor
GrayToColor
GrayToColor
GrayToColor
OverlayOutlines
OverlayOutlines
OverlayOutlines
Tile
Tile
SaveImages
SaveImages
MeasureObjectSizeShape
MeasureObjectIntensity
ExportToSpreadsheet
Output
View output settings
Adjust modules:
Start Test Mode
Analyze Images
Module notes
Module settings (ExportToSpreadsheet #40)
Select the column delimiter Tab
Output file location Default Output Folder (d:\cellprofiler data)
Add a prefix to file names? Yes No
Filename prefix REH_T_Sheep_
Overwrite existing files without warning? Yes No
Add image metadata columns to your object data file? Yes No
Limit output to a size that is allowed in Excel? Yes No
Representation of Nan/Inf NaN
Select the measurements to export Yes No
Calculate the per-image mean values for object measurements? Yes No
Calculate the per-image median values for object measurements? Yes No
Calculate the per-image standard deviation values for object measurements? Yes No
Create a GenePattern GCT file? Yes No
Export all measurement types? Yes No
Found 4 image sets
